# Sensitivity Analysis in Photodynamics: How Does the Electronic Structure Control *cis*-Stilbene Photodynamics?

**DOI:** 10.1021/acs.jctc.4c01008

**Published:** 2024-12-12

**Authors:** Tomáš Jíra, Jiří Janoš, Petr Slavíček

**Affiliations:** Department of Physical Chemistry, University of Chemistry and Technology, Technická 5, Prague 6 16628, Czech Republic

## Abstract

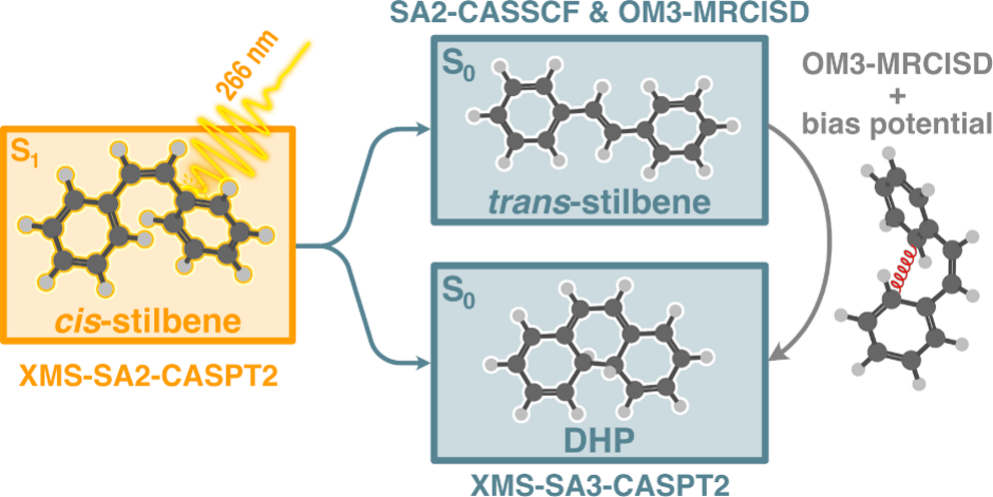

The techniques of computational photodynamics are increasingly employed to unravel reaction mechanisms and interpret experiments. However, misinterpretations in nonadiabatic dynamics caused by inaccurate underlying potentials are often difficult to foresee. This work focuses on revealing the systematic errors in the nonadiabatic simulations due to the underlying potentials and suggests a thrifty approach to evaluate the sensitivity of the simulations to the potential. This issue is exemplified in the photochemistry of *cis*-stilbene, where similar experimental outcomes have been differently interpreted based on the electronic structure methods supporting nonadiabatic dynamics. We examine the predictions of *cis*-stilbene photochemistry using trajectory surface hopping methods coupled with various electronic structure methods (OM3-MRCISD, SA2-CASSCF, XMS-SA2-CASPT2, and XMS-SA3-CASPT2) and assess their ability to interpret experimental observations. While the excited-state lifetimes and calculated photoelectron spectra show consistency with experiments, the reaction quantum yields vary significantly: either completely suppressing cyclization or isomerization. Intriguingly, analyzing stationary points on the potential energy surface does not hint at any major discrepancy, making the electronic structure methods seemingly reliable when treated separately. We show that performing an ensemble of simulations with different potentials provides an estimate of the electronic structure sensitivity. However, this ensemble approach is costly. Thus, we propose running nonadiabatic simulations with an external bias at a resource-efficient underlying potential (semiempirical or machine-learned) for the sensitivity analysis. We demonstrate this approach using a semiempirical OM3-MRCISD method with a harmonic bias toward cyclization.

## 1 Introduction

Nonadiabatic dynamics simulations are increasingly becoming a standard in the theoretical chemistry toolkit, yet their reliability and best practices remain topics of ongoing debate within the field.^1^ Recent discourse has underscored the sensitivity of these simulations to both electronic structure and quantum dynamics algorithms, revealing significant disparities, particularly across different electronic structure methodologies.^2−9^ This sensitivity was starkly highlighted in a recent theoretical prediction challenge, where various research teams attempted to forecast the outcomes of ultrafast electron diffraction experiments on cyclobutanone before their execution.^10^ The resulting theoretical predictions exhibited a broad spectrum of lifetimes and quantum yields, thereby challenging the predictive capacity of *ab initio* computational photodynamics.

Seemingly, the standards of the photodynamics simulations need to be re-evaluated. Building upon this broader dialogue, we focus here on the possible pathways to analyze the sensitivity of the nonadiabatic simulations to the underlying electronic structure. The obvious approach to estimating the systematic uncertainty of the simulations is the use of an ensemble of different methods. We demonstrate this approach in the example of *cis*-stilbene photoisomerization. This strategy is, however, computationally demanding, and we propose a thrifty alternative based on the addition of a perturbing potential into the simulations. We also discuss the possibility of simulation refinement with the experimental constraints.

**Figure 1:**
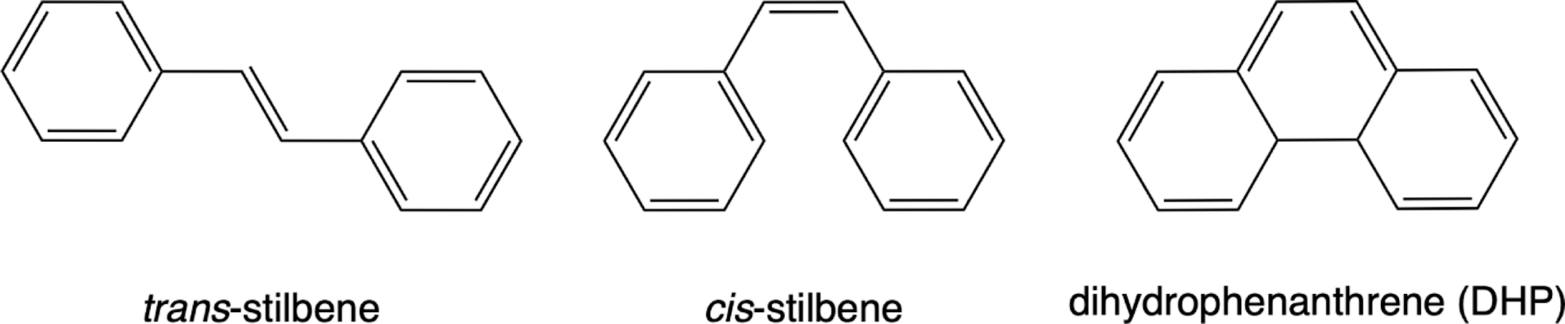
Chemical formulas of *trans*-stilbene, *cis*-stilbene, and 4a,4b-dihydrophenanthrene.

Stilbene stands out as an exceptionally suitable molecule for such an investigation. Notably stable, it does not fragment upon photoisomerization, exhibiting primary photodynamics within hundreds of femtoseconds. In its ground state, the molecule exists in two isomeric configurations: *cis*-stilbene (also known as *Z*-stilbene in systematic nomenclature) and *trans*-stilbene (or *E*-stilbene), as illustrated in Figure 1. *Trans*-stilbene represents the global minimum on the potential energy surface, being approximately 8.27 kJ/mol more stable than *cis*-stilbene (as calculated at the XMS-SA3-CASPT2(2,2)/cc-pVDZ level). Under normal conditions, *trans*-stilbene crystallizes while *cis*-stilbene exists in a liquid state at ambient temperature and pressure. Another noteworthy stable yet energetically disadvantaged isomer of stilbene is 4a,4b-dihydrophenanthrene (DHP), which can be generated photochemically.^11−15^ Both stable isomers of stilbene exhibit strong absorption in the UV region owing to the optically allowed π → π* transition. Upon excitation, *trans*-stilbene persists in the excited state for tens of picoseconds, probably trapped in the near-planar S_1_ minimum. In contrast, *cis*-stilbene undergoes decay over several hundreds of femtoseconds, an interesting phenomenon, given the relatively minor energy disparity between the two isomers.^16−20^

From the perspective of this work, product branching is of special interest. The insights on *cis*-stilbene photochemistry emerged already from classical photochemical studies, providing an abundance of experimental quantum yields and lifetimes in gas and liquid phases and various solvents; see Table 1 for a summary of measured data. Further experiments based on time-resolved spectroscopies elucidated the mechanism and atomic details underlying the photoisomerization or cyclization.^21−28^ The pulse shape effect on quantum yields was also investigated experimentally.^29^ This vast experimental data collection was supplemented by extensive computational studies. A range of approaches, including time-dependent density functional theory,^30,31^ multireference wave function methods^32−34^ improved with second-order perturbation theory,^23,35^ semiempirical methods,^36^ and molecular mechanics valence bond method,^37^ were employed in the scrutiny of *cis*-stilbene photochemistry. Despite the impressive array of experimental and theoretical methodologies, the picture of *cis*-stilbene’s photodynamics is still not converged.

**Table 1:** Experimental Measurement of the *cis*-Stilbene Photoisomerization Quantum Yields ϕ_photoisom._ to *trans*-Stilbene, Cyclization Quantum Yields ϕ_cyc._ to DHP, and the Excited-State Lifetimes τ

environment	ϕ_photoisom._	ϕ_cyc._	τ (fs)
polar solvents	0.35–0.39^38,39^	0.05–0.08^38^	380–500^38^
nonpolar solvents	0.32–0.35^38^	0.15–0.19^38^	1000–1650^38^
liquid phase			1200^36^
gas phase	0.04^23^	0.41^23^	320–500^17,23,36,40,41^

From the large collection of works presented above, we will single out the three most recent and detailed works.^21,23,36^ These works present cutting-edge time-resolved photoelectron spectroscopy experiments combined with high-level theoretical modeling that brought comprehensive accounts on time scales, quantum yields, and the mechanism of *cis*-stilbene photochemistry. However, the interpretations of the measured data, which are strongly supported by the nonadiabatic dynamics simulations, differ significantly. All three works rely on a different electronic structure method, in all three cases well justified from static investigations of potential energy surfaces or literature, yet the forecasted quantum yields are discernibly inconsistent. Let us now review them briefly.

Martínez et al. applied the vacuum ultraviolet time-resolved photoelectron spectroscopy to unmask the *cis*-stilbene phantom state.^21^ The work was supported by previous *ab initio* multiple spawning simulations in conjunction with a complete active space self-consistent field wave function to simulate the process, revealing a time scale of 520 fs and a minor yield of DHP formation of approximately 4%.^32^

Wörner et al. conducted the experiments in both the gas and liquid phases (using the liquid microjet technology).^36^ The experiment was backed up by Landau–Zener surface hopping dynamics with the multireference configuration interaction method and semiempirical OM3 Hamiltonian. The simulations predicted 52% photoisomerization quantum yield and no DHP formation, supporting the claims of Martínez et al.

The work by Suzuki et al. reported a similar investigation solely in the gas phase.^23^ Although the excited-state populations inferred from different sets of experiments were comparable, Suzuki et al. suggested a significant formation of DHP in the gas phase (exceeding 40%), accompanied by an almost complete suppression of the photoisomerization channel. This suggestion was mainly inferred from nonadiabatic dynamics on complete active space second-order perturbation theory potential energy surfaces.

The summary above demonstrates that (i) the interpretation of time-resolved photoelectron spectroscopy still heavily relies on theoretical support, and (ii) all of the theoretical predictions built on only one electronic structure method for dynamics remain blind to their sensitivity, even though the given electronic structure method was benchmarked on static structures important for the mechanism. If the calculated observables are not far from the experiments, distinct interpretations can be accepted without testing a *counter-hypothesis*. This observation prompts a critical question: to what extent can contemporary theory effectively guide ultrafast time-resolved experiments, and how reliable are these predictions?

In this work, we closely inspect the three electronic structure methods used for nonadiabatic dynamics in the aforementioned works^21,23,36^ and demonstrate the importance of an ensemble of electronic structure methods used in nonadiabatic dynamics, even when the standard static examination of their potential energy surfaces does not reveal major discrepancies. We propose that the sensitivity analysis should become a standard component of photodynamics simulations where a diverse portfolio of electronic structure methods is employed. While the use of an ensemble of electronic structure methods is a viable strategy, leading to improved results,^42^ it is not always practical. Hence, we advocate for exploring cost-effective alternatives for conducting this sensitivity analysis, such as a bias potential.

## 2 Methods

### 2.1. Electronic Structure Methods

For the ground-state sampling, we employed the Møller–Plesset perturbation theory of second order (MP2) in combination with a 6-31g* basis. Molecular optimization and frequency analysis were done using Gaussian 09, Revision D.01.^43^

For dynamical simulations and mapping of the potential energy surfaces, we use three different multireference approaches: state-averaged complete active space self-consistent field approach (SA-CASSCF), extended multistate complete active space perturbation theory (XMS-CASPT2), and a multireference configuration interaction with singles and doubles (MRCISD) method based on the semiempirical OM3 Hamiltonian. The SA-CASSCF and XMS-CASPT2 methods consider the active space of two electrons in two π and π* orbitals, further denoted as (2,2). This choice was motivated by previous studies^21,23^ and the prohibitive cost of larger active spaces. On the other hand, the OM3-MRCISD method is efficient enough to be coupled to the full active space such as (12,17) used in ref (36). A level shift of 0.25 atomic units was applied for the XMS-CASPT2 method. The SA-CASSCF and XMS-CASPT2 calculations were performed in the BAGEL package,^44^ while OM3-MRCISD calculation was performed in the MNDO v7.0 code.^45^ The conical intersections were optimized with the gradient projection algorithm of Bearpark et al.^46^ for SA-CASSCF and XMS-CASPT2 in the BAGEL package and with the penalty function algorithm of Ciminelli et al.^47^ for OM3-MRCISD in the MNDO code.

Two basis sets were applied for SA-CASSCF and XMS-CASPT2: SVP and cc-pVDZ coupled to their counterparts for density fitting, SVP-jkfit and cc-pVDZ-jkfit. The cc-pVDZ basis set was used in previous computational studies,^23^ while the SVP basis set was used to provide us with faster results. Since the difference between them is not large (they both are of double-ζ quality containing the same types of basis functions), we discuss only the cc-pVDZ results in the article and leave the SVP result to the Supporting Information. The OM3-MRCISD uses a parametrized pseudominimal basis set.

### 2.2. Nonadiabatic Dynamics

Since this work focuses on photoisomerization of *cis*-stilbene from the bright S_1_ state, the simulations were set up accordingly. All simulations were initiated in the S_1_ state with geometries sampled from the Wigner distribution around the MP2/6-31g* minimum of *cis*-stilbene, assuming the temperature of 298.15 K and momenta sampled from the Boltzmann distribution. Note that the momentum distribution has, in the present case, only a very limited effect on the dynamics, as we discuss in the Supporting Information. We calculated 500 trajectories with the OM3-MRCISD method (with 1 failed simulation), 236 with CASSCF (with 6 failed simulations), 48 with XMS-SA3-CASPT2/cc-pVDZ (with 0 failed simulations), and 58 with XMS-SA2-CASPT2/cc-pVDZ (with 8 failed simulations). The trajectories that failed in the excited state were excluded from the analysis. We show in the Supporting Information that this choice did not introduce a systematic bias into the analyzed data. We also show explicitly the convergence of the calculated data with the number of trajectories in the Supporting Information. All of the simulations were performed using the fewest switches surface hopping (FSSH) scheme.^48^ The OM3-MRCISD dynamics with an additional bias used the Landau–Zener surface hopping (LZSH) method^49^ for speed up since the simulations do not depend on the particular scheme for nonadiabatic dynamics, see the Supporting Information. For all simulations, the time step was set to 5 au. The total energy was conserved well in the excited state for XMS-SA3-CASPT2 and SA2-CASSCF, with only occasional orbital rotations causing small random jumps in total energy. The energy conservation was typically better than 0.1 eV in the excited state for these methods. The jumps in total energy were more frequent once the molecule reached the ground state, yet at that point, the trajectories had already reached stable ground-state structures. The XMS-SA2-CASPT2 was more prone to orbital rotations and energy jumps in the excited state compared to XMS-SA3-CASPT2. A slight energy drift is observed in the excited state, with energy nonconservation typically on the order of tenths of an eV. However, these jumps only shifted the PES without discernibly altering its shape. The energy discontinuities were more frequent for OM3-MRCISD. This is expected, considering the large active space and the fact that the orbitals are not optimized with a state-averaging procedure. Still, the energy jumps were moderate (below 0.2 eV) and comparably smaller than the energy scale on which the photochemistry occurs. The total energies of all of the simulations are analyzed in the SI. All simulations were performed using our molecular dynamics code ABIN.^50^

### 2.3. Fewest Switches Surface Hopping

The FSSH algorithm provides the expression for the evolution of the electronic amplitudes, denoted as *c*_*k*_(*t*), where *k* is an electronic-state label. The evolution of the amplitudes is described by the following coupled equations
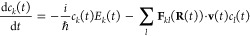
1where *E*_*k*_(*t*) represents the electronic energy of the *k*-th state, **R**(*t*) denotes the nuclear configuration following classical trajectory, **v**(*t*) is the nuclear velocity vector, and **F**_*kl*_ is the nonadiabatic coupling between states *k* and *l*, given by
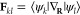
2Utilizing the electronic amplitudes and nonadiabatic couplings, the probability of an electronic transition (hopping) is evaluated as
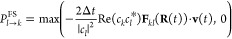
3This probability is calculated at each time step, and if a transition occurs, the gradients used for the dynamics are subsequently altered. To enforce the total energy conservation of the trajectory, the momentum is always rescaled along the nonadiabatic coupling vector upon hopping between the electronic states. To mend the overcoherence issue of FSSH, we applied the decoherence correction suggested by Granucci and Persico with a recommended value of 0.1 au.^51^

### 2.4. Landau–Zener Surface Hopping

In contrast to the FSSH approach, the LZSH scheme is based on an analytically solved model of linearly crossing potential energy surfaces and does not require nonadiabatic couplings for computing the transition probabilities. The hopping probability within the LZSH scheme is evaluated only at time t* when a minimum between the electronic states is encountered along the trajectory. The hopping probability is then expressed as^52^
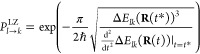
4where Δ*E*_*lk*_ denotes the energy difference between the two electronic states *l* and *k*. To ensure total energy conservation, the momentum is also rescaled upon hopping; however, in this case, it is along the momentum vector, as nonadiabatic couplings are not computed in the LZSH scheme. The LZSH technique was proven to be of comparable quality to FSSH for systems with well-defined conical intersections.^2,49^

### 2.5. Analysis of the Surface Hopping Trajectories

One of the immediate outcomes of the dynamics is time-dependent electronic-state populations. The population of a given state at a time *t* was calculated as a ratio of trajectories in that state and the total number of trajectories alive at the specified time. If the simulation failed after the jump to the ground state, we assumed that the trajectory would remain in the ground state until the end of the simulation and included it in the electronic-state population calculation even after the simulation failed. Since populations can be modeled as a random variable sampled from a binomial distribution (or a sum of variables from the Bernoulli distribution) at each time, we can estimate the confidence intervals using normal distribution as
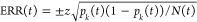
5where *N*(*t*) is the number of trajectories alive at a given time and *p*_*k*_(*t*) is the *k*-th state population. Considering a two-sided 95% confidence interval, we use *z* = 1.96 (the 97.5% quantile of standard normal distribution). To obtain the excited-state lifetime, we fit the populations with a delayed exponential function *p*_fit_ of the form
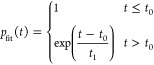
6where *t*_0_ (vibrational relaxation before the decay) and *t*_1_ (exponential decay lifetime) are fitted parameters. The total lifetime is then τ = *t*_0_ + *t*_1_.

**Figure 2:**
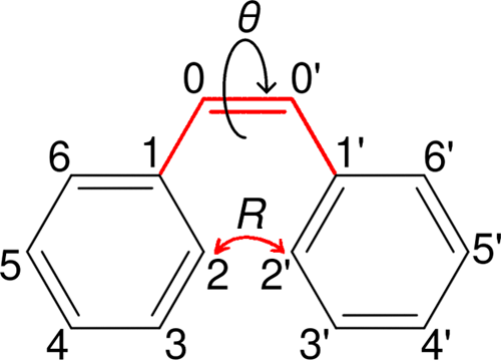
Geometric parameters *R* and θ used for categorizing the *cis*-stilbene, *trans*-stilbene, and DHP.

Moreover, we analyzed the photoproduct population of *cis*-stilbene, *trans*-stilbene, and DHP. The photoproduct populations are calculated as a ratio of trajectories in a specified conformation and the total number of trajectories alive at the specified time. The photoproduct population at the end of simulation is then referred to as the quantum yield. The conformations were categorized using the geometric parameters in Figure 2. The *trans*-stilbene is defined as a conformation with 90° < θ < 270°, while DHP is a conformation with *R* < 1.8 Å. If none of the former conditions are met, then the conformation is categorized as *cis*-stilbene. Both the time-dependent populations and quantum yields were convolved with a Gaussian kernel using a standard deviation of 30 fs.

Finally, we analyzed the hopping geometries employing the multidimensional scaling (MDS) algorithm, which allowed us to visualize geometries in low dimensional space. The MDS algorithm takes the molecular geometries as input (set of 3*N* coordinates) and projects them on low-dimensional reduced coordinates sorted by the variance covered in the initial data. The dimensionality reduction in MDS attempts to preserve distances between the data points and, as such, is suitable for comparing different data sets. The data can then be visualized in an arbitrary dimensional space. In this work, we will focus on the reduction to a 2-dimensional space. A more in-depth description of MDS can be found in refs (2,53).

### 2.6. Modeling the Time-Resolved Photoelectron Spectra

To describe the time-resolved photoelectron spectrum (TRPES), we extracted the photoelectron signals at every 12 fs (500 au) along each trajectory. The ionization energies were calculated for the first 10 ionized states at the OM3-MRCISD(10,7) level, while the intensities of these ionization energies were modeled by taking norms of the corresponding Dyson orbitals (Dyson norms) calculated at the CASSCF(2,2)/SVP level of theory. A histogram was constructed from the ionization energies weighted by their corresponding intensities at the given time, which was then convolved with a two-dimensional Gaussian kernel having a standard deviation σ of 0.1 eV in the energy domain and 22 fs in the time domain. Additionally, a cutoff for convolution is implemented at a value of 4σ. The resulting two-dimensional heat maps the final photoelectron spectra. For a comparison between OM3-MRCISD and CASSCF ionization energies, see the Supporting Information. The error bars of integrals over the photoelectron spectra were estimated using a bootstrapping algorithm. 10,000 samples were generated by randomly choosing a fixed (number of original trajectories) number of trajectories from the full set. The integrals were calculated for each sample, creating a distribution of integral spectra. We then assumed a normal distribution and calculated the error bars as a 95% confidence interval of the obtained distribution.

## 3 Results and Discussion

We first briefly describe the topology of the *cis*-stilbene potential energy surface, focusing on the distinctions between different electronic structure levels. Next, we compare the outcome of dynamical simulations for the different electronic structure methods, with a specific focus on time-resolved photoelectron spectroscopy. Finally, we propose a thrifty approach to investigate the sensitivity of the photodynamical simulations with respect to the electronic structure method used.

### 3.1 Mapping the Potential Energy Surface

**Figure 3:**
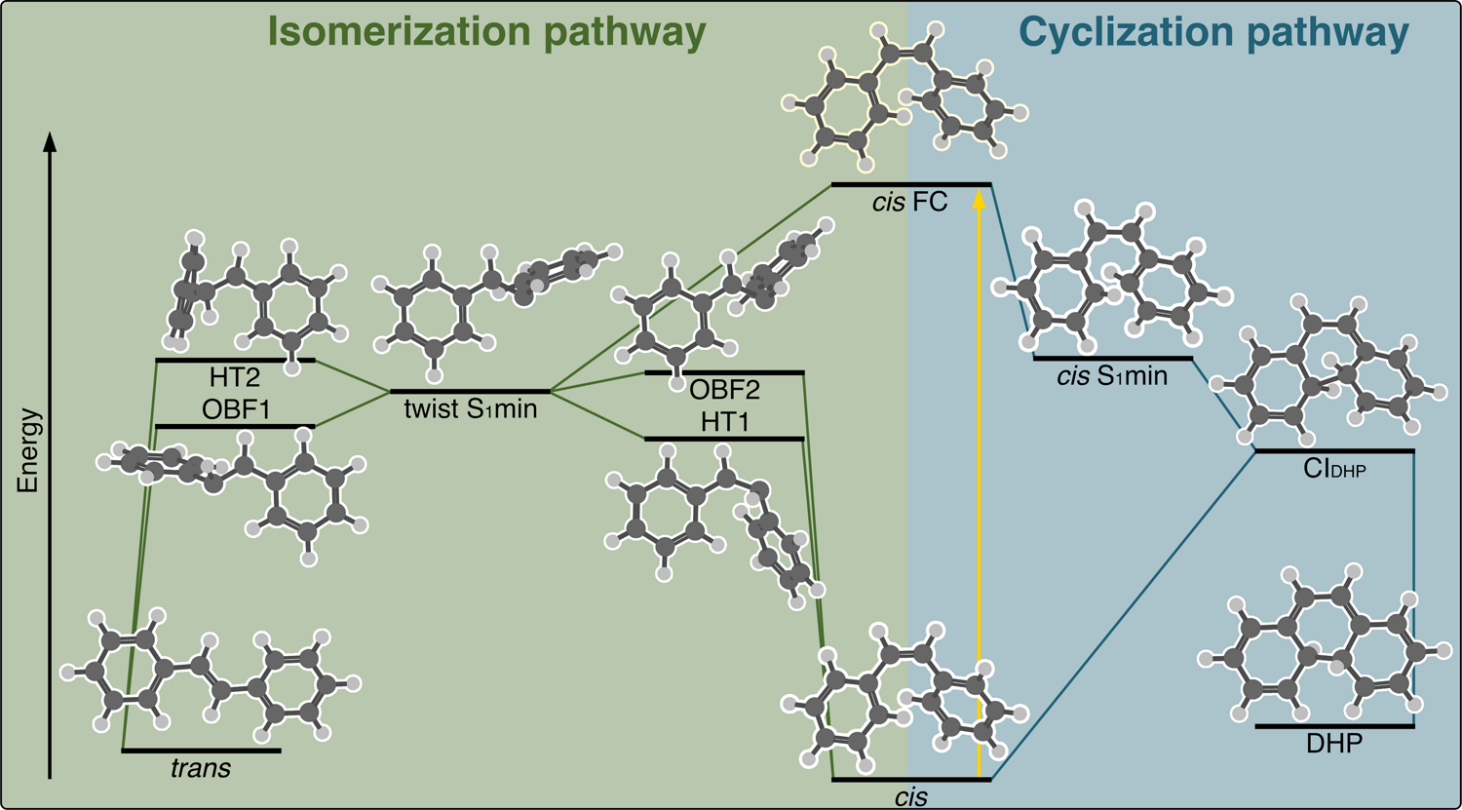
Reaction mechanism of *cis*-stilbene upon excitation to the S_1_ state, depicted as a schematic energy diagram showing energy relations between the important structures on the potential energy surface.

The overall picture of *cis*-stilbene photodynamics is shown in Figure 3. Following the excitation, there are two possible pathways. (i) The cyclization pathway starts by moving toward a shallow *cis* S_1_ minimum characterized by a short distance *R* ≈ 2.0 Å defined in Figure 2. Note that the very existence of this minimum was previously discussed in the literature.^24,54^ The *cis* S_1_ minimum geometry is very close in energy to a conical intersection leading to DHP (*R* ≈ 1.8 Å). In the vicinity of this conical intersection, *cis*-stilbene can transfer its population to the ground state and either produce cyclic DHP or recover the initial *cis* structure. (ii) The second possible pathway—photoisomerization pathway—leads to a twisted geometry with the two rings perpendicular to each other (i.e., geometry halfway between *cis* and *trans* isomers). From this minimum, four different conical intersections are available either via one-bond flip (OBF) or hula-twist (HT): coined OBF1, OBF2, HT1, and HT2 in the literature.^55,56^ We adopt the notation used in the literature that OBF1 and HT2 funnel toward the *trans*-stilbene (denoted as *trans* CIs) while OBF2 and HT1 lead rather to the *cis* isomer (denoted as *cis* CIs). We note that this classification might be a bit arbitrary and all of the conical intersections may lead to both isomers. Nonetheless, all of the conical intersections are energetically accessible, following the S_1_ excitation of *cis*-stilbene, and the molecule then funnels the excited-state wavepacket back to the ground state, back either into the *cis*-stilbene or to the *trans*-stilbene.

**Figure 4:**
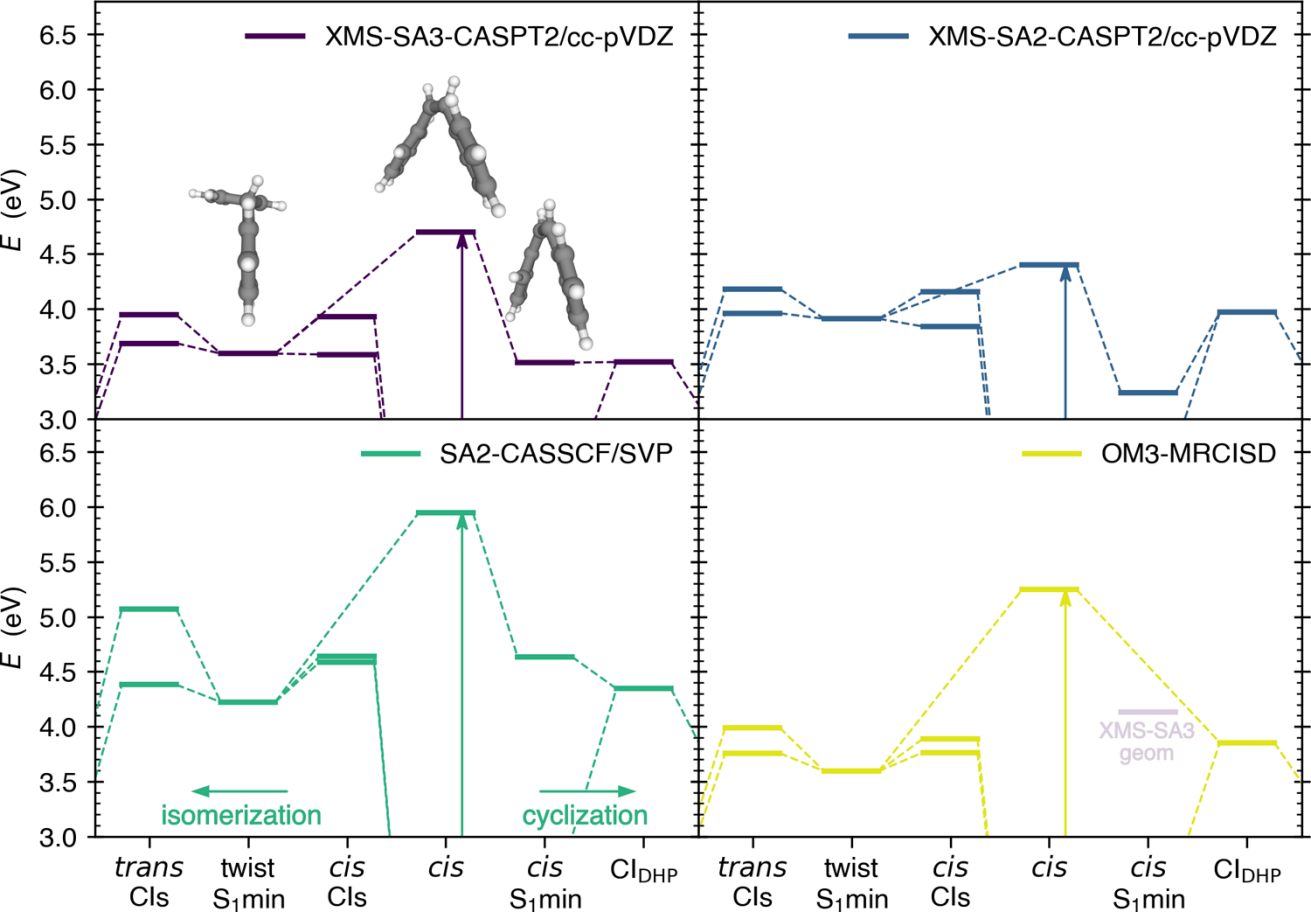
Mapping important points at the potential energy surface of *cis*-stilbene for various electronic structure methods, see Figure 3 for the full schematic energy diagram. The ground-state *cis*-stilbene geometry is set as a reference to 0 eV. We were not able to optimize the *cis* S_1_min geometry for the OM3-MRCISD method, as it always slipped to the conical intersection. Thus, we provide the energy calculated at the XMS-SA3-CASPT2/cc-pVDZ geometry in light purple in the bottom left panel.

We shall now focus on the difference between the electronic structure methods applied for nonadiabatic dynamics of *cis*-stilbene in previous works—SA2-CASSCF, OM3-MRCISD, and XMS-SA3-CASPT2—together with the XMS-SA2-CASPT2 method, representing the dynamically correlated version of the SA2-CASSCF method. Although XMS-SA2-CASPT2 was not used in any previous work, it makes a natural extension of SA2-CASSCF and, as such, appears as a reasonable choice. We zoomed the Franck–Condon point, S_1_ minima, and conical intersections to compare their relative energies and discuss the plausibility of the two pathways introduced above from the energy perspective, see Figure 4. From a general perspective, all of the methods predict both pathways, cyclization and isomerization, energetically accessible. The pictures drawn by XMS-SA3-CASPT2, SA2-CASSCF, and OM3-MRCISD are qualitatively comparable: the cyclization pathway being seemingly barrierless as the DHP conical intersection is below or at the level of the *cis* minimum while the twisted minimum sits below the conical intersections in the photoisomerization pathway. XMS-SA2-CASPT2 stands as an outlier here. The DHP conical intersection is about 0.8 eV above the *cis* minimum, suggesting a discrimination of cyclization. On the other hand, one of the conical intersections in the photoisomerization pathways is discernibly below the twist minimum.

**Figure 5:**
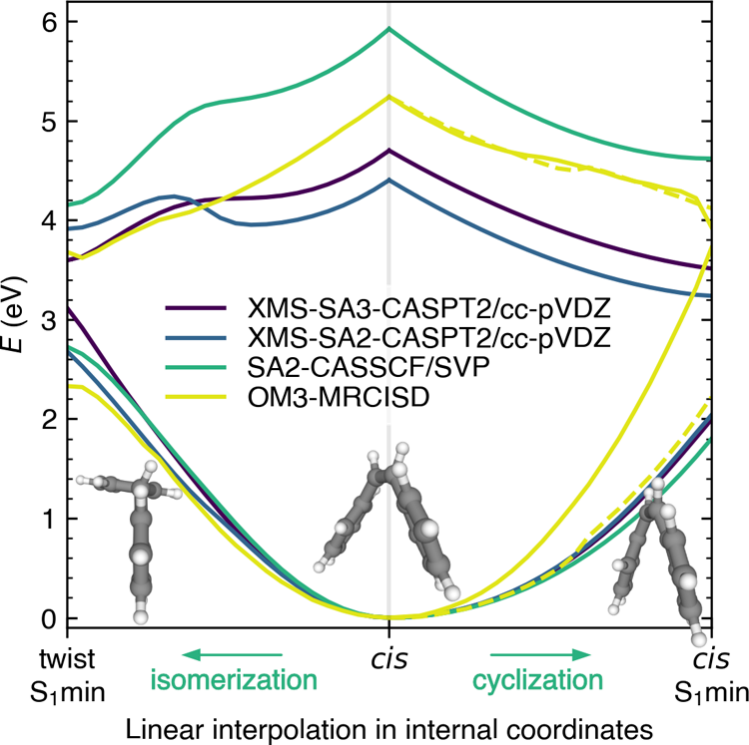
Linear interpolation in internal coordinates between the ground-state *cis*-stilbene geometry, twisted S_1_ minimum, and *cis* S_1_ minimum. All of the geometries were optimized at their respective electronic structure levels. The motion from the *cis* geometry to the *cis* S_1_ minimum is characterized by shortening the distance *R* (defined in Figure 2), while the motion for *cis*-stilbene toward the twisted S_1_ minimum corresponds to prolonging the distance *R*. As we could not optimize the *cis* S_1_ minimum with OM3-MRCISD, we used a geometry close to the DHP conical intersection instead. We also plot the LIIC between the *cis* OM3-MRCISD geometry and the XMS-SA3-CASPT2/cc-pVDZ *cis* S_1_ minimum in the dashed line.

Although energetics in Figure 4 can disclose the energetic accessibility of channels, it contains no information about the direction in which the molecule will be driven after the excitation. To estimate how the molecule will evolve after excitation, we evaluated the potential curves along linear interpolation in internal coordinates between *cis* and *cis* S_1_ minimum, and *cis* and twisted S_1_ minimum, see Figure 5. These LIICs capture both reaction pathways and should help us assess whether any of them will be preferred. All of the methods show a very similar slope toward both reaction pathways without any seeming preference. The only outlier is again the XMS-SA2-CASPT2 method, which exhibits a barrier toward the isomerization, and one could assume that cyclization will be the leading pathway (which is, however, hindered by the energetic barrier of conical intersection). Although this barrier might be as well an artifact of the linear interpolation, the current investigation would probably exclude XMS-SA2-CASPT2 from nonadiabatic dynamics application even though the corresponding CASSCF calculation was used and the choice looks reasonable for an essentially two-state problem.

Scans of potential energy surfaces along LIICs are often used to select an electronic structure method. Although these scans provide more information than isolated points on the potential energy surface, the LIICs are only an approximation of the reaction pathway, ignoring the detailed variations of the potential energy surface. As such, any transition-state barriers predicted by these scans are only estimates of the true barriers. In the end, dynamic calculations are always needed to reveal the true reaction pathways.

### 3.2 Nonadiabatic Dynamics of *cis*-Stilbene: Control by Electronic Structure

The examination of the potential energy surfaces above offers insight into the accessibility of potential reaction pathways and hints at the initial motion upon excitation to the S_1_ state. Our investigation showed no preferential pathway for XMS-SA3-CASPT2, SA2-CASSCF, and OM3-MRCISD—the methods applied in the works of Suzuki et al.,^23^ Martínez et al.,^32^ and Wörner et al.^36^—and we cannot claim any of them to be superior to others and best suitable for dynamics. The only outlier is the XMS-SA2-CASPT2, which we included in the set to test the effect of the stabilizing third state in state averaging of XMS-SA3-CASPT2. Although we would not recommend XMS-SA2-CASPT2 for dynamics, we still performed the dynamics to reveal the disparities.

**Figure 6:**
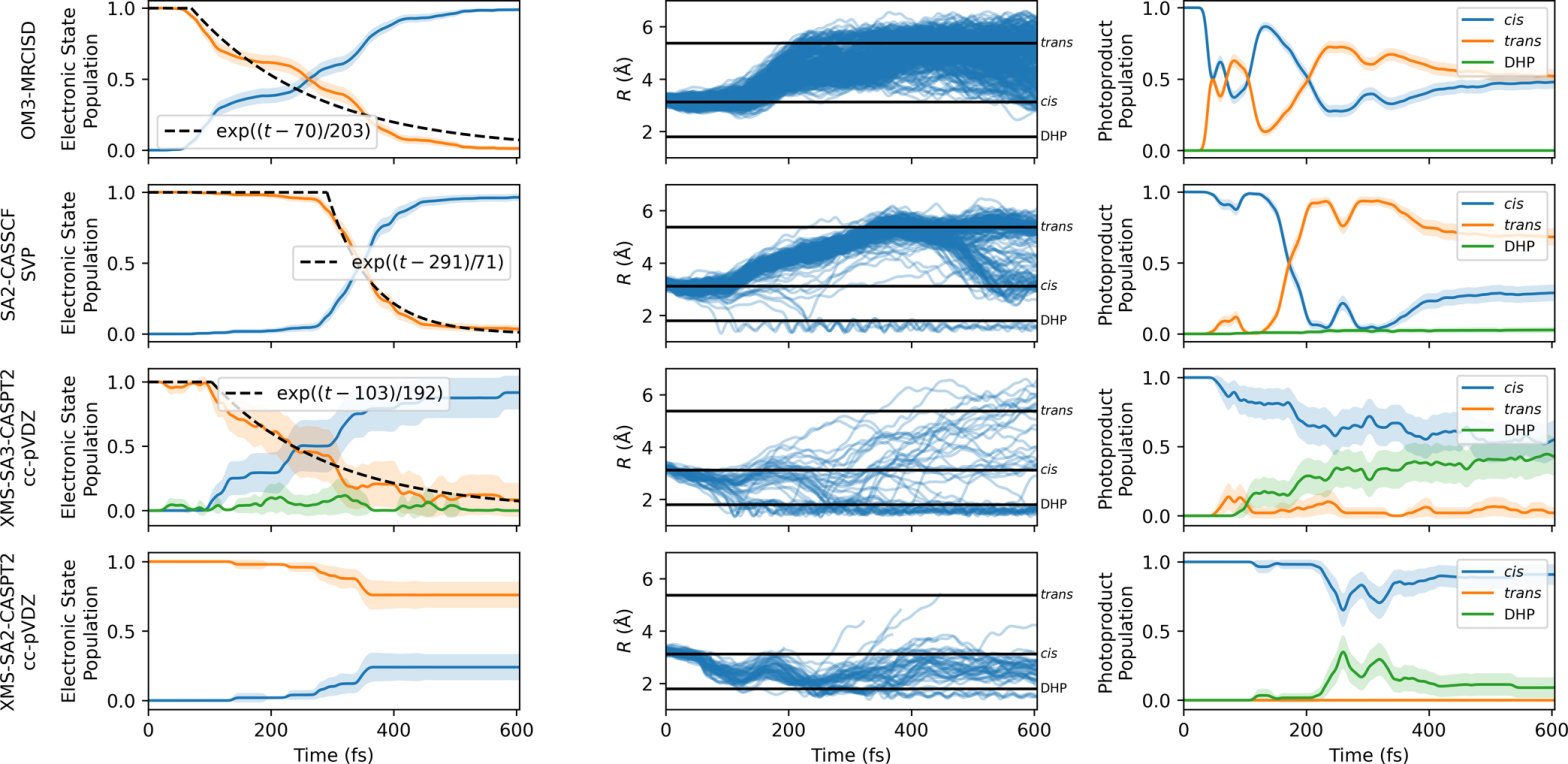
Analysis of nonadiabatic simulations for *cis*-stilbene. The left column shows the time evolution of electronic ground and excited-state populations, with accompanying 95% confidence intervals and a least-squares fit for the excited-state curve. The middle column tracks the distance *R* between the C_2_ and C_2′_ atoms throughout the simulation period. We delineate three distinctive distances: the upper distance, at 5.375 Å, corresponds to the *trans*-stilbene structure; the middle distance, at 3.125 Å, signifies the *cis*-stilbene configuration; and the distance at 1.8 Å represents the DHP state. The right column showcases the *cis*, *trans*, and DHP photoproduct populations during the dynamics. The *trans*-stilbene is defined as a conformation 90° < θ < 270°, where θ is the dihedral angle between atoms C_1_, C_0_, C_0′_, and C_1′_. DHP is a conformation with *R* < 1.8 Å, where *R* is the distance between *C*_2_ and *C*_2′_. Any remaining configurations are classified as *cis*-stilbene. The error bars were calculated according to eq 5.

First, let us examine the excited-state lifetimes, see the first column of Figure 6. The excited-state populations are not directly measurable, yet these quantities can be inferred from time-resolved photoelectron spectroscopic. Gas-phase experiments agree on excited-state lifetimes of about 320–500 fs.^17,23,36,40,41^ The calculated electronic populations (as well as the corresponding experimental data) were best fitted to a delayed exponential function, see formula 6, with a total lifetime of τ. We were able to extract meaningful excited-state lifetimes only for the OM3-MRCISD (τ = 321 ± 10 fs), SA2-CASSCF/SVP (τ = 360 ± 6 fs), and XMS-SA3-CASPT2/cc-pVDZ (τ = 295 ± 10 fs) methods since XMS-SA2-CASPT2 did not decay to the ground state. The calculated values generally appear shorter than in the other theoretical works, yet they are still within the experimental range, see Table 2. This discrepancy may stem from differences in ground-state sampling, with our simulation having more energy at the beginning. While the extracted lifetimes appear similar across methods, the decay profiles differ significantly. For instance, the SA2-CASSCF/SVP method exhibits rapid decay while featuring a relatively long induction period. Conversely, the XMS-SA3-CASPT2/cc-pVDZ method is characterized by a short induction period and a relatively long decay time.

**Table 2:** Calculated Quantum Yields ϕ and Excited-State Lifetimes τ Together with the Experimental Values in Various Solvents and in the Gas Phase^a^

level of theory	ϕ_cis_	ϕ_trans_	ϕ_DHP_	τ (fs)
OM3-MRCISD (LZSH)	0.48 ± 0.05	0.52 ± 0.05	0.00 ± 0.00	321 ± 10
SA2-CASSCF/SVP	0.29 ± 0.06	0.68 ± 0.06	0.03 ± 0.02	360 ± 6
XMS-SA3-CASPT2/cc-pVDZ	0.55 ± 0.14	0.02 ± 0.04	0.43 ± 0.14	295 ± 10
XMS-SA2-CASPT2/cc-pVDZ	0.91 ± 0.07	0.00 ± 0.00	0.09 ± 0.07	
XMS-SA3-CASPT2(2,2)/cc-pVDZ^23^	0.55 ± 0.14	0.04 ± 0.10	0.41 ± 0.14	572 ± 16
OM3-MRCISD(12,17)^36^	0.48	0.52	0.0	≈ 500
SA2-CASSCF(2,2)/6-31G*^32^	0.45 ± 0.04	0.52 ± 0.04	0.04 ± 0.01	520 ± 40
experiment (solvents)^38,39^	0.47–0.59	0.35–0.39	0.05–0.08	380–1650

a The XMS-SA2-CASPT2 simulation did not acquire enough ground-state population for evaluating either quantum yields or the lifetime. The experimental gas-phase quantum yields were omitted as they are not purely experimental quantities but were predicted on the support of simulations with the XMS-SA3-CASPT2(2,2)/cc-pVDZ electronic structure.

The XMS-SA2-CASPT2 method represents very different dynamics. The molecule relaxes in the excited state without closely approaching the conical intersection, resulting in minimal population transfer to the ground state within the simulation time frame. Such behavior is not surprising, referring to the previous section, where we showed the substantial energy gap between the *cis* minimum and the CI_DHP_. It is important to note that while XMS-SA2-CASPT2 and XMS-SA3-CASPT2 visually share the same active space orbitals, the computed energies differ between the two approaches. This difference arises from state-averaging the treatment in the two methods, where XMS-SA2-CASPT2 averages over two states, while XMS-SA3-CASPT2 includes also the third state inactive in the photochemistry. The third state in the state averaging influences the resulting energy landscape, and although the differences in energy are relatively minor on the absolute scale, it leads to variations in predicted dynamics and excited-state behavior.

The differentiation between the various electronic structure methods is further elucidated in the middle panel of Figure 6, which now illustrates the distance *R* between the key carbon atoms of the two benzene rings. Despite the closely matched lifetimes observed (except for XMS-SA2-CASPT2), significant differences in structural evolution manifest between the methods. In both the SA2-CASSCF and OM3-MRCISD simulations, molecules predominantly maintain *cis* configurations initially (based on the distance *R*), gradually transitioning to adopt *trans* geometries. Conversely, all CASPT2 calculations exhibit a shortening of the distance *R* and evolve toward CI_DHP_. Subsequently, XMS-SA3-CASPT2 simulation progresses to the ground state and forms DHP, while XMS-SA2-CASPT2 simulation remains confined in the excited S_1_ basin without culminating in the final product formation.

This narrative gains further depth when considering time-dependent photoproduct populations. We focus on three primary products of photoreaction: *cis*-stilbene, *trans*-stilbene, and DHP. Again, simulations conducted at different levels of theory yield significantly divergent results. The quantum yield of DHP remains negligible with the OM3-MRCISD, SA2-CASSCF, and XMS-SA2-CASPT2 levels of theory. Conversely, the XMS-SA3-CASPT2 methods exhibit a substantial quantum yield of DHP and almost no *trans*-stilbene. Considering that the XMS-SA2-CASPT2 simulation did not show significant deexcitation, it is unsurprising that the majority of products consist of *cis*-stilbene. Specific values, along with experiment comparisons, can be seen in Table 2.

**Figure 7:**
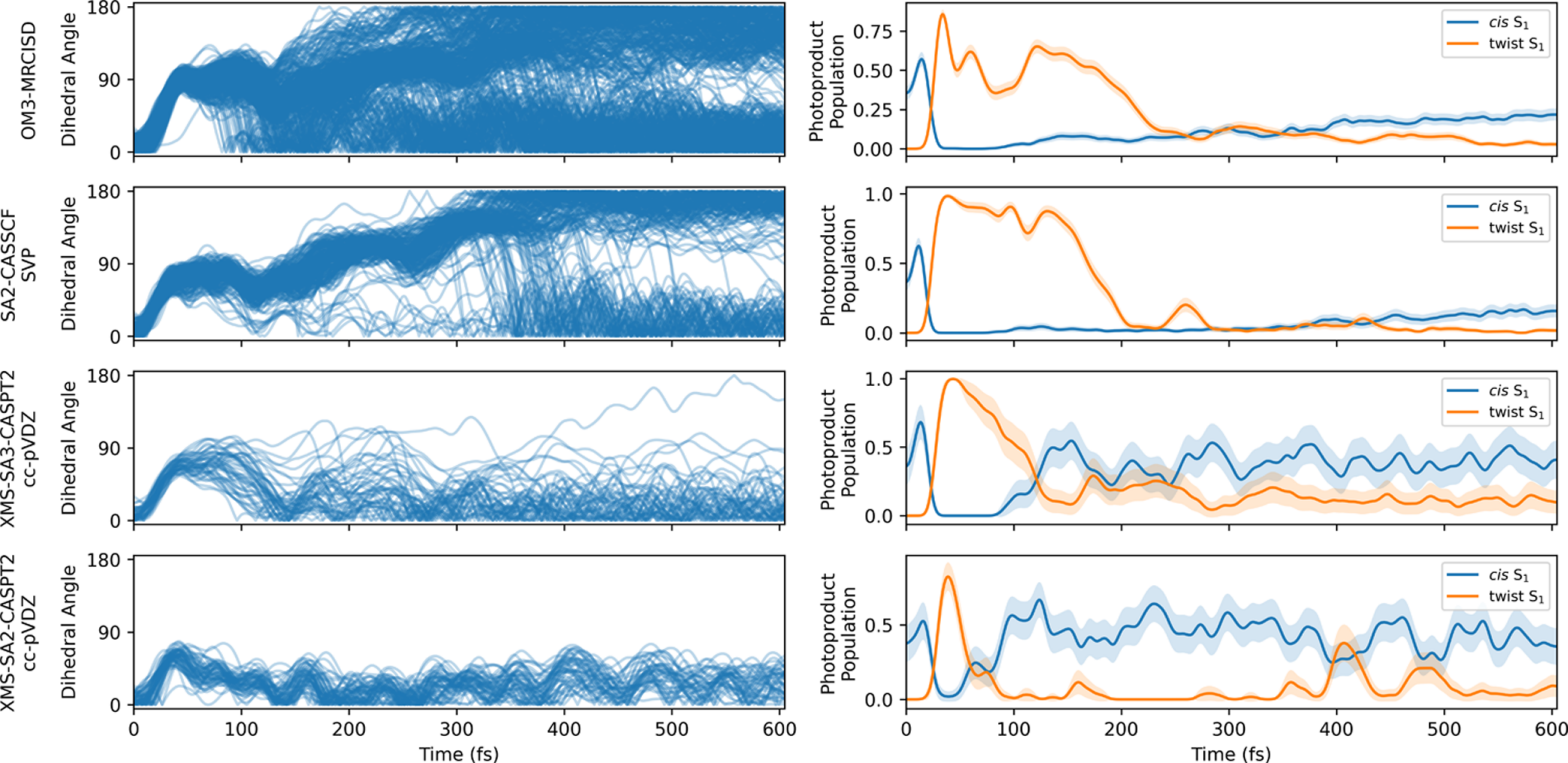
Analysis of nonadiabatic simulations for *cis*-stilbene. The left column tracks the dihedral angle θ between C_1_, C_0_, C_0′_, and C_1′_ atoms throughout the simulation period. The right column showcases the ratios of *cis* and twist geometries during the simulation period. Twist conformation is defined as a conformation with 50° < θ < 90°, while the *cis* S_1_min has 10° < θ < 30°. The error bars were calculated according to the eq 5.

The potential energy surfaces in Figure 5 suggested no preference for either photoisomerization or cyclization pathways. Hence, one could expect the wavepacket to bifurcate equally into those pathways. Unequal bifurcation into the pathways was already demonstrated in Figure 6. To further investigate this bifurcation, we track the evolution of the dihedral angle θ along the carbon backbone C_1_–C_0_=C_0′_–C_1′_, see Figure 7. Compared with the distance *R*, which defines the distance between the benzene rings, the dihedral θ is significantly floppier. It is apparent that the double bond can change configuration even without significant motion of the heavy benzene rings. The analysis in Figure 7 indicates that the double bond first twists toward 90° for all methods before settling into the *cis*, *trans*, or DHP configurations.

**Figure 8:**
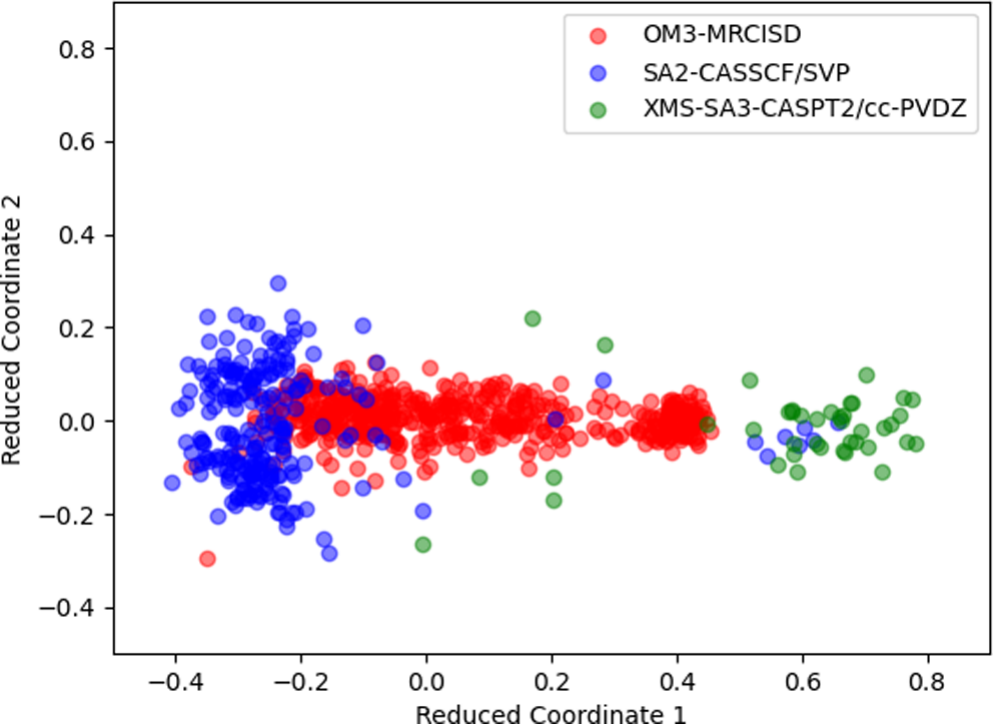
Analysis of hopping geometries using MDS. The axes depict the most significant reduced coordinates identified by the MDS algorithm for a combined set of geometries across all of the methods. The resulting plot categorizes the geometries by methods, each represented by a unique color.

Finally, we applied the MDS algorithm to visualize the hopping geometries for simulations with different potential energy surfaces (see the Methods Section for details). We have considered 486 OM3-MRCISD hopping geometries, 231 SA2-CASSCF/SVP hopping geometries, and 41 XMS-SA3-CASPT2/cc-pVDZ hopping geometries. The first two reduced coordinates plotted in Figure 8 cover the majority of variance in the data while preserving distances between the geometries as much as possible. MDS was applied to hopping geometries from all methods collectively, ensuring a unified coordinate system for plotting. Analysis of the plot reveals a distinctive pattern: the transition geometries from the XMS-SA3-CASPT2 methods predominantly appear on the right side, while those from other methods are mainly situated on the left. This observation aligns with the fact that XMS-SA3-CASPT2 trajectories rarely yield a significant percentage of the *trans*-stilbene isomer. Consequently, we infer that in these reduced coordinates, the right side of the plot corresponds to transition geometries leading to DHP, whereas the left side predominantly represents the *trans* isomer.

### 3.3 Simulated Time-Resolved Photoelectron Spectra

Arguably, the most detailed insight into stilbene photodynamics is provided by time-resolved photoelectron spectroscopy, which was recently conducted by three research teams. All three teams initiated the reaction with a 266 nm laser field, and the evolving system was then probed by a 4.65 eV laser (Stolow group^21^), 9 eV (Wörner group^36^), and 21 eV (Suzuki group^23^). The pump–probe signal stems exclusively from the excited state in the first case, while the interpretation of the latter two experiments requires the consideration of reactants, intermediates, and products in both the ground and excited states. In our analysis, we will focus on the regions 5–6.5 eV (Wörner data) and 3.0–6.8 eV (Suzuki data). In both cases, these signals are directly related to stilbene in the excited state. Further, we analyze the regions 7.0–7.3 and 7.5–7.8 eV. Here, the signal should be indicative of the DHP formation and, as we show, also the hot *trans* isomer.

**Figure 9:**
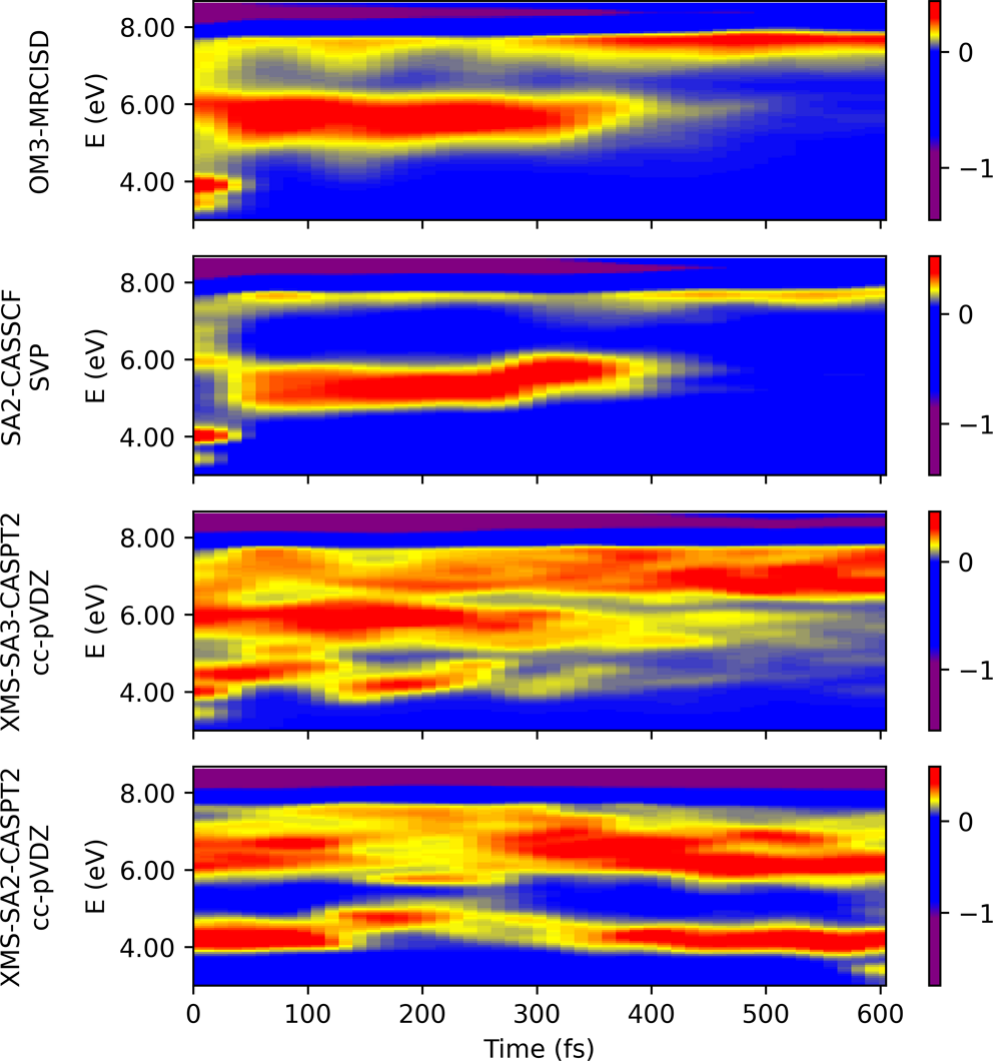
Simulated photoelectron spectra for surface hopping dynamics with various electronic structure methods; the intensities were included via Dyson norms. The pump–probe signal was calculated by subtracting the ground-state signal at *t* = 0.

The calculated photoelectron spectra for each electronic structure method are presented in Figure 9. As for the electronic populations, the calculated spectra exhibit discernible disparities across the various electronic structure methods utilized. The OM3-MRCISD, SA2-CASSCF, and XMS-SA3-CASPT2 dynamical calculations feature a brief 4 eV component, accompanied by a decay of the excited-state component (6–6.5 eV) in about 400 fs, while the XMS-SA2-CASPT2 calculations feature the 4 eV component throughout the simulation time. These results are consistent with the previous findings regarding the electronic populations.

**Figure 10:**
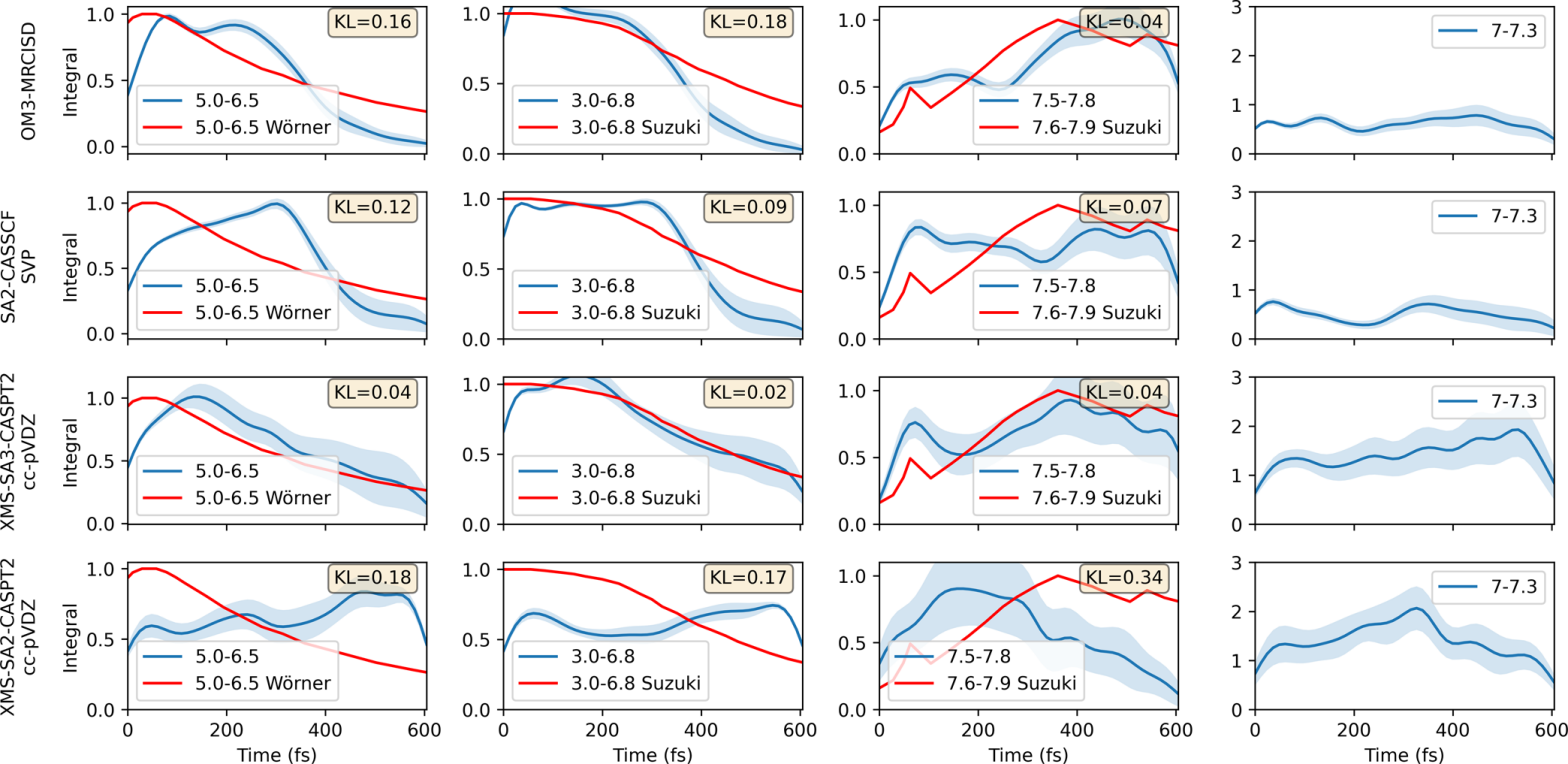
Integrated time-resolved photoelectron spectra. The first column presents the integrals of the calculated spectra from Figure 9 in the range 5 to 6.5 eV (blue line) along with the experimental spectrum (red line).^36^ The second column displays the integrated calculated spectrum (blue line) from 3 to 6.8 eV along with the corresponding experimental spectrum (red line). The third column is analogous, with the energy range from 7.5 to 7.9 eV.^23^ The fourth column corresponds to the 7.0 to 7.3 eV that should be most sensitive to the DHP formation.^23^ All of the integrals were convoluted with Gaussian function, with a standard deviation of 30 fs. The comparisons with the experimental data are normalized to the maximum value and aligned using the least-squares method. The error bars are calculated using the bootstrapping method using 10,000 samples, and for quantitative comparison with the experiments, we used the Kullback–Leibler (KL) divergence, where the lower value means better agreement with the experiment.

The calculated spectra exhibit trends consistent with the experiments for all methods except for XMS-SA2-CASPT2. The agreement between different approaches and the experiments^23,36^ can be best compared in the integrated signals, see Figure 10. The integration range for the comparison with the experiment performed by Suzuki was shifted by 0.1 eV due to the proximity to the negative part of the spectrum. The data in the first two columns in Figure 10 indicate the excited-state lifetime of stilbene: once the system quenches to the ground state, the electrons with a low binding energy are no longer populated. The XMS-SA3-CASPT2 method shows the best reproduction of the data. Yet the MRCISD-OM3 and SA2-CASSCF methods display the same features. The difference between the methods seems to be related to the time evolution of the internal conversion process rather than to the formation of the different products.

The region of 7.5–8.0 eV was assigned by Suzuki et al. as the ionization from the *cis*-stilbene in the S_1_ state to a higher cation state. This is indeed what we observe in our simulations in the early stages. However, the signal is persistent even at the times when all of the excited-state population is dumped to the ground state. We then observe from our simulations that the 7.5–8.0 eV pump–probe signal corresponds to the hot *trans*-stilbene formed once the excited state depopulates or to the DHP. Note that *trans*-stilbene has ionization energy somewhat lower than *cis*-stilbene.^23^

The experiments by Suzuki with the highest photon energy bear in principle information on the emergence of the ground-state products, yet the interpretation is complicated, as the fraction of excited molecules is unknown. The signal corresponding to the formation of DHP is estimated to peak at 7.1 eV.^23^ Therefore, we also integrated the spectra into the 7.0–7.3 eV range. The time evolution of this signal is similar for different methods, yet the signal on an absolute scale is larger for the XMS-SA3-CASPT2 method, in agreement with the higher DHP formation. However, the signal is still present for the OM3-MRCISD and SA2-CASSCF methods, even though no DHP is formed.

The calculated photoelectron spectra reflect the photoproduct population during the simulation. While the OM3-MRCISD method leads to a significant formation of the *trans*-stilbene, it shows little DHP-related signal. On the other hand, the XMS-SA3-CASPT2 method predicts too much DHP, and it fails to describe the formation of *trans*-stilbene. However, it is very difficult to judge from the experimental spectra how much DHP was formed during the photochemical reaction. The signals assigned to DHP can also stem from the *trans*-stilbene formation.

### 3.4 Nonadiabatic Simulations with a Bias

The simulations of *cis*-stilbene photoisomerization have illustrated the critical impact of the electronic structure theory method on observable quantities. The previous analysis revealed that treating the methods separately brings seeming reliability compared to the experiments, but only when taken as an ensemble was the discrepancy displayed. However, these simulations are computationally expensive, limiting our capacity to conduct a wide array of simulations with various electronic structure methods. Nonetheless, conducting a basic sensitivity analysis remains valuable.

One approach to navigate this challenge is to carry out the most efficient simulations, exemplified here by employing the semiempirical method with the OM3 Hamiltonian, supplemented by an additional potential to modify the potential energy surface. If then a small perturbation of the potential leads to a major change in the outcome, we are at least aware of the sensitivity issue. We have introduced a potential in the form of a simple harmonic oscillator, defined by the equation
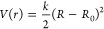
7In this expression, *R* represents the distance between carbons 2 and 2′, as depicted in Figure 2, and *k* signifies the force constant measured in eV Å^–2^. The constant *R*_0_ is set to 1.5 Å to stimulate the DHP molecule formation: a higher force constant implies a stronger tendency toward closing the cycle.

**Figure 11:**
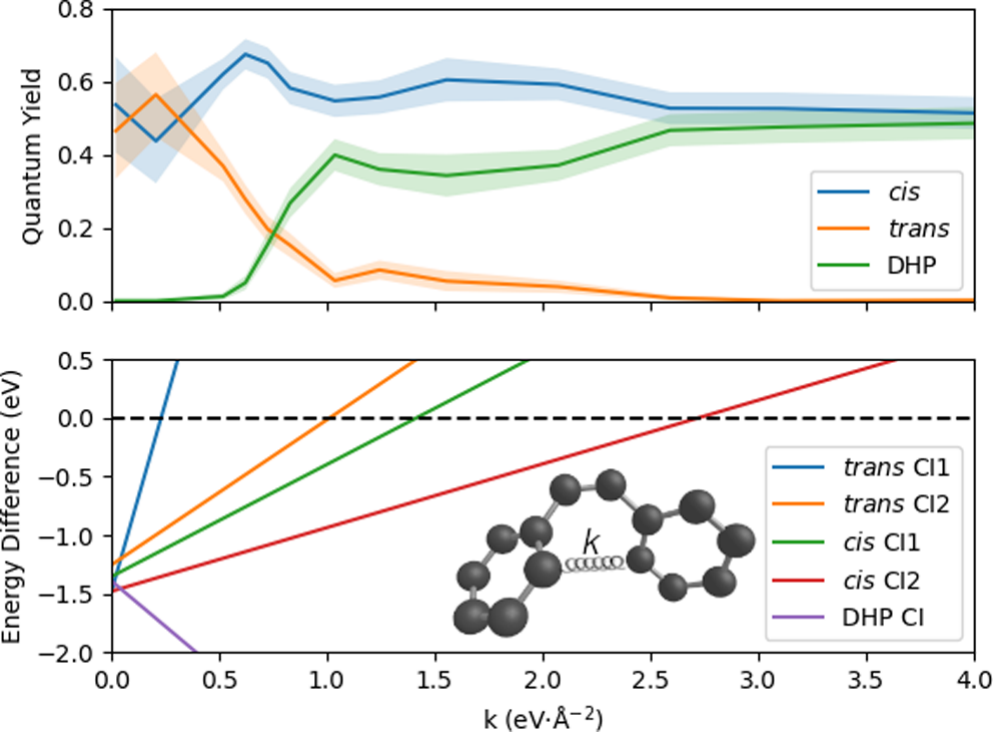
Quantum yields of *cis*-stilbene photoproducts as a function of the applied force constant. The upper panel shows the product ratio on the force constant *k* of the harmonic oscillator, as referenced in eq 7. The bottom panel shows the energy difference between the Franck–Condon point (dashed line) and several conical intersections, also varying with different values of *k*.

The upper panel of Figure 11 demonstrates how the quantum yield of DHP varies with force constant *k*. For small force constants, the DHP quantum yield is zero, consistent with the OM3-MRCISD simulations failing to capture its formation. However, there is a sharp increase in the DHP quantum yield around *k* = 0.83 eV Å^–2^, indicating that the potential energy surfaces are biased enough to start pulling the molecule toward the DHP conical intersection. The lower panel of Figure 11 further illustrates the energetics of different stationary points on the potential energy surface when the bias is applied, with the excited-state energy at the Franck–Condon (FC) point serving as a reference. Conical intersections positioned above the FC energy (marked by the dashed line) are energetically inaccessible. Following this logic, the population of *trans*-stilbene should diminish for *k* values exceeding roughly 1 eV Å^–2^, consistent with the observed quantum yields. Notably, the DHP quantum yield peaks locally and never reaches unity, indicating that the *cis* CI2 conical intersection remains energetically viable within the range of *k* values that we have explored. The fate of the reaction is largely determined in the initial phase during the bifurcation into the cyclization or photoisomerization pathways.

**Figure 12:**
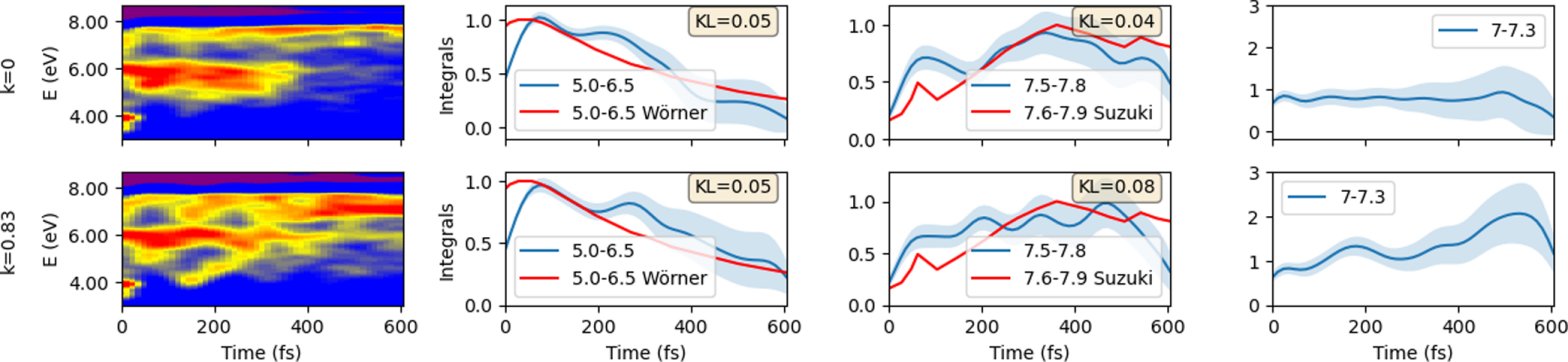
Time-resolved photoelectron signals for biased simulations. The left panel shows the generated spectra for the biased and unbiased simulations at the OM3-MRCISD level, while the right panel presents the corresponding integrals across different spectral ranges. The comparisons with the experimental data are normalized to the maximum value and aligned using the least-squares method. The error bars are calculated using the bootstrapping method using 10,000 samples, and for quantitative comparison with the experiments, we used the Kullback–Leibler (KL) divergence, where the lower value means better agreement with the experiment.

The biased simulations are further scrutinized in Figure 12, where we present the calculated photoelectron spectrum for the biased and unbiased cases. The upper panel illustrates the unbiased simulations, while the lower panel depicts simulations with a notably large value of *k* = 0.83 eV Å^–2^, corresponding to a DHP quantum yield of 0.26. In the unbiased simulation, the entire wavepacket swiftly (within 200 fs) converges toward structures with larger C–C distances before gradually dispersing. Conversely, the bias compels molecules to close the cycle, yet some molecules still prefer photoisomerization. This difference is mirrored in the calculated spectra, with a stronger signal in the 7–8 eV region where the DHP signal increase becomes apparent in the biased simulation. While the difference is visually pronounced in the 2D spectrum, the integrated signals between 5–6.5 and 7.5–7.8 eV appear similar to the experiment, demonstrating the integrated signals as quite forgiving quantities. A discernible difference can be seen for the integration range of 7–7.3 eV, reflecting the different quantum yields of the product.

Another significant disparity between the simulations is observed in the excited-state lifetime. The characteristic reaction time spans about 300 fs for the unbiased simulation, whereas it approaches 400 fs for the biased simulation with *k* = 0.83 eV Å^–2^. The lifetimes calculated with the unbiased simulations with all electronic structure methods show too fast decay (if there is a decay at all); the present value for biased simulations is more consistent with the experimental observations.

## 4 Conclusions and Discussion

We have modeled the photodynamics of *cis*-stilbene with an ensemble of nonadiabatic simulations based on different electronic structure methods, highlighting the difference compared with a separate treatment based on a single electronic structure. First, we show that inspecting the potential energy surfaces could hardly reveal discrepancies in the dynamics. While we could single out XMS-SA2-CASPT2 as obviously inconsistent with the rest, the remaining methods appeared as a reasonable choice for dynamics, depending on the computational resources available. Intriguingly, the photoelectron spectra calculated from the nonadiabatic dynamics simulations appeared comparable and difficult to assess when only a single method was considered. However, the perspective of an ensemble of methods displays a large disparity hidden under the reasonably looking simulated photoelectron spectra.

The differences were substantial: while for some approaches (XMS-SA2-CASPT2), negligible population transfer was observed within the first 500 fs, other methods agreed on the ultrafast dynamics being essentially complete on that time scale. The quantum yields within the ensemble of approaches were also very different. While the XMS-SA3-CASPT2/cc-pVDZ method showed negligible *trans* isomer production and dominant DHP formation, the other methods indicated much lower DHP yields, a result more consistent with experimental data in the liquid phase. Qualitatively distinct dynamics were achieved only by changing the number of states we average over in the multireference methods, although stilbene is a molecule where state-averaging dependence would not be suspected.

The results show two critical points in stilbene’s reaction mechanism. First, the initial bifurcation of the wavepacket into cyclization or photoisomerization pathways. Second, reaching the conical intersection to form DHP in the cyclization pathway. We conclude that none of the simulations are completely satisfactory. Most of the methods contradict the experimental photoelectron spectra, indicating the presence of DHP in the early times of the dynamics, while the XMS-SA3-CASPT2 method overestimates the DHP formation. At the same time, all of the electronic structure methods underestimate the excited-state lifetimes compared to the most recent experiments yet agree with older measurements.

Suzuki et al. argued that photoexcited *cis*-stilbene favors ring closure over photoisomerization in the gas phase. This would indicate rather strong environmental effects; the ring closure is important in nonpolar solvents yet by far not dominating over the photoisomerization. Our results indicate that the time-resolved data can be equally interpreted with a much higher *trans*-stilbene quantum yield. Thus, we conclude that none of the simulations can fully explain the photoelectron experimental spectra. What is the correct answer, then? We believe that experiments focusing on geometrical features, such as ultrafast electron diffraction,^57^ rather than electronic quantities like photoelectron spectra might cut the Gordian knot.

Our study suggests that we should be more careful with the evaluation of nonadiabatic simulations. The usual strategy rests on mapping the potential energy surface with an expensive electronic structure method that is then used to validate an approach more amiable to nonadiabatic simulations with thousands of electronic structure evaluations. However, such an approach does not seem to be satisfactory. The approach presented here with an ensemble of electronic structure methods is more robust. Combined with machine learning approaches, an ensemble of methods can deliver more accurate results as well as the estimate of the systematic uncertainty, as demonstrated, e.g., in a recent work on the use of an ensemble of functionals.^42^ Performing multiple simulations with different electronic structure methods is, however, impractical. Furthermore, the limited availability of electronic structure methods for global potential energy surfaces brings additional problems.

The dynamics with biases seem to be an alternative. Using a thrifty method such as the OM3-MRCISD approach with multiple biases can provide a hint at the potential problems with our calculations. Therefore, we propose a sensitivity analysis approach based on adding a small perturbing potential into the system. If a strong variation of the reaction outcome with a small change in the additional potential is observed, then the interpretation of the experimental data should be performed with specific care. Our simulations with a bias potential were able to bridge methods with negligible and dominant DHP formation, helping us understand how much energy is necessary to transfer between these two frameworks. This strategy could certainly be refined to incorporate more nuanced considerations. Nongeometrical constraints, such as energy difference constraints or penalties explicitly targeting certain conical intersections, could be envisaged.

Incorporating perturbing potentials is a common tactic in computational statistical mechanics, akin to utilizing metadynamics.^58^ Similarly, empirical potentials have been employed to enhance photodynamical simulations by compensating for missing correlation energy,^59,60^ aligning with the principles of Δ machine learning. However, in this instance, our aim is solely to augment the system with an additional potential to evaluate the reliability of our computed results. One can think of changing the paradigm of these approaches and employing them in the sensitivity analysis business. It would be interesting to apply a similar procedure to other molecules, such as tetraphenylethene and its derivatives where previous studies have indicated difficulties with the electronic structure and nonadiabatic dynamics.^61,62^

Armed with experimental data, we could contemplate designing additional potentials to optimize the agreement with experimental observations. In this scenario, quantum yields, excited-state lifetimes, and time-resolved photoelectron spectra can be computed and compared with experimental data for any trajectory—whether unbiased or biased. The forces can then be adjusted to achieve the closest match with the experimental findings. In our current study, we have utilized quantum yields as inputs, and the results exhibit a notably improved agreement with experimental data. This approach bears a resemblance to the construction of optimal pulses, and the technology of optimal control could be directly applied. A similar strategy is used in other fields where the calculations are performed with constraints provided by the experiment. For example, the structure of complicated biomolecules can be conveniently estimated within the quantum refinement technique.^63^

We also note that the photodynamics community should be, in general, more careful with the experimental interpretation. All available experimental data should be considered simultaneously. In biology, it is common not to stop whenever the hypothesis confirms an experimental observation. An alternative hypothesis is typically suggested, and the outcome is compared. We advocate for adopting this attitude, even in the field of photodynamics.

Johannes Kepler, in his essay on the six-cornered snowflake, expressed the hope that truth could be uncovered by comparing many mistakes. We suggest that this might also be the most productive approach for the field of photodynamics.

## Supporting Information Available

Energies from the mapping of PESs, Cartesian coordinates of all optimized geometries, analysis of the simulations using the SVP basis set, additional plots for the biased simulations, a comparison of photoelectron spectra with ionization energies from SA2-CASSCF/SVP, OM3-MRCISD theories, a comparison of OM3-MRCISD simulations using FSSH and LZSH algorithms, a comparison of simulations with different number of trajectories, and a comparison of simulations with different calculation algorithm for the electronic-state population and comparison of simulations with different initial conditions (PDF)
