# Supplementary material for: Sensitivity Analysis in Photodynamics: How Does the Electronic Structure Control cis-Stilbene Photodynamics?
Source: J Chem Theory Comput. 2024 Dec 12;20(24):10972–85. doi: 10.1021/acs.jctc.4c01008 (PMC11672677; doi:10.1021/acs.jctc.4c01008)
Supplement: Supplementary file 1 — ct4c01008_si_001.pdf [file ct4c01008_si_001.pdf]

# Supporting information

# Sensitivity analysis in photodynamics: How the electronic structure controls cis-stilbene photodynamics?

Tomaš Jíra, Jiří Janoš and Petr Slavíček*
University of Chemistry and Technology, 166 28 Prague 6, Czech Republic
* Corresponding author: Petr.Slavicek@vscht.cz

## I. Energies from the mapping of PESs

**Table S1.** OM3(12,17) and all the CASPT2 methods are XMS-CASPT2(2,2). *cis* $S_1$ denotes the $S_1$ energy at the $S_0$ minimum geometry.

| **structures** | **E / eV** | | | | | |
|---|---|---|---|---|---|---|
| | **OM3** | **SA2-CASSCF svp** | **SA2-CASPT2 svp** | **SA3-CASPT2 svp** | **SA2-CASPT2 cc-pvdz** | **SA3-CASPT2 cc-pvdz** |
| *cis* | 0.00 | 0.00 | 0.00 | 0.00 | 0.00 | 0.00 |
| *trans* | -0.05 | -0.20 | -0.06 | -0.06 | -0.08 | -0.09 |
| DHP | 1.96 | 1.82 | 1.55 | 1.45 | 1.61 | 1.51 |
| *cis* $S_1$ | 5.25 | 5.94 | 4.59 | 4.89 | 4.40 | 4.70 |
| *trans* $S_1$ | 5.17 | 5.34 | 4.23 | 4.52 | 4.08 | 4.38 |
| DHP $S_1$ | 4.74 | 6.01 | 4.02 | 4.16 | 3.92 | 4.11 |
| twist $S_1$ | 3.59 | 4.22 | 4.06 | 3.76 | 3.91 | 3.59 |
| *cis* $S_1$min | - | 4.64 | 3.28 | 3.61 | 3.24 | 3.51 |
| *trans* $S_1$min | - | 4.81 | 3.85 | 4.14 | 3.74 | 4.05 |
| DHP $S_1$min | 3.94 | 4.34 | 3.31 | 3.17 | 3.18 | 3.11 |
| $CI_{OBF1}$ | 3.76 | 4.38 | 4.08 | 3.85 | 3.96 | 3.69 |
| $CI_{OBF2}$ | 3.89 | 4.64 | 4.27 | 4.08 | 4.16 | 3.93 |
| $CI_{HT1}$ | 3.76 | 4.59 | 4.00 | 3.76 | 3.84 | 3.59 |
| $CI_{HT2}$ | 3.99 | 5.07 | 4.29 | 4.06 | 4.18 | 3.95 |
| $CI_{DHP}$ | 3.85 | 4.34 | 4.03 | 3.62 | 3.97 | 3.52 |

## II. Cartesian coordinates of all optimized geometries

The Cartesian coordinates are presented in the atomic units.

## II.A SA2-CASSCF(2,2)/SVP

***trans* $S_0$min**

$E_{\text{SA2-CASSCF(2,2)/SVP}}$ = -536.74765388 a.u.

| | | | |
|---|---|---|---|
| C | 1.974180 | -0.025211 | 0.066715 |
| C | -1.926080 | -0.051631 | -0.030191 |
| C | 0.544702 | 0.017890 | 0.436109 |
| C | -0.495486 | -0.095373 | -0.394172 |
| C | 2.924290 | -0.210232 | 1.073660 |
| C | -2.873240 | -0.436578 | -0.981811 |
| C | 2.431940 | 0.116877 | -1.247380 |
| C | -2.389040 | 0.362543 | 1.223390 |
| C | 4.279340 | -0.271284 | 0.781533 |
| C | -4.230280 | -0.424707 | -0.692417 |
| C | 3.784290 | 0.056706 | -1.539860 |
| C | -3.743010 | 0.374799 | 1.513140 |
| C | 4.715780 | -0.139435 | -0.527494 |
| C | -4.671680 | -0.020250 | 0.557597 |
| H | 0.360009 | 0.128738 | 1.497360 |
| H | -0.307607 | -0.260573 | -1.447810 |
| H | 2.596420 | -0.314259 | 2.100210 |
| H | -2.541630 | -0.754488 | -1.962140 |
| H | 1.729600 | 0.291824 | -2.050110 |
| H | -1.689640 | 0.691387 | 1.978890 |
| H | 4.994120 | -0.421118 | 1.580340 |
| H | -4.942480 | -0.731765 | -1.447360 |
| H | 4.114770 | 0.171670 | -2.564110 |
| H | -4.077150 | 0.701336 | 2.489470 |
| H | 5.772060 | -0.181703 | -0.758733 |
| H | -5.729280 | -0.006332 | 0.786215 |

***cis* $S_0$min**

$E_{\text{SA2-CASSCF(2,2)/SVP}}$ = -536.74036036 a.u.

| | | | |
|---|---|---|---|
| C | -4.779500 | 0.082052 | 0.415337 |
| C | -4.269420 | -0.580893 | -0.691334 |
| C | -2.901090 | -0.600627 | -0.919934 |
| C | -2.015590 | 0.024050 | -0.039565 |
| C | -2.543160 | 0.698620 | 1.062430 |
| C | -3.911230 | 0.725817 | 1.287210 |
| C | -0.561027 | 0.005518 | -0.341904 |
| C | 0.480677 | -0.153957 | 0.475886 |
| C | 0.514044 | -0.460372 | 1.929090 |
| C | 1.471550 | 0.162411 | 2.732240 |
| C | 1.558310 | -0.116684 | 4.088480 |
| C | 0.698325 | -1.040540 | 4.664080 |

| | | | |
|---|---|---|---|
| C | -0.244202 | -1.683370 | 3.872280 |
| C | -0.335730 | -1.397740 | 2.518470 |
| H | 1.460830 | -0.056936 | 0.020783 |
| H | -0.323637 | 0.155646 | -1.389960 |
| H | 2.153170 | 0.878148 | 2.289700 |
| H | -2.512530 | -1.112730 | -1.791430 |
| H | -1.068060 | -1.911750 | 1.912500 |
| H | -1.879250 | 1.210790 | 1.744210 |
| H | 2.302650 | 0.384300 | 4.693840 |
| H | -4.937060 | -1.079360 | -1.382190 |
| H | -0.909651 | -2.416360 | 4.309820 |
| H | -4.301250 | 1.257480 | 2.145560 |
| H | 0.767259 | -1.265580 | 5.720420 |
| H | -5.846780 | 0.105096 | 0.593278 |

**DHP $S_0$min**

$E_{\text{SA2-CASSCF(2,2)/SVP}}$ = -536.67358784 a.u.

| | | | |
|---|---|---|---|
| C | 1.427740 | -0.865056 | -0.022653 |
| C | -1.431290 | -0.892873 | -0.066421 |
| C | 0.732945 | -2.013300 | -0.026513 |
| C | -0.712937 | -2.023220 | -0.153297 |
| C | 2.883630 | -0.842211 | 0.108671 |
| C | -2.887510 | -0.889832 | -0.196346 |
| C | 0.709258 | 0.458830 | -0.248656 |
| C | -0.740059 | 0.423451 | 0.264261 |
| C | 3.539750 | 0.292759 | 0.355599 |
| C | -3.567150 | 0.247368 | -0.352855 |
| C | 1.500600 | 1.626080 | 0.305023 |
| C | -1.555940 | 1.614330 | -0.195496 |
| C | 2.804480 | 1.551510 | 0.539718 |
| C | -2.858200 | 1.531690 | -0.436212 |
| H | 1.254220 | -2.959700 | 0.048614 |
| H | -1.214610 | -2.971240 | -0.303674 |
| H | 3.413920 | -1.783940 | 0.041069 |
| H | -3.398160 | -1.844720 | -0.203729 |
| H | 0.663829 | 0.598932 | -1.342630 |
| H | -0.696009 | 0.477370 | 1.365940 |
| H | 4.615690 | 0.293548 | 0.471725 |
| H | -4.643010 | 0.235183 | -0.468896 |
| H | 0.985636 | 2.564120 | 0.465621 |
| H | -1.060640 | 2.572510 | -0.281065 |
| H | 3.346480 | 2.419250 | 0.894048 |
| H | -3.418330 | 2.413390 | -0.721141 |

***twist* $S_1$min**

$E_{\text{SA2-CASSCF(2,2)/SVP}}$ = -536.58515977 a.u.

| | | | |
|---|---|---|---|
| C | 1.810590 | -0.269987 | 0.225824 |
| C | -1.745640 | -0.403632 | -0.138322 |
| C | 0.563475 | -0.885254 | 0.523622 |
| C | -0.511908 | -1.116200 | -0.384665 |

| | | | |
|---|---|---|---|
| C | 2.780330 | -0.102004 | 1.239310 |
| C | -2.989120 | -0.933811 | -0.564655 |
| C | 2.076660 | 0.196929 | -1.077640 |
| C | -1.795450 | 0.856333 | 0.498778 |
| C | 3.973300 | 0.524878 | 0.962010 |
| C | -4.170290 | -0.252199 | -0.371586 |
| C | 3.278800 | 0.819304 | -1.353580 |
| C | -2.995540 | 1.528440 | 0.691531 |
| C | 4.213620 | 0.984349 | -0.335628 |
| C | -4.197560 | 0.991889 | 0.261954 |
| H | 0.498129 | -1.196260 | 1.581780 |
| H | -0.646615 | -2.200410 | -0.472080 |
| H | 2.571820 | -0.466495 | 2.236700 |
| H | -3.001470 | -1.895430 | -1.062530 |
| H | 1.309370 | 0.042963 | -1.822690 |
| H | -0.878081 | 1.335550 | 0.823563 |
| H | 4.716080 | 0.663555 | 1.734750 |
| H | -5.095910 | -0.697129 | -0.718300 |
| H | 3.492960 | 1.179410 | -2.350070 |
| H | -2.982510 | 2.493650 | 1.184740 |
| H | 5.150450 | 1.481550 | -0.552867 |
| H | -5.129870 | 1.520870 | 0.408113 |

***trans*** $\mathbf{S_1}$**min**

$E_{\text{SA2-CASSCF(2,2)/SVP}}$ = -536.56377571 a.u.

| | | | |
|---|---|---|---|
| C | 1.922880 | -0.087470 | 0.114677 |
| C | -1.876370 | -0.111198 | -0.071120 |
| C | 0.562616 | -0.108497 | 0.475524 |
| C | -0.516011 | -0.204765 | -0.419921 |
| C | 2.932940 | -0.040427 | 1.129400 |
| C | -2.886410 | -0.280469 | -1.072760 |
| C | 2.374190 | -0.106122 | -1.247400 |
| C | -2.327690 | 0.160551 | 1.263690 |
| C | 4.264280 | -0.020954 | 0.811594 |
| C | -4.217740 | -0.193737 | -0.766399 |
| C | 3.720550 | -0.084528 | -1.543020 |
| C | -3.674060 | 0.244684 | 1.547920 |
| C | 4.677930 | -0.044320 | -0.531934 |
| C | -4.631420 | 0.068744 | 0.551440 |
| H | 0.351667 | -0.059989 | 1.533760 |
| H | -0.305068 | -0.379722 | -1.464710 |
| H | 2.625190 | -0.025244 | 2.166810 |
| H | -2.578620 | -0.485540 | -2.089800 |
| H | 1.659350 | -0.126386 | -2.054530 |
| H | -1.612840 | 0.312749 | 2.056610 |
| H | 5.004560 | 0.012011 | 1.600280 |
| H | -4.958020 | -0.329258 | -1.544060 |
| H | 4.036850 | -0.096724 | -2.578180 |
| H | -3.990370 | 0.452492 | 2.562080 |
| H | 5.730950 | -0.030641 | -0.777546 |
| H | -5.684450 | 0.133591 | 0.788648 |

***cis* $\mathbf{S_1}$min**

$E_{\text{SA2-CASSCF(2,2)/SVP}}$ = -536.57002618 a.u.

| | | | |
|---|---|---|---|
| C | -4.430390 | 0.043604 | 0.522615 |
| C | -4.056880 | -0.628227 | -0.675845 |
| C | -2.746020 | -0.767933 | -1.013970 |
| C | -1.704540 | -0.282902 | -0.161359 |
| C | -2.101590 | 0.462213 | 1.011380 |
| C | -3.468920 | 0.597289 | 1.322570 |
| C | -0.353200 | -0.567158 | -0.424456 |
| C | 0.674626 | -0.429084 | 0.519272 |
| C | 0.479515 | -0.502670 | 1.909510 |
| C | 1.462040 | -0.009769 | 2.825200 |
| C | 1.207570 | 0.064605 | 4.160260 |
| C | -0.035261 | -0.385743 | 4.689390 |
| C | -0.968242 | -0.943606 | 3.859150 |
| C | -0.745064 | -1.026490 | 2.470530 |
| H | 1.688700 | -0.288066 | 0.161016 |
| H | -0.092378 | -0.881716 | -1.429130 |
| H | 2.403780 | 0.348285 | 2.429000 |
| H | -2.469760 | -1.292630 | -1.919630 |
| H | -1.336210 | -1.713030 | 1.888320 |
| H | -1.394750 | 1.132840 | 1.470060 |
| H | 1.954060 | 0.470753 | 4.830300 |
| H | -4.826760 | -1.031280 | -1.320830 |
| H | -1.884000 | -1.352710 | 4.266350 |
| H | -3.751550 | 1.170480 | 2.196170 |
| H | -0.217691 | -0.322563 | 5.753990 |
| H | -5.477780 | 0.149341 | 0.772138 |

***DHP* $\mathbf{S_1}$min**

$E_{\text{SA2-CASSCF(2,2)/SVP}}$ = -536.58101684 a.u.

| | | | |
|---|---|---|---|
| C | 1.427340 | -0.880673 | 0.021720 |
| C | -1.482220 | -0.892654 | -0.019812 |
| C | 0.669064 | -2.070400 | 0.092622 |
| C | -0.715712 | -2.051380 | -0.096618 |
| C | 2.764600 | -0.812803 | 0.503804 |
| C | -2.779490 | -0.794151 | -0.594282 |
| C | 0.843170 | 0.353259 | -0.505338 |
| C | -0.825901 | 0.317306 | 0.522752 |
| C | 3.398520 | 0.392545 | 0.613211 |
| C | -3.448700 | 0.384450 | -0.690540 |
| C | 1.592440 | 1.566590 | -0.404494 |
| C | -1.585830 | 1.552980 | 0.404712 |
| C | 2.809870 | 1.621590 | 0.198884 |
| C | -2.814360 | 1.558980 | -0.164036 |
| H | 1.179720 | -3.006760 | 0.269270 |
| H | -1.220840 | -2.988120 | -0.314187 |
| H | 3.248450 | -1.716670 | 0.847719 |
| H | -3.223280 | -1.702330 | -0.986963 |
| H | 0.266408 | 0.249414 | -1.416330 |
| H | -0.307595 | 0.175902 | 1.467790 |

| | | | |
|---|---|---|---|
| H | 4.393550 | 0.415164 | 1.042280 |
| H | -4.435210 | 0.445310 | -1.125710 |
| H | 1.152070 | 2.464410 | -0.822395 |
| H | -1.179860 | 2.458370 | 0.835641 |
| H | 3.348450 | 2.550610 | 0.317844 |
| H | -3.362740 | 2.494130 | -0.187112 |

## OBF1

$E_{\text{SA2-CASSCF(2,2)/SVP}} = -536.57936369$ a.u.

| | | | |
|---|---|---|---|
| C | 0.387734 | -0.656381 | 0.543234 |
| C | 0.120070 | -0.210353 | 1.911680 |
| C | -0.723629 | 0.885118 | 2.160950 |
| C | -1.021080 | 1.282700 | 3.456160 |
| C | -0.493589 | 0.609621 | 4.548120 |
| C | 0.338177 | -0.480808 | 4.316510 |
| C | 0.637482 | -0.888133 | 3.028990 |
| C | 0.777537 | 0.146922 | -0.519645 |
| H | 1.528330 | -0.865916 | 0.510096 |
| H | 1.531590 | 0.943661 | -0.396002 |
| C | 0.388290 | -0.019259 | -1.912640 |
| C | 0.960853 | 0.798137 | -2.896060 |
| C | 0.556391 | 0.693604 | -4.214650 |
| C | -0.419243 | -0.236984 | -4.557040 |
| C | -0.990729 | -1.061290 | -3.591760 |
| C | -0.587012 | -0.958729 | -2.273290 |
| H | -1.162210 | 1.420710 | 1.328050 |
| H | -1.675370 | 2.132490 | 3.609790 |
| H | -0.727572 | 0.922128 | 5.557050 |
| H | 0.758292 | -1.025240 | 5.153560 |
| H | 1.273330 | -1.750950 | 2.875600 |
| H | 1.719730 | 1.518650 | -2.618140 |
| H | 0.994824 | 1.327330 | -4.973200 |
| H | -0.734841 | -0.323785 | -5.588850 |
| H | -1.745330 | -1.782460 | -3.874510 |
| H | -0.992495 | -1.589210 | -1.495530 |

## OBF2

$E_{\text{SA2-CASSCF(2,2)/SVP}} = -536.56982990$ a.u.

| | | | |
|---|---|---|---|
| C | 0.015481 | 0.033488 | -0.000640 |
| C | 0.009326 | -0.012937 | 1.446740 |
| C | 1.222020 | -0.082036 | 2.159260 |
| C | 1.238710 | -0.074730 | 3.544050 |
| C | 0.061586 | 0.005829 | 4.276800 |
| C | -1.141490 | 0.082636 | 3.585600 |
| C | -1.174370 | 0.074739 | 2.201770 |
| C | 0.540421 | -0.756012 | -1.005540 |
| H | -0.900760 | -0.552322 | -0.426386 |
| H | 0.866854 | -0.271620 | -1.933860 |
| C | 0.625055 | -2.228270 | -1.126770 |
| C | 1.085260 | -2.771370 | -2.328380 |

| | | | |
|---|---|---|---|
| C | 1.243930 | -4.141520 | -2.469600 |
| C | 0.938958 | -4.977890 | -1.405330 |
| C | 0.479446 | -4.449880 | -0.201127 |
| C | 0.325706 | -3.083760 | -0.057924 |
| H | 2.156270 | -0.131649 | 1.614780 |
| H | 2.190920 | -0.130198 | 4.057970 |
| H | 0.081067 | 0.013775 | 5.358250 |
| H | -2.073890 | 0.151132 | 4.132950 |
| H | -2.126090 | 0.149833 | 1.690820 |
| H | 1.320680 | -2.114260 | -3.156000 |
| H | 1.599830 | -4.554440 | -3.403630 |
| H | 1.060150 | -6.048180 | -1.510430 |
| H | 0.248331 | -5.108180 | 0.625190 |
| H | -0.021496 | -2.670880 | 0.877879 |

**HT1**

$E_{\text{SA2-CASSCF(2,2)/SVP}}$ = -536.57179503 a.u.

| | | | |
|---|---|---|---|
| C | 0.042809 | 0.039892 | 0.020602 |
| C | -0.041998 | -0.079332 | 1.568650 |
| C | 1.097170 | 0.158983 | 2.343040 |
| C | 1.094900 | -0.028953 | 3.723900 |
| C | -0.052954 | -0.461400 | 4.360360 |
| C | -1.211000 | -0.691927 | 3.614190 |
| C | -1.211460 | -0.478056 | 2.251780 |
| C | -0.090681 | 1.438540 | 0.191647 |
| H | -0.909581 | -0.386043 | -0.312167 |
| H | -0.957193 | 1.909870 | 0.688202 |
| C | 0.894227 | 2.442540 | -0.184976 |
| C | 0.675631 | 3.793760 | 0.119217 |
| C | 1.569050 | 4.757550 | -0.307701 |
| C | 2.686860 | 4.370200 | -1.042940 |
| C | 2.916970 | 3.033490 | -1.352560 |
| C | 2.026390 | 2.066730 | -0.919514 |
| H | 2.008120 | 0.459583 | 1.844800 |
| H | 1.997810 | 0.152291 | 4.292740 |
| H | -0.056923 | -0.621653 | 5.430870 |
| H | -2.111890 | -1.033840 | 4.107790 |
| H | -2.117880 | -0.649131 | 1.684730 |
| H | -0.198729 | 4.077640 | 0.691036 |
| H | 1.404820 | 5.800710 | -0.075511 |
| H | 3.389190 | 5.123340 | -1.377310 |
| H | 3.789950 | 2.753420 | -1.926020 |
| H | 2.155460 | 1.013090 | -1.120270 |

**HT2**

$E_{\text{SA2-CASSCF(2,2)/SVP}}$ = -536.55412551 a.u.

| | | | |
|---|---|---|---|
| C | 1.556490 | -0.164629 | 0.198366 |
| C | 0.550369 | -0.126600 | 1.265860 |
| C | -0.284071 | 0.980623 | 1.471190 |
| C | -1.160680 | 1.002300 | 2.538480 |

| | | | |
|---|---|---|---|
| C | -1.209310 | -0.073212 | 3.422140 |
| C | -0.384325 | -1.174630 | 3.237310 |
| C | 0.487100 | -1.199980 | 2.161380 |
| C | 1.932280 | 0.918403 | -0.697327 |
| H | 2.151250 | -1.073490 | 0.270215 |
| H | 1.837910 | 1.925730 | -0.307009 |
| C | 0.948693 | 0.533982 | -1.617110 |
| C | -0.046585 | 1.382290 | -2.244480 |
| C | -1.139920 | 0.841449 | -2.830220 |
| C | -1.381180 | -0.571852 | -2.846060 |
| C | -0.428572 | -1.399830 | -2.341090 |
| C | 0.763179 | -0.883980 | -1.766640 |
| H | -0.241140 | 1.811500 | 0.784297 |
| H | -1.806780 | 1.856230 | 2.689910 |
| H | -1.894100 | -0.048845 | 4.259830 |
| H | -0.423617 | -2.007740 | 3.925680 |
| H | 1.128650 | -2.058580 | 2.009820 |
| H | 0.102086 | 2.454270 | -2.223950 |
| H | -1.867610 | 1.496420 | -3.295200 |
| H | -2.280700 | -0.960364 | -3.302770 |
| H | -0.548844 | -2.474250 | -2.415320 |
| H | 1.618590 | -1.537930 | -1.696840 |

**$CI_{DHP}$**

$E_{\text{SA2-CASSCF(2,2)/SVP}}$ = -536.5808233 a.u.

| | | | |
|---|---|---|---|
| C | 1.469210 | -0.890988 | -0.023070 |
| C | -1.442060 | -0.882857 | -0.066182 |
| C | 0.703936 | -2.046200 | 0.023873 |
| C | -0.680894 | -2.064910 | -0.185625 |
| C | 2.758590 | -0.796931 | 0.570661 |
| C | -2.792530 | -0.810671 | -0.514406 |
| C | 0.805847 | 0.326928 | -0.544215 |
| C | -0.868588 | 0.344258 | 0.487349 |
| C | 3.426990 | 0.379307 | 0.692304 |
| C | -3.426720 | 0.398156 | -0.589778 |
| C | 1.561760 | 1.561960 | -0.401085 |
| C | -1.619940 | 1.552960 | 0.426908 |
| C | 2.790450 | 1.553980 | 0.169719 |
| C | -2.844270 | 1.624400 | -0.165441 |
| H | 1.207060 | -2.985780 | 0.237105 |
| H | -1.188100 | -2.996670 | -0.390869 |
| H | 3.198260 | -1.711660 | 0.953299 |
| H | -3.282890 | -1.707070 | -0.867485 |
| H | 0.284138 | 0.196434 | -1.489020 |
| H | -0.269360 | 0.224586 | 1.381030 |
| H | 4.410600 | 0.438311 | 1.133210 |
| H | -4.428400 | 0.422515 | -1.004270 |
| H | 1.161550 | 2.473540 | -0.823544 |
| H | -1.175720 | 2.439380 | 0.865449 |
| H | 3.339650 | 2.488930 | 0.197869 |
| H | -3.386630 | 2.553010 | -0.264021 |

## II.B XMS-SA2-CASPT2(2,2)/SVP

***trans* S$_0$min**

$E_{\text{XMS-SA2-CASPT2(2,2)/SVP}}$ = -538.556025973 a.u.

| | | | |
|---|---|---|---|
| C | 1.968240 | -0.025297 | 0.066564 |
| C | -1.919950 | -0.048620 | -0.029804 |
| C | 0.553446 | 0.013040 | 0.445012 |
| C | -0.503608 | -0.091663 | -0.401739 |
| C | 2.939250 | -0.229978 | 1.067600 |
| C | -2.888260 | -0.426978 | -0.981704 |
| C | 2.411460 | 0.139141 | -1.263260 |
| C | -2.368490 | 0.359378 | 1.244860 |
| C | 4.299680 | -0.288620 | 0.755013 |
| C | -4.250830 | -0.415735 | -0.672730 |
| C | 3.769390 | 0.071001 | -1.577240 |
| C | -3.728410 | 0.362339 | 1.556770 |
| C | 4.721080 | -0.141108 | -0.570651 |
| C | -4.677380 | -0.022800 | 0.600124 |
| H | 0.360853 | 0.105370 | 1.519340 |
| H | -0.307150 | -0.250659 | -1.467620 |
| H | 2.616640 | -0.348954 | 2.105860 |
| H | -2.562000 | -0.737751 | -1.978290 |
| H | 1.687980 | 0.336924 | -2.056910 |
| H | -1.647860 | 0.689386 | 1.995870 |
| H | 5.033210 | -0.448724 | 1.548460 |
| H | -4.981830 | -0.713385 | -1.427870 |
| H | 4.091400 | 0.198682 | -2.613450 |
| H | -4.053840 | 0.679273 | 2.550450 |
| H | 5.783530 | -0.184533 | -0.819520 |
| H | -5.741360 | -0.010811 | 0.845924 |

***cis* S$_0$min**

$E_{\text{XMS-SA2-CASPT2(2,2)/SVP}}$ = -538.5539166219 a.u.

| | | | |
|---|---|---|---|
| C | -4.778680 | 0.081961 | 0.415591 |
| C | -4.268420 | -0.580985 | -0.690988 |
| C | -2.900090 | -0.599995 | -0.919843 |
| C | -2.014840 | 0.025211 | -0.039743 |
| C | -2.542570 | 0.699870 | 1.062130 |
| C | -3.910570 | 0.726359 | 1.287180 |
| C | -0.560218 | 0.007163 | -0.341828 |
| C | 0.481267 | -0.152051 | 0.476351 |
| C | 0.513988 | -0.460072 | 1.929250 |
| C | 1.470490 | 0.162313 | 2.733760 |
| C | 1.556290 | -0.118163 | 4.089790 |
| C | 0.696256 | -1.042970 | 4.663740 |
| C | -0.245167 | -1.685570 | 3.870470 |
| C | -0.335709 | -1.398580 | 2.516920 |
| H | 1.461560 | -0.054165 | 0.021746 |
| H | -0.322505 | 0.157971 | -1.389720 |
| H | 2.152040 | 0.878949 | 2.292550 |
| H | -2.511410 | -1.112170 | -1.791270 |

| | | | |
|---|---|---|---|
| H | -1.067180 | -1.912380 | 1.909780 |
| H | -1.878790 | 1.212670 | 1.743570 |
| H | 2.300010 | 0.382405 | 4.696210 |
| H | -4.935910 | -1.079920 | -1.381650 |
| H | -0.910320 | -2.419670 | 4.306620 |
| H | -4.300750 | 1.258240 | 2.145340 |
| H | 0.764630 | -1.269280 | 5.719850 |
| H | -5.845980 | 0.104825 | 0.593475 |

**DHP $S_0$min**

$E_{\text{XMS-SA2-CASPT2(2,2)/SVP}}$ = -538.497114948 a.u.

| | | | |
|---|---|---|---|
| C | 1.432680 | -0.868146 | -0.022703 |
| C | -1.441770 | -0.887376 | -0.066358 |
| C | 0.715302 | -2.044170 | -0.035499 |
| C | -0.707877 | -2.050770 | -0.144819 |
| C | 2.868540 | -0.849492 | 0.055375 |
| C | -2.878330 | -0.882526 | -0.132016 |
| C | 0.706684 | 0.443481 | -0.257944 |
| C | -0.733814 | 0.413467 | 0.264513 |
| C | 3.546760 | 0.319211 | 0.268838 |
| C | -3.573460 | 0.289833 | -0.252440 |
| C | 1.477780 | 1.618430 | 0.283561 |
| C | -1.522490 | 1.614130 | -0.187413 |
| C | 2.820140 | 1.558560 | 0.467046 |
| C | -2.865320 | 1.550100 | -0.366095 |
| H | 1.255440 | -2.995180 | 0.012007 |
| H | -1.234400 | -3.003200 | -0.261863 |
| H | 3.406650 | -1.801470 | 0.013902 |
| H | -3.403830 | -1.841940 | -0.156075 |
| H | 0.642581 | 0.565918 | -1.368280 |
| H | -0.671003 | 0.452380 | 1.381050 |
| H | 4.635600 | 0.314419 | 0.358183 |
| H | -4.662960 | 0.276708 | -0.331545 |
| H | 0.947976 | 2.556190 | 0.467074 |
| H | -1.005050 | 2.568200 | -0.310846 |
| H | 3.363240 | 2.442840 | 0.810316 |
| H | -3.422470 | 2.448630 | -0.643375 |

***twist* $S_1$min**

$E_{\text{XMS-SA2-CASPT2(2,2)/SVP}}$ = -538.4049233829 a.u.

| | | | |
|---|---|---|---|
| C | 1.728340 | -0.250954 | 0.216441 |
| C | -1.654170 | -0.556153 | -0.122191 |
| C | 0.547611 | -0.963250 | 0.534705 |
| C | -0.462918 | -1.343910 | -0.413314 |
| C | 2.650660 | 0.137046 | 1.227170 |
| C | -2.963620 | -1.099370 | -0.268718 |
| C | 1.965930 | 0.091607 | -1.143260 |
| C | -1.579250 | 0.802598 | 0.301808 |
| C | 3.791410 | 0.846090 | 0.878980 |
| C | -4.100440 | -0.331253 | -0.045249 |

| | | | |
|---|---|---|---|
| C | 3.108990 | 0.807036 | -1.477730 |
| C | -2.730330 | 1.559440 | 0.548019 |
| C | 4.014210 | 1.181140 | -0.470210 |
| C | -4.001600 | 1.006080 | 0.379770 |
| H | 0.437094 | -1.165510 | 1.627670 |
| H | -0.677407 | -2.422420 | -0.283002 |
| H | 2.445350 | -0.131787 | 2.266760 |
| H | -3.063610 | -2.140010 | -0.591393 |
| H | 1.204470 | -0.246146 | -1.855310 |
| H | -0.602603 | 1.290160 | 0.384540 |
| H | 4.512300 | 1.148940 | 1.640340 |
| H | -5.086070 | -0.783714 | -0.188198 |
| H | 3.307640 | 1.082300 | -2.515060 |
| H | -2.626200 | 2.600090 | 0.868969 |
| H | 4.911140 | 1.745300 | -0.737774 |
| H | -4.899140 | 1.601920 | 0.557928 |

***trans* $S_1$min**

$E_{\text{XMS-SA2-CASPT2(2,2)/SVP}}$ = -538.4123649602 a.u.

| | | | |
|---|---|---|---|
| C | 1.917630 | -0.087385 | 0.114408 |
| C | -1.864470 | -0.229544 | -0.057674 |
| C | 0.579899 | -0.281835 | 0.503179 |
| C | -0.514469 | -0.421214 | -0.403827 |
| C | 2.977920 | -0.163392 | 1.078680 |
| C | -2.911510 | -0.594109 | -0.968498 |
| C | 2.283470 | 0.172348 | -1.254430 |
| C | -2.255730 | 0.313867 | 1.217740 |
| C | 4.303360 | -0.034769 | 0.697842 |
| C | -4.245000 | -0.466829 | -0.615940 |
| C | 3.617900 | 0.308297 | -1.615050 |
| C | -3.598420 | 0.444047 | 1.548970 |
| C | 4.638940 | 0.194845 | -0.654348 |
| C | -4.603750 | 0.046631 | 0.649538 |
| H | 0.378293 | -0.408795 | 1.575980 |
| H | -0.298351 | -0.774469 | -1.421920 |
| H | 2.719210 | -0.345504 | 2.126650 |
| H | -2.633630 | -0.996105 | -1.948070 |
| H | 1.492770 | 0.289088 | -2.001880 |
| H | -1.479090 | 0.650566 | 1.910960 |
| H | 5.094580 | -0.113453 | 1.448080 |
| H | -5.024200 | -0.767604 | -1.320950 |
| H | 3.877020 | 0.506818 | -2.659080 |
| H | -3.877490 | 0.861410 | 2.520890 |
| H | 5.687360 | 0.283984 | -0.951318 |
| H | -5.657710 | 0.134615 | 0.926682 |

***cis* $S_1$min**

$E_{\text{XMS-SA2-CASPT2(2,2)/SVP}}$ = -538.4335138015 a.u.

| | | | |
|---|---|---|---|
| C | -4.547490 | 0.056500 | 0.486632 |
| C | -4.136870 | -0.616611 | -0.694580 |

| | | | |
|---|---|---|---|
| C | -2.793070 | -0.753451 | -0.985743 |
| C | -1.782090 | -0.236616 | -0.117502 |
| C | -2.216730 | 0.437636 | 1.095820 |
| C | -3.589460 | 0.582888 | 1.351590 |
| C | -0.419586 | -0.406403 | -0.427590 |
| C | 0.620567 | -0.167395 | 0.501067 |
| C | 0.481274 | -0.370986 | 1.887190 |
| C | 1.449720 | 0.158647 | 2.792030 |
| C | 1.325720 | -0.007333 | 4.158100 |
| C | 0.215987 | -0.714982 | 4.692150 |
| C | -0.750994 | -1.244040 | 3.839130 |
| C | -0.661018 | -1.071840 | 2.449240 |
| H | 1.608030 | 0.142178 | 0.133207 |
| H | -0.169456 | -0.683682 | -1.459360 |
| H | 2.277750 | 0.738028 | 2.375750 |
| H | -2.473980 | -1.309870 | -1.870390 |
| H | -1.296290 | -1.656420 | 1.783590 |
| H | -1.498390 | 1.032930 | 1.662520 |
| H | 2.090290 | 0.393509 | 4.828750 |
| H | -4.885410 | -1.024430 | -1.379980 |
| H | -1.577000 | -1.829620 | 4.250350 |
| H | -3.907810 | 1.140830 | 2.235980 |
| H | 0.146636 | -0.886254 | 5.769680 |
| H | -5.612570 | 0.201211 | 0.687848 |

***DHP* $\mathbf{S_1}$min**

$E_{\text{XMS-SA2-CASPT2(2,2)/SVP}}$ = -538.4324089019 a.u.

| | | | |
|---|---|---|---|
| C | 1.446310 | -0.892744 | 0.022113 |
| C | -1.488510 | -0.882578 | -0.020003 |
| C | 0.661966 | -2.071820 | 0.115260 |
| C | -0.726291 | -2.073260 | -0.068546 |
| C | 2.769940 | -0.821083 | 0.525519 |
| C | -2.784350 | -0.800422 | -0.588853 |
| C | 0.851906 | 0.335737 | -0.539715 |
| C | -0.870272 | 0.333501 | 0.536053 |
| C | 3.443460 | 0.390553 | 0.633635 |
| C | -3.446490 | 0.415826 | -0.688729 |
| C | 1.592080 | 1.555740 | -0.435392 |
| C | -1.597990 | 1.557980 | 0.417379 |
| C | 2.827590 | 1.595690 | 0.197512 |
| C | -2.834100 | 1.606520 | -0.210005 |
| H | 1.172100 | -3.022430 | 0.310913 |
| H | -1.253820 | -3.014690 | -0.268283 |
| H | 3.240050 | -1.742880 | 0.884968 |
| H | -3.227480 | -1.719490 | -0.988027 |
| H | 0.230911 | 0.213491 | -1.438330 |
| H | -0.268402 | 0.192619 | 1.446770 |
| H | 4.450130 | 0.412097 | 1.062540 |
| H | -4.438790 | 0.461297 | -1.145400 |
| H | 1.161430 | 2.462010 | -0.875585 |
| H | -1.153800 | 2.474910 | 0.817321 |
| H | 3.367810 | 2.542710 | 0.297400 |

| | | | |
|---|---|---|---|
| H | -3.375780 | 2.555680 | -0.258277 |

**OBF1**

$E_{\text{XMS-SA2-CASPT2(2,2)/SVP}}$ = -538.4041474245 a.u.

| | | | |
|---|---|---|---|
| C | 0.370667 | -0.627048 | 0.519134 |
| C | 0.136724 | -0.238753 | 1.907310 |
| C | -0.731855 | 0.840855 | 2.200110 |
| C | -1.018090 | 1.192800 | 3.521520 |
| C | -0.451933 | 0.485815 | 4.588400 |
| C | 0.406420 | -0.587144 | 4.311700 |
| C | 0.690075 | -0.951887 | 2.995520 |
| C | 0.721306 | 0.249377 | -0.527764 |
| H | 1.549270 | -0.662281 | 0.470340 |
| H | 1.392180 | 1.123980 | -0.357759 |
| C | 0.364598 | 0.062538 | -1.916320 |
| C | 0.869721 | 0.937978 | -2.902750 |
| C | 0.465204 | 0.802382 | -4.229850 |
| C | -0.430729 | -0.216565 | -4.582800 |
| C | -0.928962 | -1.097950 | -3.609340 |
| C | -0.530312 | -0.968607 | -2.282290 |
| H | -1.188690 | 1.395070 | 1.374930 |
| H | -1.691900 | 2.031250 | 3.717790 |
| H | -0.677932 | 0.763356 | 5.620010 |
| H | 0.854568 | -1.152850 | 5.132860 |
| H | 1.344190 | -1.805000 | 2.793730 |
| H | 1.567700 | 1.727880 | -2.611560 |
| H | 0.848605 | 1.481710 | -4.993770 |
| H | -0.741775 | -0.326902 | -5.624240 |
| H | -1.623870 | -1.889140 | -3.898310 |
| H | -0.873612 | -1.637510 | -1.488260 |

**OBF2**

$E_{\text{XMS-SA2-CASPT2(2,2)/SVP}}$ = -538.3969449399 a.u.

| | | | |
|---|---|---|---|
| C | -0.014453 | 0.166483 | -0.050650 |
| C | 0.007787 | -0.003223 | 1.387380 |
| C | 1.251510 | -0.191708 | 2.044080 |
| C | 1.315620 | -0.314589 | 3.432970 |
| C | 0.156380 | -0.245855 | 4.215930 |
| C | -1.076520 | -0.048684 | 3.579840 |
| C | -1.153940 | 0.078830 | 2.192460 |
| C | 0.453612 | -0.666348 | -1.087400 |
| H | -0.918525 | -0.504758 | -0.424426 |
| H | 0.722820 | -0.196421 | -2.053830 |
| C | 0.545955 | -2.127370 | -1.134270 |
| C | 1.031280 | -2.733880 | -2.311380 |
| C | 1.235500 | -4.113180 | -2.364640 |
| C | 0.955788 | -4.900310 | -1.240750 |
| C | 0.470356 | -4.307020 | -0.065064 |
| C | 0.276103 | -2.928860 | -0.000141 |
| H | 2.165960 | -0.242970 | 1.446520 |

| | | | |
|---|---|---|---|
| H | 2.288300 | -0.462897 | 3.909970 |
| H | 0.212544 | -0.340943 | 5.302190 |
| H | -1.992670 | 0.012573 | 4.173210 |
| H | -2.123130 | 0.247164 | 1.713790 |
| H | 1.244060 | -2.109600 | -3.183790 |
| H | 1.609710 | -4.576960 | -3.279610 |
| H | 1.113040 | -5.980510 | -1.279710 |
| H | 0.257695 | -4.925900 | 0.809140 |
| H | -0.087626 | -2.461760 | 0.917264 |

**HT1**

$E_{\text{XMS-SA2-CASPT2(2,2)/SVP}}$ = -538.4068716493 a.u.

| | | | |
|---|---|---|---|
| C | 0.000867 | -0.046274 | -0.041228 |
| C | -0.028684 | -0.054713 | 1.505140 |
| C | 1.179500 | 0.208282 | 2.189080 |
| C | 1.278580 | 0.037737 | 3.573940 |
| C | 0.154564 | -0.359599 | 4.302890 |
| C | -1.071390 | -0.588461 | 3.650280 |
| C | -1.172890 | -0.395464 | 2.277330 |
| C | -0.184556 | 1.355960 | 0.201179 |
| H | -0.884045 | -0.614415 | -0.364374 |
| H | -1.067050 | 1.789190 | 0.728045 |
| C | 0.790283 | 2.378100 | -0.149023 |
| C | 0.576162 | 3.726260 | 0.209748 |
| C | 1.476240 | 4.710870 | -0.196287 |
| C | 2.604960 | 4.356000 | -0.949313 |
| C | 2.827930 | 3.016580 | -1.304930 |
| C | 1.936190 | 2.026710 | -0.896011 |
| H | 2.045930 | 0.508427 | 1.595200 |
| H | 2.226720 | 0.222461 | 4.083620 |
| H | 0.226711 | -0.499286 | 5.384090 |
| H | -1.945190 | -0.899085 | 4.227760 |
| H | -2.124980 | -0.554260 | 1.762810 |
| H | -0.308583 | 3.988130 | 0.797472 |
| H | 1.308390 | 5.755130 | 0.076386 |
| H | 3.313690 | 5.127060 | -1.260880 |
| H | 3.709450 | 2.750680 | -1.892920 |
| H | 2.061070 | 0.965378 | -1.130940 |

**HT2**

$E_{\text{XMS-SA2-CASPT2(2,2)/SVP}}$ = -538.3963218573 a.u.

| | | | |
|---|---|---|---|
| C | 1.652980 | -0.174617 | 0.210318 |
| C | 0.496207 | -0.112937 | 1.113920 |
| C | -0.194365 | 1.090650 | 1.363890 |
| C | -1.254500 | 1.118550 | 2.269670 |
| C | -1.655060 | -0.053718 | 2.926020 |
| C | -0.978280 | -1.255820 | 2.684080 |
| C | 0.084412 | -1.285280 | 1.780310 |
| C | 2.239160 | 0.953124 | -0.562100 |
| H | 2.209970 | -1.116920 | 0.325945 |

| | | | |
|---|---|---|---|
| H | 2.119050 | 1.956480 | -0.131574 |
| C | 1.186780 | 0.587256 | -1.465720 |
| C | 0.026649 | 1.368750 | -1.813570 |
| C | -1.118900 | 0.764302 | -2.282950 |
| C | -1.220500 | -0.654487 | -2.392650 |
| C | -0.102347 | -1.434990 | -2.161520 |
| C | 1.109590 | -0.832306 | -1.744660 |
| H | 0.126685 | 2.003050 | 0.859367 |
| H | -1.777340 | 2.057600 | 2.464750 |
| H | -2.487320 | -0.027182 | 3.632960 |
| H | -1.285550 | -2.170030 | 3.196700 |
| H | 0.617050 | -2.220860 | 1.586160 |
| H | 0.076017 | 2.453260 | -1.682690 |
| H | -1.983300 | 1.380050 | -2.546550 |
| H | -2.156980 | -1.107190 | -2.724870 |
| H | -0.134424 | -2.513600 | -2.335530 |
| H | 2.041840 | -1.396540 | -1.785580 |

**CI$_{\mathrm{DHP}}$**

$E_{\text{XMS-SA2-CASPT2(2,2)/SVP}}$ = -538.4059241285 a.u.

| | | | |
|---|---|---|---|
| C | 1.465710 | -0.888798 | -0.023035 |
| C | -1.455070 | -0.851127 | -0.065108 |
| C | 0.688560 | -2.054690 | -0.041162 |
| C | -0.680001 | -2.033060 | -0.331431 |
| C | 2.787110 | -0.765668 | 0.490845 |
| C | -2.862510 | -0.849846 | 0.086296 |
| C | 0.798670 | 0.265781 | -0.697984 |
| C | -0.844428 | 0.427581 | 0.113673 |
| C | 3.492950 | 0.412295 | 0.410079 |
| C | -3.566250 | 0.349983 | 0.221154 |
| C | 1.580540 | 1.511390 | -0.720481 |
| C | -1.538580 | 1.629780 | 0.303460 |
| C | 2.862470 | 1.529080 | -0.229386 |
| C | -2.932250 | 1.603340 | 0.316041 |
| H | 1.160260 | -3.007180 | 0.234184 |
| H | -1.230210 | -2.976270 | -0.383044 |
| H | 3.224380 | -1.647610 | 0.970904 |
| H | -3.410010 | -1.795180 | 0.051889 |
| H | 0.285899 | -0.059361 | -1.631540 |
| H | 0.141068 | 0.379507 | 0.679077 |
| H | 4.507080 | 0.500471 | 0.800052 |
| H | -4.658350 | 0.306434 | 0.265226 |
| H | 1.174390 | 2.381800 | -1.241230 |
| H | -0.971122 | 2.556920 | 0.432315 |
| H | 3.433810 | 2.455170 | -0.349846 |
| H | -3.517890 | 2.520870 | 0.389066 |

## II.C XMS-SA3-CASPT2(2,2)/SVP

***trans*** $\mathbf{S_0}$**min**

$E_{\text{XMS-SA3-CASPT2(2,2)/SVP}}$ = -538.5666967864 a.u.

| | | | |
|---|---|---|---|
| C | 1.968800 | -0.025289 | 0.066578 |
| C | -1.920630 | -0.048860 | -0.029840 |
| C | 0.554451 | 0.017270 | 0.443570 |
| C | -0.504357 | -0.091591 | -0.400837 |
| C | 2.938770 | -0.224992 | 1.069850 |
| C | -2.887830 | -0.431430 | -0.981637 |
| C | 2.414820 | 0.131213 | -1.264480 |
| C | -2.372120 | 0.363446 | 1.243800 |
| C | 4.299790 | -0.285223 | 0.760268 |
| C | -4.250870 | -0.419307 | -0.675157 |
| C | 3.772540 | 0.063507 | -1.574970 |
| C | -3.731700 | 0.368547 | 1.552120 |
| C | 4.724340 | -0.143008 | -0.565647 |
| C | -4.680660 | -0.020274 | 0.595295 |
| H | 0.364667 | 0.115332 | 1.518160 |
| H | -0.310796 | -0.255749 | -1.467140 |
| H | 2.611480 | -0.338419 | 2.107370 |
| H | -2.556670 | -0.745774 | -1.975860 |
| H | 1.691860 | 0.321717 | -2.060530 |
| H | -1.651530 | 0.693862 | 1.994720 |
| H | 5.031400 | -0.441492 | 1.556540 |
| H | -4.979700 | -0.720190 | -1.430990 |
| H | 4.098620 | 0.187176 | -2.610230 |
| H | -4.060520 | 0.690257 | 2.542780 |
| H | 5.787800 | -0.185493 | -0.812150 |
| H | -5.745370 | -0.005878 | 0.839034 |

***cis*** $\mathbf{S_0}$**min**

$E_{\text{XMS-SA3-CASPT2(2,2)/SVP}}$ = -538.5644065284 a.u.

| | | | |
|---|---|---|---|
| C | -4.592140 | 0.060371 | 0.475832 |
| C | -4.106730 | -0.607093 | -0.653447 |
| C | -2.736540 | -0.602027 | -0.935702 |
| C | -1.828420 | 0.052511 | -0.083911 |
| C | -2.331740 | 0.740326 | 1.039990 |
| C | -3.698760 | 0.738571 | 1.317800 |
| C | -0.389384 | 0.043316 | -0.396309 |
| C | 0.624106 | -0.133206 | 0.490940 |
| C | 0.484298 | -0.459462 | 1.919880 |
| C | 1.310050 | 0.171394 | 2.867810 |
| C | 1.189860 | -0.121514 | 4.230220 |
| C | 0.254101 | -1.065860 | 4.663930 |
| C | -0.555956 | -1.720840 | 3.725060 |
| C | -0.440447 | -1.426050 | 2.366670 |
| H | 1.649600 | -0.034259 | 0.116471 |
| H | -0.122063 | 0.205420 | -1.446990 |
| H | 2.044120 | 0.909116 | 2.531750 |
| H | -2.360310 | -1.123430 | -1.820610 |

| | | | |
|---|---|---|---|
| H | -1.063740 | -1.947020 | 1.636540 |
| H | -1.639420 | 1.279570 | 1.690600 |
| H | 1.832720 | 0.386008 | 4.953200 |
| H | -4.796360 | -1.129380 | -1.320740 |
| H | -1.275260 | -2.473350 | 4.056310 |
| H | -4.074510 | 1.277300 | 2.190940 |
| H | 0.160895 | -1.299940 | 5.726680 |
| H | -5.661960 | 0.061858 | 0.696419 |

**DHP $S_0$min**

$E_{\text{XMS-SA3-CASPT2(2,2)/SVP}}$ = -538.5666967864 a.u.

| | | | |
|---|---|---|---|
| C | 1.433220 | -0.868487 | -0.022708 |
| C | -1.438270 | -0.892597 | -0.066514 |
| C | 0.718041 | -2.047390 | -0.022054 |
| C | -0.702133 | -2.054730 | -0.160454 |
| C | 2.864470 | -0.848762 | 0.085275 |
| C | -2.870910 | -0.891564 | -0.158222 |
| C | 0.692659 | 0.433135 | -0.296632 |
| C | -0.720365 | 0.398703 | 0.300181 |
| C | 3.546840 | 0.328203 | 0.244620 |
| C | -3.575740 | 0.281242 | -0.222701 |
| C | 1.485140 | 1.639850 | 0.131960 |
| C | -1.535780 | 1.618290 | -0.040394 |
| C | 2.827020 | 1.582680 | 0.326454 |
| C | -2.878520 | 1.551150 | -0.225037 |
| H | 1.257360 | -2.997340 | 0.053313 |
| H | -1.225080 | -3.005810 | -0.305286 |
| H | 3.395650 | -1.805360 | 0.112512 |
| H | -3.385750 | -1.853190 | -0.248963 |
| H | 0.565150 | 0.493170 | -1.405620 |
| H | -0.593956 | 0.381032 | 1.410910 |
| H | 4.633450 | 0.322915 | 0.358256 |
| H | -4.663620 | 0.264336 | -0.322156 |
| H | 0.976469 | 2.603340 | 0.204695 |
| H | -1.043320 | 2.592820 | -0.054170 |
| H | 3.377120 | 2.491910 | 0.582090 |
| H | -3.446740 | 2.465670 | -0.413401 |

***twist* $S_1$min**

$E_{\text{XMS-SA3-CASPT2(2,2)/SVP}}$ = -538.4260939076 a.u.

| | | | |
|---|---|---|---|
| C | 1.703210 | -0.245139 | 0.213576 |
| C | -1.667680 | -0.527675 | -0.124832 |
| C | 0.509416 | -0.931777 | 0.547038 |
| C | -0.488387 | -1.347830 | -0.422754 |
| C | 2.603820 | 0.205396 | 1.214580 |
| C | -2.983840 | -1.066580 | -0.188183 |
| C | 1.988690 | -0.020467 | -1.160550 |
| C | -1.558790 | 0.845027 | 0.238561 |
| C | 3.770320 | 0.862262 | 0.845203 |
| C | -4.102920 | -0.279074 | 0.060405 |

| | | | |
|---|---|---|---|
| C | 3.162020 | 0.631044 | -1.520520 |
| C | -2.692020 | 1.620080 | 0.514989 |
| C | 4.044700 | 1.070180 | -0.519081 |
| C | -3.973800 | 1.071050 | 0.434429 |
| H | 0.363544 | -1.086730 | 1.640680 |
| H | -0.730315 | -2.408830 | -0.200697 |
| H | 2.361130 | 0.030262 | 2.266390 |
| H | -3.107750 | -2.117370 | -0.467292 |
| H | 1.234340 | -0.398354 | -1.862760 |
| H | -0.575584 | 1.327020 | 0.244833 |
| H | 4.471790 | 1.217600 | 1.602070 |
| H | -5.097870 | -0.726904 | -0.021804 |
| H | 3.400840 | 0.805570 | -2.571280 |
| H | -2.564000 | 2.671270 | 0.790518 |
| H | 4.965670 | 1.584990 | -0.804676 |
| H | -4.858340 | 1.678560 | 0.639603 |

***trans* $\mathbf{S_1}$min**
$E_{\text{XMS-SA3-CASPT2(2,2)/SVP}}$ = -538.4122098417 a.u.

| | | | |
|---|---|---|---|
| C | 1.914630 | -0.087337 | 0.114255 |
| C | -1.860800 | -0.246935 | -0.055602 |
| C | 0.581221 | -0.299022 | 0.507383 |
| C | -0.512730 | -0.451176 | -0.399686 |
| C | 2.983960 | -0.169012 | 1.067090 |
| C | -2.914980 | -0.635694 | -0.947506 |
| C | 2.265080 | 0.190439 | -1.254830 |
| C | -2.239470 | 0.329702 | 1.208740 |
| C | 4.304630 | -0.022302 | 0.675023 |
| C | -4.245220 | -0.494618 | -0.587134 |
| C | 3.593890 | 0.342428 | -1.626920 |
| C | -3.578080 | 0.470517 | 1.548240 |
| C | 4.625360 | 0.226256 | -0.677133 |
| C | -4.592040 | 0.051711 | 0.667503 |
| H | 0.381654 | -0.432766 | 1.580330 |
| H | -0.295464 | -0.827035 | -1.409870 |
| H | 2.737790 | -0.374790 | 2.113800 |
| H | -2.647750 | -1.071680 | -1.915600 |
| H | 1.465120 | 0.306912 | -1.992200 |
| H | -1.455050 | 0.680375 | 1.886100 |
| H | 5.103070 | -0.104062 | 1.417220 |
| H | -5.030510 | -0.812850 | -1.277710 |
| H | 3.842190 | 0.549778 | -2.671560 |
| H | -3.847210 | 0.907313 | 2.513880 |
| H | 5.669690 | 0.327291 | -0.984691 |
| H | -5.642870 | 0.148060 | 0.953231 |

***cis* $\mathbf{S_1}$min**
$E_{\text{XMS-SA3-CASPT2(2,2)/SVP}}$ = -538.4318057045 a.u.

| | | | |
|---|---|---|---|
| C | -4.283590 | 0.026476 | 0.570406 |
| C | -3.952190 | -0.639807 | -0.638831 |

| | | | |
|---|---|---|---|
| C | -2.622520 | -0.798355 | -1.007960 |
| C | -1.567770 | -0.363257 | -0.171476 |
| C | -1.903960 | 0.345651 | 1.078430 |
| C | -3.283790 | 0.567126 | 1.367600 |
| C | -0.210386 | -0.667321 | -0.445292 |
| C | 0.774206 | -0.576843 | 0.540679 |
| C | 0.448037 | -0.527560 | 1.917920 |
| C | 1.358450 | -0.067814 | 2.896650 |
| C | 0.969311 | 0.130633 | 4.214860 |
| C | -0.353237 | -0.183612 | 4.625990 |
| C | -1.244150 | -0.749041 | 3.727370 |
| C | -0.918642 | -0.887014 | 2.342510 |
| H | 1.832300 | -0.551940 | 0.251979 |
| H | 0.068024 | -0.959720 | -1.463500 |
| H | 2.379590 | 0.173143 | 2.586120 |
| H | -2.367850 | -1.302880 | -1.943630 |
| H | -1.378510 | -1.735970 | 1.819260 |
| H | -1.212770 | 1.149570 | 1.370140 |
| H | 1.686200 | 0.527378 | 4.936150 |
| H | -4.743820 | -1.020730 | -1.291060 |
| H | -2.217010 | -1.108680 | 4.071640 |
| H | -3.549060 | 1.190870 | 2.228110 |
| H | -0.634611 | -0.063408 | 5.675970 |
| H | -5.333650 | 0.181297 | 0.828891 |

***DHP* $\mathbf{S_1}$min**

$E_{\text{XMS-SA3-CASPT2(2,2)/SVP}}$ = -538.4480922575 a.u.

| | | | |
|---|---|---|---|
| C | 1.451640 | -0.880003 | -0.022893 |
| C | -1.444160 | -0.924074 | -0.067859 |
| C | 0.720633 | -2.090480 | -0.010088 |
| C | -0.675382 | -2.106170 | -0.174667 |
| C | 2.764460 | -0.776170 | 0.490299 |
| C | -2.762130 | -0.822946 | -0.568166 |
| C | 0.747205 | 0.351077 | -0.482402 |
| C | -0.777613 | 0.289432 | 0.485354 |
| C | 3.387380 | 0.459646 | 0.647089 |
| C | -3.423910 | 0.401133 | -0.628307 |
| C | 1.473660 | 1.595230 | -0.344781 |
| C | -1.542680 | 1.517130 | 0.444214 |
| C | 2.720380 | 1.649450 | 0.253899 |
| C | -2.792730 | 1.577940 | -0.146817 |
| H | 1.261390 | -3.033240 | 0.128932 |
| H | -1.186490 | -3.051960 | -0.386288 |
| H | 3.270500 | -1.690570 | 0.815928 |
| H | -3.241220 | -1.725380 | -0.961076 |
| H | 0.249685 | 0.230211 | -1.459050 |
| H | -0.274768 | 0.108809 | 1.449960 |
| H | 4.390280 | 0.515251 | 1.077980 |
| H | -4.430120 | 0.457947 | -1.051120 |
| H | 1.004410 | 2.511500 | -0.717548 |
| H | -1.100910 | 2.416190 | 0.886069 |
| H | 3.222850 | 2.613800 | 0.367932 |

| | | | |
|---|---|---|---|
| H | -3.325340 | 2.532010 | -0.186162 |

**OBF1**

$E_{\text{XMS-SA3-CASPT2(2,2)/SVP}}$ = -538.4230998286 a.u.

| | | | |
|---|---|---|---|
| C | 0.457665 | -0.776576 | 0.641982 |
| C | 0.068896 | -0.122880 | 1.899400 |
| C | -0.715106 | 1.056770 | 1.877980 |
| C | -1.147180 | 1.658060 | 3.064280 |
| C | -0.812350 | 1.111540 | 4.307780 |
| C | -0.038408 | -0.057210 | 4.342610 |
| C | 0.391759 | -0.667912 | 3.164050 |
| C | 0.921508 | 0.017643 | -0.453763 |
| H | 1.588760 | -0.950066 | 0.819706 |
| H | 1.670430 | 0.829252 | -0.312291 |
| C | 0.562236 | -0.210030 | -1.820640 |
| C | 1.117540 | 0.578961 | -2.855260 |
| C | 0.688770 | 0.403669 | -4.168430 |
| C | -0.282901 | -0.567915 | -4.453380 |
| C | -0.835160 | -1.364060 | -3.435360 |
| C | -0.411205 | -1.194020 | -2.122320 |
| H | -1.005080 | 1.488350 | 0.913735 |
| H | -1.752430 | 2.567860 | 3.012360 |
| H | -1.152740 | 1.581980 | 5.233090 |
| H | 0.231110 | -0.503068 | 5.304260 |
| H | 0.980162 | -1.589420 | 3.210600 |
| H | 1.872110 | 1.330820 | -2.607470 |
| H | 1.106270 | 1.011520 | -4.973310 |
| H | -0.613385 | -0.709163 | -5.485350 |
| H | -1.587440 | -2.116130 | -3.681270 |
| H | -0.778370 | -1.785770 | -1.276460 |

**OBF2**

$E_{\text{XMS-SA3-CASPT2(2,2)/SVP}}$ = -538.4146370393 a.u.

| | | | |
|---|---|---|---|
| C | -0.020635 | 0.193945 | -0.060976 |
| C | 0.007562 | -0.001777 | 1.378120 |
| C | 1.246670 | -0.235780 | 2.030870 |
| C | 1.310290 | -0.385372 | 3.417570 |
| C | 0.156103 | -0.305961 | 4.207630 |
| C | -1.071340 | -0.066053 | 3.575520 |
| C | -1.149490 | 0.089808 | 2.190340 |
| C | 0.531348 | -0.651851 | -1.069860 |
| H | -0.955743 | -0.407999 | -0.416241 |
| H | 0.905261 | -0.175393 | -1.998420 |
| C | 0.570777 | -2.099390 | -1.132350 |
| C | 1.113300 | -2.715700 | -2.281570 |
| C | 1.279280 | -4.099700 | -2.333240 |
| C | 0.905731 | -4.879620 | -1.232040 |
| C | 0.364861 | -4.281540 | -0.081271 |
| C | 0.206376 | -2.900800 | -0.020331 |
| H | 2.161890 | -0.294931 | 1.433260 |

| | | | |
|---|---|---|---|
| H | 2.281600 | -0.564824 | 3.887860 |
| H | 0.212641 | -0.422039 | 5.292190 |
| H | -1.985970 | 0.005729 | 4.171000 |
| H | -2.116950 | 0.291470 | 1.719580 |
| H | 1.399260 | -2.091500 | -3.133190 |
| H | 1.695830 | -4.572260 | -3.224950 |
| H | 1.033450 | -5.963950 | -1.267970 |
| H | 0.083665 | -4.900510 | 0.773087 |
| H | -0.197825 | -2.422350 | 0.874372 |

**HT1**

$E_{\text{XMS-SA3-CASPT2(2,2)/SVP}}$ = -538.4263064209 a.u.

| | | | |
|---|---|---|---|
| C | 0.030547 | 0.014700 | 0.002525 |
| C | -0.048362 | -0.094175 | 1.505470 |
| C | 0.975214 | 0.451799 | 2.319120 |
| C | 0.943549 | 0.324588 | 3.713990 |
| C | -0.121200 | -0.328548 | 4.336060 |
| C | -1.159760 | -0.861788 | 3.550500 |
| C | -1.156260 | -0.694866 | 2.169580 |
| C | -0.145403 | 1.452340 | 0.145088 |
| H | -0.878923 | -0.463851 | -0.405684 |
| H | -1.067580 | 1.899000 | 0.579569 |
| C | 0.869132 | 2.419130 | -0.176145 |
| C | 0.674979 | 3.795460 | 0.083123 |
| C | 1.641410 | 4.719830 | -0.305814 |
| C | 2.811410 | 4.276040 | -0.942403 |
| C | 3.016520 | 2.911100 | -1.200910 |
| C | 2.056090 | 1.981560 | -0.811330 |
| H | 1.837470 | 0.917052 | 1.835610 |
| H | 1.757890 | 0.739944 | 4.312980 |
| H | -0.141537 | -0.441510 | 5.422450 |
| H | -1.994110 | -1.379380 | 4.031220 |
| H | -1.977150 | -1.092820 | 1.566190 |
| H | -0.242342 | 4.119400 | 0.583280 |
| H | 1.496480 | 5.784640 | -0.111589 |
| H | 3.571640 | 5.002690 | -1.239470 |
| H | 3.932520 | 2.583100 | -1.697350 |
| H | 2.147120 | 0.900080 | -0.967140 |

**HT2**

$E_{\text{XMS-SA3-CASPT2(2,2)/SVP}}$ = -538.4151678598 a.u.

| | | | |
|---|---|---|---|
| C | 1.660660 | -0.175413 | 0.211270 |
| C | 0.569640 | -0.123499 | 1.176370 |
| C | -0.223067 | 1.032860 | 1.349720 |
| C | -1.200250 | 1.071390 | 2.340830 |
| C | -1.408420 | -0.040627 | 3.172050 |
| C | -0.629638 | -1.192340 | 3.011690 |
| C | 0.346819 | -1.235430 | 2.015360 |
| C | 2.116110 | 0.918662 | -0.677268 |
| H | 2.238760 | -1.110780 | 0.286854 |

| | | | |
|---|---|---|---|
| H | 2.033300 | 1.937450 | -0.271997 |
| C | 1.043960 | 0.539076 | -1.552350 |
| C | -0.064143 | 1.366030 | -1.950040 |
| C | -1.209570 | 0.810177 | -2.479930 |
| C | -1.370970 | -0.602172 | -2.589450 |
| C | -0.300799 | -1.426880 | -2.283330 |
| C | 0.914165 | -0.878208 | -1.817290 |
| H | -0.053618 | 1.892780 | 0.700353 |
| H | -1.809110 | 1.968910 | 2.470340 |
| H | -2.176300 | -0.004287 | 3.948110 |
| H | -0.790118 | -2.057290 | 3.658890 |
| H | 0.957489 | -2.132870 | 1.879660 |
| H | 0.025237 | 2.449890 | -1.827340 |
| H | -2.026980 | 1.464870 | -2.795460 |
| H | -2.306100 | -1.016860 | -2.972090 |
| H | -0.374773 | -2.505970 | -2.444170 |
| H | 1.811770 | -1.498570 | -1.789450 |

**$\mathbf{CI_{DHP}}$**

$E_{\text{XMS-SA3-CASPT2(2,2)/SVP}}$ = -538.4314347944 a.u.

| | | | |
|---|---|---|---|
| C | 0.480205 | -0.285504 | -1.431160 |
| C | 0.580525 | -1.278400 | -0.432452 |
| C | 0.658062 | -0.918579 | 0.912021 |
| C | 0.866535 | 0.532071 | 1.100290 |
| C | 1.048680 | 0.948943 | 2.474330 |
| C | 0.861920 | 0.047424 | 3.504380 |
| C | 0.380989 | -1.771830 | 2.016330 |
| C | 0.465444 | -1.309620 | 3.314260 |
| C | -0.352192 | 0.841044 | -1.126460 |
| C | -0.956004 | 1.650350 | -2.117370 |
| C | -0.625504 | 1.274710 | 0.238643 |
| C | -1.320880 | 2.965070 | -1.803070 |
| C | -1.123420 | 2.565040 | 0.529845 |
| C | -1.366160 | 3.477510 | -0.491583 |
| H | 0.534950 | -0.578370 | -2.484400 |
| H | 0.546026 | -2.345670 | -0.699101 |
| H | 1.537940 | 0.927709 | 0.304783 |
| H | 1.367190 | 1.972180 | 2.691460 |
| H | 1.063060 | 0.394856 | 4.523360 |
| H | 0.115351 | -2.813640 | 1.806100 |
| H | 0.297667 | -1.965070 | 4.170150 |
| H | -0.973888 | 1.320860 | -3.159370 |
| H | -1.044630 | 0.498893 | 0.901990 |
| H | -1.589220 | 3.626680 | -2.634020 |
| H | -1.313220 | 2.814290 | 1.579870 |
| H | -1.630090 | 4.518050 | -0.299183 |

## II.D XMS-SA2-CASPT2(2,2)/cc-pVDZ

***trans* $S_0$min**

$E_{\text{XMS-SA2-CASPT2(2,2)/cc-pVDZ}}$ = -538.9962001843 a.u.

| | | | |
|---|---|---|---|
| C | 1.973420 | -0.025222 | 0.066696 |
| C | -1.925110 | -0.048195 | -0.029896 |
| C | 0.554656 | 0.015015 | 0.447674 |
| C | -0.504969 | -0.088515 | -0.405083 |
| C | 2.946140 | -0.229360 | 1.072460 |
| C | -2.895160 | -0.424754 | -0.987387 |
| C | 2.418260 | 0.136700 | -1.268110 |
| C | -2.375280 | 0.355349 | 1.251040 |
| C | 4.311640 | -0.290131 | 0.760121 |
| C | -4.262770 | -0.415649 | -0.678435 |
| C | 3.781880 | 0.073080 | -1.581110 |
| C | -3.740750 | 0.361569 | 1.561310 |
| C | 4.736500 | -0.141077 | -0.570063 |
| C | -4.692800 | -0.023606 | 0.599542 |
| H | 0.361843 | 0.103191 | 1.524740 |
| H | -0.308569 | -0.247624 | -1.473370 |
| H | 2.618160 | -0.347916 | 2.111240 |
| H | -2.563460 | -0.732795 | -1.985290 |
| H | 1.693560 | 0.331719 | -2.064700 |
| H | -1.653360 | 0.681492 | 2.006110 |
| H | 5.045350 | -0.452699 | 1.556180 |
| H | -4.994120 | -0.713784 | -1.436620 |
| H | 4.106740 | 0.202730 | -2.618470 |
| H | -4.068960 | 0.678088 | 2.556540 |
| H | 5.802040 | -0.183242 | -0.817895 |
| H | -5.759790 | -0.010986 | 0.844186 |

***cis* $S_0$min**

$E_{\text{XMS-SA2-CASPT2(2,2)/cc-pVDZ}}$ = -538.9932788236 a.u.

| | | | |
|---|---|---|---|
| C | -4.648620 | 0.067637 | 0.455558 |
| C | -4.170390 | -0.602122 | -0.682398 |
| C | -2.793990 | -0.626660 | -0.957137 |
| C | -1.871190 | -0.002478 | -0.089881 |
| C | -2.366590 | 0.686206 | 1.042790 |
| C | -3.741250 | 0.717625 | 1.311800 |
| C | -0.427885 | -0.034847 | -0.408301 |
| C | 0.601129 | -0.180051 | 0.472703 |
| C | 0.496695 | -0.455072 | 1.921620 |
| C | 1.389070 | 0.169674 | 2.819790 |
| C | 1.317050 | -0.082999 | 4.198870 |
| C | 0.363145 | -0.983632 | 4.700090 |
| C | -0.512760 | -1.633800 | 3.811770 |
| C | -0.447071 | -1.375540 | 2.436160 |
| H | 1.620850 | -0.096240 | 0.073175 |
| H | -0.168717 | 0.081147 | -1.469320 |
| H | 2.137790 | 0.869582 | 2.431230 |
| H | -2.424330 | -1.146190 | -1.848640 |

| | | | |
|---|---|---|---|
| H | -1.120160 | -1.894560 | 1.746760 |
| H | -1.665570 | 1.206120 | 1.702950 |
| H | 2.010960 | 0.418877 | 4.881090 |
| H | -4.869820 | -1.100680 | -1.361330 |
| H | -1.245490 | -2.352230 | 4.193700 |
| H | -4.109110 | 1.258710 | 2.189700 |
| H | 0.308990 | -1.187530 | 5.774290 |
| H | -5.721940 | 0.093831 | 0.668983 |

**DHP $S_0$min**

$E_{\text{XMS-SA2-CASPT2(2,2)/cc-pVDZ}}$ = -538.9340775845 a.u.

| | | | |
|---|---|---|---|
| C | 1.435570 | -0.869953 | -0.022732 |
| C | -1.443960 | -0.890498 | -0.066515 |
| C | 0.718576 | -2.054010 | -0.026957 |
| C | -0.707691 | -2.058400 | -0.154915 |
| C | 2.875040 | -0.853374 | 0.060149 |
| C | -2.882220 | -0.891119 | -0.172819 |
| C | 0.701028 | 0.443406 | -0.264806 |
| C | -0.731660 | 0.407073 | 0.296072 |
| C | 3.559360 | 0.323159 | 0.247180 |
| C | -3.585990 | 0.284416 | -0.263907 |
| C | 1.486260 | 1.632570 | 0.237384 |
| C | -1.535410 | 1.623270 | -0.101347 |
| C | 2.834610 | 1.573030 | 0.417606 |
| C | -2.879840 | 1.556620 | -0.306125 |
| H | 1.262130 | -3.005170 | 0.025552 |
| H | -1.236100 | -3.009600 | -0.294958 |
| H | 3.410820 | -1.809900 | 0.036792 |
| H | -3.400040 | -1.855330 | -0.244607 |
| H | 0.605888 | 0.545928 | -1.377600 |
| H | -0.635240 | 0.413252 | 1.413350 |
| H | 4.650580 | 0.317287 | 0.333785 |
| H | -4.675360 | 0.268380 | -0.370350 |
| H | 0.964067 | 2.582020 | 0.391121 |
| H | -1.030890 | 2.593090 | -0.156805 |
| H | 3.383570 | 2.467410 | 0.730580 |
| H | -3.440550 | 2.467370 | -0.541646 |

***twist* $S_1$min**

$E_{\text{XMS-SA2-CASPT2(2,2)/cc-pVDZ}}$ = -538.8495479671 a.u.

| | | | |
|---|---|---|---|
| C | 1.692960 | -0.242767 | 0.212406 |
| C | -1.675790 | -0.521370 | -0.125949 |
| C | 0.485493 | -0.919548 | 0.544588 |
| C | -0.494442 | -1.365940 | -0.425453 |
| C | 2.570980 | 0.241527 | 1.226340 |
| C | -2.996080 | -1.066100 | -0.108597 |
| C | 2.021870 | -0.079355 | -1.165990 |
| C | -1.562240 | 0.875649 | 0.143745 |
| C | 3.757240 | 0.876652 | 0.864390 |
| C | -4.114640 | -0.266819 | 0.135388 |

| | | | |
|---|---|---|---|
| C | 3.217890 | 0.548484 | -1.515030 |
| C | -2.690770 | 1.667140 | 0.418943 |
| C | 4.077140 | 1.025780 | -0.503371 |
| C | -3.976310 | 1.108600 | 0.421118 |
| H | 0.335109 | -1.032860 | 1.647630 |
| H | -0.768402 | -2.410000 | -0.155651 |
| H | 2.295900 | 0.110411 | 2.278710 |
| H | -3.124320 | -2.134760 | -0.317344 |
| H | 1.291010 | -0.482102 | -1.878920 |
| H | -0.578680 | 1.357670 | 0.088937 |
| H | 4.440270 | 1.257930 | 1.628660 |
| H | -5.111720 | -0.722441 | 0.121110 |
| H | 3.493720 | 0.674639 | -2.565980 |
| H | -2.557660 | 2.735180 | 0.626345 |
| H | 5.012430 | 1.523320 | -0.781761 |
| H | -4.855550 | 1.727390 | 0.625933 |

***trans* $\mathbf{S_1}$min**

$E_{\text{XMS-SA2-CASPT2(2,2)/cc-pVDZ}}$ = -538.8557640696 a.u.

| | | | |
|---|---|---|---|
| C | 1.932920 | -0.087631 | 0.115192 |
| C | -1.886490 | -0.104174 | -0.072385 |
| C | 0.570012 | -0.113968 | 0.486909 |
| C | -0.523735 | -0.212649 | -0.429475 |
| C | 2.957350 | -0.043655 | 1.127910 |
| C | -2.911120 | -0.277392 | -1.070940 |
| C | 2.368000 | -0.108243 | -1.265450 |
| C | -2.321230 | 0.174115 | 1.280190 |
| C | 4.306130 | -0.028872 | 0.793121 |
| C | -4.259770 | -0.187382 | -0.747545 |
| C | 3.725590 | -0.094678 | -1.581170 |
| C | -3.678680 | 0.258993 | 1.585200 |
| C | 4.706690 | -0.057126 | -0.566962 |
| C | -4.659980 | 0.079616 | 0.586474 |
| H | 0.347556 | -0.079931 | 1.561600 |
| H | -0.301456 | -0.410861 | -1.486310 |
| H | 2.649280 | -0.025522 | 2.180990 |
| H | -2.603340 | -0.487758 | -2.103040 |
| H | 1.623430 | -0.119372 | -2.068570 |
| H | -1.576460 | 0.334325 | 2.067050 |
| H | 5.063500 | 0.003602 | 1.583750 |
| H | -5.017290 | -0.324568 | -1.526700 |
| H | 4.035140 | -0.112853 | -2.632860 |
| H | -3.987960 | 0.468638 | 2.616020 |
| H | 5.770380 | -0.048578 | -0.829188 |
| H | -5.723560 | 0.147454 | 0.840372 |

***cis* $\mathbf{S_1}$min**

$E_{\text{XMS-SA2-CASPT2(2,2)/cc-pVDZ}}$ = -538.8742964333 a.u.

| | | | |
|---|---|---|---|
| C | -4.297670 | 0.028987 | 0.563400 |
| C | -3.966340 | -0.641063 | -0.653988 |

| | | | |
|---|---|---|---|
| C | -2.632930 | -0.766711 | -1.030240 |
| C | -1.573510 | -0.323308 | -0.185949 |
| C | -1.907380 | 0.366193 | 1.086530 |
| C | -3.304700 | 0.577287 | 1.367550 |
| C | -0.216310 | -0.624241 | -0.460555 |
| C | 0.781351 | -0.530108 | 0.533277 |
| C | 0.456962 | -0.524392 | 1.914900 |
| C | 1.377090 | -0.074942 | 2.905800 |
| C | 0.976025 | 0.104064 | 4.227400 |
| C | -0.340001 | -0.245493 | 4.653110 |
| C | -1.221020 | -0.817872 | 3.743240 |
| C | -0.904247 | -0.913226 | 2.349490 |
| H | 1.840880 | -0.462686 | 0.256116 |
| H | 0.044425 | -0.943176 | -1.480190 |
| H | 2.396070 | 0.190769 | 2.599180 |
| H | -2.380110 | -1.262600 | -1.974510 |
| H | -1.364600 | -1.751820 | 1.808460 |
| H | -1.214070 | 1.170920 | 1.377870 |
| H | 1.687870 | 0.514601 | 4.951290 |
| H | -4.765410 | -1.031420 | -1.293180 |
| H | -2.173400 | -1.224270 | 4.101000 |
| H | -3.588230 | 1.210330 | 2.216210 |
| H | -0.617940 | -0.143108 | 5.707310 |
| H | -5.351950 | 0.175909 | 0.829713 |

***DHP*** **$S_1$min**

$E_{\text{XMS-SA2-CASPT2(2,2)/cc-pVDZ}}$ = -538.8765007046 a.u.

| | | | |
|---|---|---|---|
| C | 1.449190 | -0.894578 | 0.022173 |
| C | -1.483760 | -0.884420 | -0.019917 |
| C | 0.669292 | -2.083820 | 0.118413 |
| C | -0.723167 | -2.085470 | -0.064223 |
| C | 2.772100 | -0.820051 | 0.536180 |
| C | -2.784990 | -0.802833 | -0.580548 |
| C | 0.834239 | 0.334056 | -0.538109 |
| C | -0.867750 | 0.340005 | 0.532882 |
| C | 3.449770 | 0.394693 | 0.635164 |
| C | -3.449450 | 0.420155 | -0.685927 |
| C | 1.582390 | 1.555530 | -0.440442 |
| C | -1.595420 | 1.565540 | 0.414713 |
| C | 2.829160 | 1.594120 | 0.184163 |
| C | -2.837290 | 1.616450 | -0.217001 |
| H | 1.184140 | -3.031080 | 0.325088 |
| H | -1.256200 | -3.028340 | -0.250727 |
| H | 3.239780 | -1.743750 | 0.901617 |
| H | -3.236270 | -1.724290 | -0.977180 |
| H | 0.229598 | 0.195996 | -1.448310 |
| H | -0.264292 | 0.201549 | 1.445570 |
| H | 4.459580 | 0.421873 | 1.062810 |
| H | -4.443100 | 0.459333 | -1.145500 |
| H | 1.157860 | 2.467550 | -0.881944 |
| H | -1.152650 | 2.486270 | 0.816617 |
| H | 3.368460 | 2.545020 | 0.272258 |

| | | | |
|---|---|---|---|
| H | -3.376920 | 2.566860 | -0.282071 |

**OBF1**

$E_{\text{XMS-SA2-CASPT2(2,2)/cc-pVDZ}}$ = -538.8476899117 a.u.

| | | | |
|---|---|---|---|
| C | 0.388919 | -0.658419 | 0.544908 |
| C | 0.109589 | -0.192501 | 1.917010 |
| C | -0.749240 | 0.917852 | 2.130990 |
| C | -1.082970 | 1.331720 | 3.429510 |
| C | -0.568952 | 0.659576 | 4.550170 |
| C | 0.280057 | -0.444532 | 4.350760 |
| C | 0.608759 | -0.869924 | 3.057680 |
| C | 0.746583 | 0.221897 | -0.512714 |
| H | 1.574180 | -0.660171 | 0.567516 |
| H | 1.405170 | 1.110910 | -0.351293 |
| C | 0.414120 | -0.002596 | -1.904370 |
| C | 0.911561 | 0.868576 | -2.906120 |
| C | 0.525376 | 0.693776 | -4.239410 |
| C | -0.347842 | -0.356121 | -4.580430 |
| C | -0.838692 | -1.232390 | -3.590640 |
| C | -0.457390 | -1.065420 | -2.257480 |
| H | -1.165880 | 1.444560 | 1.264390 |
| H | -1.748850 | 2.191560 | 3.563970 |
| H | -0.830393 | 0.984259 | 5.561960 |
| H | 0.686639 | -0.984094 | 5.213210 |
| H | 1.254470 | -1.744140 | 2.916180 |
| H | 1.589730 | 1.681370 | -2.622410 |
| H | 0.902021 | 1.366660 | -5.015530 |
| H | -0.644167 | -0.496335 | -5.625130 |
| H | -1.514220 | -2.046170 | -3.871400 |
| H | -0.792204 | -1.725040 | -1.449360 |

**OBF2**

$E_{\text{XMS-SA2-CASPT2(2,2)/cc-pVDZ}}$ = -538.8405882829 a.u.

| | | | |
|---|---|---|---|
| C | -0.023476 | 0.206568 | -0.065722 |
| C | 0.007453 | -0.001534 | 1.382750 |
| C | 1.250870 | -0.253137 | 2.027410 |
| C | 1.324990 | -0.386860 | 3.420770 |
| C | 0.173034 | -0.275957 | 4.218270 |
| C | -1.060440 | -0.018455 | 3.593160 |
| C | -1.144690 | 0.124870 | 2.202110 |
| C | 0.470833 | -0.649070 | -1.089550 |
| H | -0.931618 | -0.484239 | -0.414407 |
| H | 0.755355 | -0.185033 | -2.056840 |
| C | 0.553224 | -2.114230 | -1.131100 |
| C | 1.054770 | -2.726720 | -2.304890 |
| C | 1.246340 | -4.113180 | -2.356360 |
| C | 0.930744 | -4.901460 | -1.236210 |
| C | 0.427016 | -4.301680 | -0.064861 |
| C | 0.244117 | -2.917060 | -0.001176 |
| H | 2.158110 | -0.342279 | 1.418930 |

| | | | |
|---|---|---|---|
| H | 2.297390 | -0.581545 | 3.887340 |
| H | 0.235292 | -0.381627 | 5.305550 |
| H | -1.969830 | 0.077564 | 4.196740 |
| H | -2.111290 | 0.342849 | 1.733000 |
| H | 1.293400 | -2.101930 | -3.173160 |
| H | 1.634130 | -4.580780 | -3.266450 |
| H | 1.076230 | -5.985760 | -1.273540 |
| H | 0.187456 | -4.921650 | 0.804509 |
| H | -0.131286 | -2.445420 | 0.911666 |

**HT1**

$E_{\text{XMS-SA2-CASPT2(2,2)/cc-pVDZ}}$ = -538.8521296883 a.u.

| | | | |
|---|---|---|---|
| C | 0.003171 | -0.041539 | -0.037831 |
| C | -0.039714 | -0.076606 | 1.512200 |
| C | 1.147760 | 0.245476 | 2.219550 |
| C | 1.226480 | 0.101638 | 3.614160 |
| C | 0.105072 | -0.340496 | 4.331550 |
| C | -1.100460 | -0.632205 | 3.655910 |
| C | -1.183940 | -0.463532 | 2.272400 |
| C | -0.187067 | 1.381720 | 0.191814 |
| H | -0.911854 | -0.569333 | -0.365385 |
| H | -1.069890 | 1.819760 | 0.718247 |
| C | 0.805912 | 2.387100 | -0.153496 |
| C | 0.599951 | 3.749630 | 0.179280 |
| C | 1.518810 | 4.720120 | -0.234465 |
| C | 2.653290 | 4.338540 | -0.976321 |
| C | 2.867140 | 2.985730 | -1.307890 |
| C | 1.956080 | 2.009270 | -0.893172 |
| H | 2.018240 | 0.570591 | 1.640660 |
| H | 2.158100 | 0.335756 | 4.139070 |
| H | 0.162885 | -0.462869 | 5.418060 |
| H | -1.972370 | -0.977453 | 4.221300 |
| H | -2.119750 | -0.677996 | 1.743760 |
| H | -0.287594 | 4.030470 | 0.758029 |
| H | 1.360090 | 5.772960 | 0.018938 |
| H | 3.375150 | 5.098350 | -1.293780 |
| H | 3.751810 | 2.701190 | -1.886500 |
| H | 2.068910 | 0.940917 | -1.111420 |

**HT2**

$E_{\text{XMS-SA2-CASPT2(2,2)/cc-pVDZ}}$ = -538.8395435151 a.u.

| | | | |
|---|---|---|---|
| C | 1.667020 | -0.176071 | 0.212057 |
| C | 0.505177 | -0.113924 | 1.116090 |
| C | -0.166768 | 1.100610 | 1.394010 |
| C | -1.215760 | 1.132120 | 2.319610 |
| C | -1.616030 | -0.044290 | 2.981020 |
| C | -0.954806 | -1.256280 | 2.715890 |
| C | 0.097405 | -1.288930 | 1.792060 |
| C | 2.260140 | 0.939444 | -0.604444 |
| H | 2.220090 | -1.124600 | 0.324166 |

| | | | |
|---|---|---|---|
| H | 2.141660 | 1.949100 | -0.175053 |
| C | 1.157190 | 0.578554 | -1.480710 |
| C | -0.007728 | 1.367000 | -1.808090 |
| C | -1.156150 | 0.769458 | -2.296140 |
| C | -1.250560 | -0.651418 | -2.439100 |
| C | -0.132201 | -1.441280 | -2.208540 |
| C | 1.080680 | -0.839330 | -1.776340 |
| H | 0.156237 | 2.015730 | 0.890177 |
| H | -1.724050 | 2.077790 | 2.533600 |
| H | -2.436730 | -0.014052 | 3.704730 |
| H | -1.260320 | -2.172620 | 3.230860 |
| H | 0.617225 | -2.230890 | 1.581100 |
| H | 0.039566 | 2.450900 | -1.654850 |
| H | -2.024550 | 1.388390 | -2.548520 |
| H | -2.186710 | -1.099930 | -2.785730 |
| H | -0.166544 | -2.520080 | -2.393880 |
| H | 2.015100 | -1.404710 | -1.801460 |

**$CI_{DHP}$**

$E_{\text{XMS-SA2-CASPT2(2,2)/cc-pVDZ}}$ = -538.8474208667 a.u.

| | | | |
|---|---|---|---|
| C | 0.483256 | -0.290931 | -1.442490 |
| C | 0.581029 | -1.275730 | -0.440326 |
| C | 0.583718 | -0.942933 | 0.923924 |
| C | 0.826529 | 0.517217 | 1.161710 |
| C | 0.871876 | 0.919931 | 2.578950 |
| C | 0.758795 | -0.028511 | 3.572270 |
| C | 0.428308 | -1.848810 | 2.017990 |
| C | 0.509852 | -1.424930 | 3.329230 |
| C | -0.236513 | 0.928667 | -1.167250 |
| C | -0.807073 | 1.752630 | -2.174720 |
| C | -0.459821 | 1.397260 | 0.170681 |
| C | -1.375340 | 2.993320 | -1.851820 |
| C | -1.079210 | 2.616560 | 0.506065 |
| C | -1.508100 | 3.459520 | -0.524190 |
| H | 0.545881 | -0.591957 | -2.493960 |
| H | 0.608708 | -2.337630 | -0.728175 |
| H | 1.601080 | 0.903479 | 0.455741 |
| H | 1.104060 | 1.960150 | 2.828740 |
| H | 0.885009 | 0.310030 | 4.608440 |
| H | 0.237991 | -2.902980 | 1.779090 |
| H | 0.399772 | -2.119350 | 4.165030 |
| H | -0.767228 | 1.434700 | -3.222110 |
| H | -0.707398 | 0.556975 | 0.901262 |
| H | -1.739350 | 3.623810 | -2.671710 |
| H | -1.207170 | 2.874810 | 1.564230 |
| H | -1.924890 | 4.448560 | -0.317097 |

## II.E XMS-SA3-CASPT2(2,2)/cc-pVDZ

***trans* $S_0$min**

$E_{\text{XMS-SA3-CASPT2(2,2)/cc-pVDZ}}$ = -539.0072559429 a.u.

| | | | |
|---|---|---|---|
| C | 1.973750 | -0.025217 | 0.066704 |
| C | -1.925520 | -0.049411 | -0.030002 |
| C | 0.555201 | 0.018542 | 0.446691 |
| C | -0.505510 | -0.092049 | -0.404188 |
| C | 2.947190 | -0.220363 | 1.074440 |
| C | -2.896080 | -0.433408 | -0.984981 |
| C | 2.420220 | 0.126901 | -1.270540 |
| C | -2.377320 | 0.365337 | 1.248590 |
| C | 4.312760 | -0.283368 | 0.762814 |
| C | -4.263780 | -0.422170 | -0.676432 |
| C | 3.782780 | 0.061342 | -1.582130 |
| C | -3.741680 | 0.373546 | 1.557930 |
| C | 4.738220 | -0.144874 | -0.568675 |
| C | -4.694540 | -0.020163 | 0.598558 |
| H | 0.363944 | 0.117363 | 1.523570 |
| H | -0.310723 | -0.258346 | -1.472440 |
| H | 2.620840 | -0.330770 | 2.115150 |
| H | -2.565840 | -0.749698 | -1.981320 |
| H | 1.694980 | 0.314731 | -2.068660 |
| H | -1.655130 | 0.697275 | 2.001100 |
| H | 5.046060 | -0.439417 | 1.560530 |
| H | -4.994520 | -0.726532 | -1.432550 |
| H | 4.106830 | 0.183350 | -2.620850 |
| H | -4.069400 | 0.698512 | 2.550970 |
| H | 5.803290 | -0.188891 | -0.816450 |
| H | -5.761130 | -0.006026 | 0.843497 |

***cis* $S_0$min**

$E_{\text{XMS-SA3-CASPT2(2,2)/cc-pVDZ}}$ = -539.0041064367 a.u.

| | | | |
|---|---|---|---|
| C | -4.653880 | 0.068217 | 0.453939 |
| C | -4.173280 | -0.601418 | -0.682485 |
| C | -2.796140 | -0.623169 | -0.956696 |
| C | -1.875150 | 0.003858 | -0.089505 |
| C | -2.373650 | 0.692834 | 1.043040 |
| C | -3.747370 | 0.721471 | 1.310910 |
| C | -0.432144 | -0.023662 | -0.406573 |
| C | 0.599136 | -0.171085 | 0.475405 |
| C | 0.498263 | -0.453886 | 1.922070 |
| C | 1.398910 | 0.158927 | 2.820400 |
| C | 1.330770 | -0.101244 | 4.198720 |
| C | 0.373108 | -0.997613 | 4.698850 |
| C | -0.511678 | -1.637010 | 3.809280 |
| C | -0.450213 | -1.371520 | 2.436210 |
| H | 1.618690 | -0.079786 | 0.076454 |
| H | -0.173198 | 0.097276 | -1.467420 |
| H | 2.151440 | 0.855797 | 2.433480 |
| H | -2.425470 | -1.143090 | -1.847760 |

| | | | |
|---|---|---|---|
| H | -1.130580 | -1.881400 | 1.746840 |
| H | -1.673510 | 1.213200 | 1.704230 |
| H | 2.031510 | 0.392100 | 4.880280 |
| H | -4.870670 | -1.102520 | -1.361770 |
| H | -1.247330 | -2.352820 | 4.190460 |
| H | -4.117510 | 1.262640 | 2.187880 |
| H | 0.321502 | -1.207810 | 5.771980 |
| H | -5.727390 | 0.092690 | 0.667083 |

**DHP $S_0$min**

$E_{\text{XMS-SA3-CASPT2(2,2)/cc-pVDZ}}$ = -538.9487101281 a.u.

| | | | |
|---|---|---|---|
| C | 1.437660 | -0.871259 | -0.022752 |
| C | -1.442610 | -0.895400 | -0.066691 |
| C | 0.720564 | -2.056340 | -0.019821 |
| C | -0.704383 | -2.063460 | -0.163369 |
| C | 2.871750 | -0.852759 | 0.099380 |
| C | -2.878000 | -0.894603 | -0.171795 |
| C | 0.694701 | 0.434591 | -0.299012 |
| C | -0.722541 | 0.400086 | 0.302576 |
| C | 3.557580 | 0.329811 | 0.257404 |
| C | -3.586340 | 0.283681 | -0.234424 |
| C | 1.489000 | 1.645070 | 0.133846 |
| C | -1.539800 | 1.623340 | -0.042902 |
| C | 2.834860 | 1.588170 | 0.339995 |
| C | -2.886690 | 1.557160 | -0.238449 |
| H | 1.262900 | -3.007110 | 0.057835 |
| H | -1.230250 | -3.015360 | -0.310143 |
| H | 3.403580 | -1.811730 | 0.130117 |
| H | -3.393100 | -1.858280 | -0.265786 |
| H | 0.565634 | 0.495808 | -1.410110 |
| H | -0.595205 | 0.383754 | 1.415530 |
| H | 4.646110 | 0.324751 | 0.375719 |
| H | -4.676000 | 0.267387 | -0.337916 |
| H | 0.978338 | 2.610810 | 0.202973 |
| H | -1.045500 | 2.599450 | -0.055478 |
| H | 3.384230 | 2.499810 | 0.598709 |
| H | -3.454390 | 2.473940 | -0.430532 |

***twist* $S_1$min**

$E_{\text{XMS-SA3-CASPT2(2,2)/cc-pVDZ}}$ = -538.8720251517 a.u.

| | | | |
|---|---|---|---|
| C | 1.664810 | -0.236255 | 0.209196 |
| C | -1.632080 | -0.588802 | -0.118477 |
| C | 0.493792 | -0.978171 | 0.541349 |
| C | -0.465299 | -1.453130 | -0.461244 |
| C | 2.533070 | 0.269189 | 1.217420 |
| C | -2.948280 | -1.131170 | -0.002826 |
| C | 1.964040 | -0.026390 | -1.168570 |
| C | -1.497260 | 0.816987 | 0.087030 |
| C | 3.685820 | 0.964597 | 0.853190 |
| C | -4.054340 | -0.310555 | 0.226348 |

| | | | |
|---|---|---|---|
| C | 3.120400 | 0.668718 | -1.524130 |
| C | -2.614170 | 1.628790 | 0.355548 |
| C | 3.973350 | 1.162050 | -0.514490 |
| C | -3.900930 | 1.079030 | 0.426380 |
| H | 0.331448 | -1.106270 | 1.637490 |
| H | -0.746481 | -2.495750 | -0.189143 |
| H | 2.280410 | 0.103590 | 2.270650 |
| H | -3.086530 | -2.209380 | -0.145615 |
| H | 1.236700 | -0.445693 | -1.878470 |
| H | -0.516433 | 1.291290 | -0.032585 |
| H | 4.363110 | 1.358620 | 1.616660 |
| H | -5.053300 | -0.759712 | 0.269203 |
| H | 3.369590 | 0.837561 | -2.576030 |
| H | -2.468920 | 2.705510 | 0.502416 |
| H | 4.877390 | 1.712770 | -0.796519 |
| H | -4.772710 | 1.714170 | 0.614415 |

***trans*** **$S_1$min**

$E_{\text{XMS-SA3-CASPT2(2,2)/cc-pVDZ}}$ = -538.8554270589 a.u.

| | | | |
|---|---|---|---|
| C | 1.933720 | -0.087643 | 0.115234 |
| C | -1.887300 | -0.103972 | -0.072446 |
| C | 0.570851 | -0.114994 | 0.486763 |
| C | -0.524588 | -0.213540 | -0.429123 |
| C | 2.958460 | -0.043672 | 1.127550 |
| C | -2.912250 | -0.276927 | -1.070640 |
| C | 2.368400 | -0.107808 | -1.266050 |
| C | -2.321620 | 0.174889 | 1.280660 |
| C | 4.307080 | -0.029342 | 0.791690 |
| C | -4.260710 | -0.186997 | -0.746108 |
| C | 3.725160 | -0.094698 | -1.582560 |
| C | -3.678230 | 0.259622 | 1.586530 |
| C | 4.707260 | -0.057682 | -0.568371 |
| C | -4.660540 | 0.079885 | 0.587916 |
| H | 0.348854 | -0.081420 | 1.561770 |
| H | -0.302777 | -0.412273 | -1.486170 |
| H | 2.652280 | -0.025503 | 2.181120 |
| H | -2.606350 | -0.487340 | -2.103230 |
| H | 1.623390 | -0.118535 | -2.068690 |
| H | -1.576390 | 0.335266 | 2.066990 |
| H | 5.064710 | 0.002897 | 1.582240 |
| H | -5.018520 | -0.324271 | -1.525150 |
| H | 4.033510 | -0.112775 | -2.634290 |
| H | -3.986300 | 0.469351 | 2.617380 |
| H | 5.770760 | -0.049467 | -0.831346 |
| H | -5.723930 | 0.147655 | 0.842609 |

***cis*** **$S_1$min**

$E_{\text{XMS-SA3-CASPT2(2,2)/cc-pVDZ}}$ = -538.8750335452 a.u.

| | | | |
|---|---|---|---|
| C | -4.302700 | 0.029541 | 0.561853 |
| C | -3.968380 | -0.639076 | -0.650947 |

| | | | |
|---|---|---|---|
| C | -2.632970 | -0.823061 | -1.011530 |
| C | -1.575460 | -0.376965 | -0.176147 |
| C | -1.912770 | 0.342887 | 1.074050 |
| C | -3.299280 | 0.546437 | 1.377000 |
| C | -0.208835 | -0.663878 | -0.452766 |
| C | 0.782267 | -0.543188 | 0.533690 |
| C | 0.460036 | -0.505825 | 1.918780 |
| C | 1.373230 | -0.035082 | 2.898410 |
| C | 0.991336 | 0.123170 | 4.230580 |
| C | -0.331628 | -0.212823 | 4.640660 |
| C | -1.240420 | -0.744470 | 3.729940 |
| C | -0.909528 | -0.884906 | 2.340550 |
| H | 1.840680 | -0.500268 | 0.243989 |
| H | 0.067628 | -0.958758 | -1.473710 |
| H | 2.384850 | 0.242503 | 2.577470 |
| H | -2.381230 | -1.358350 | -1.935300 |
| H | -1.349740 | -1.746350 | 1.814400 |
| H | -1.223300 | 1.152040 | 1.360000 |
| H | 1.708810 | 0.508502 | 4.962760 |
| H | -4.764510 | -1.014590 | -1.302840 |
| H | -2.225110 | -1.081620 | 4.074630 |
| H | -3.563330 | 1.134310 | 2.264160 |
| H | -0.611815 | -0.114901 | 5.695360 |
| H | -5.354830 | 0.195392 | 0.817538 |

***DHP* $\mathbf{S_1}$min**

$E_{\text{XMS-SA3-CASPT2(2,2)/cc-pVDZ}}$ = -538.8898067396 a.u.

| | | | |
|---|---|---|---|
| C | 1.445220 | -0.892054 | 0.022090 |
| C | -1.458470 | -0.913824 | -0.019259 |
| C | 0.702981 | -2.098990 | 0.097211 |
| C | -0.698542 | -2.110920 | -0.077732 |
| C | 2.773790 | -0.784299 | 0.505787 |
| C | -2.779800 | -0.824628 | -0.527265 |
| C | 0.747536 | 0.328087 | -0.492932 |
| C | -0.779731 | 0.320369 | 0.489527 |
| C | 3.423270 | 0.451312 | 0.583323 |
| C | -3.440870 | 0.403261 | -0.626553 |
| C | 1.492130 | 1.572900 | -0.413617 |
| C | -1.539260 | 1.555070 | 0.393716 |
| C | 2.760900 | 1.635940 | 0.149998 |
| C | -2.799540 | 1.598370 | -0.189911 |
| H | 1.240220 | -3.038610 | 0.281469 |
| H | -1.218360 | -3.061320 | -0.257876 |
| H | 3.271750 | -1.692760 | 0.866612 |
| H | -3.262860 | -1.740690 | -0.889860 |
| H | 0.239706 | 0.165177 | -1.460390 |
| H | -0.272690 | 0.172534 | 1.459860 |
| H | 4.440290 | 0.509489 | 0.985541 |
| H | -4.449550 | 0.448108 | -1.050850 |
| H | 1.025800 | 2.481540 | -0.814286 |
| H | -1.087860 | 2.472450 | 0.791836 |
| H | 3.280480 | 2.598860 | 0.207636 |

| | | | |
|---|---|---|---|
| H | -3.328800 | 2.554840 | -0.262629 |

**OBF1**

$E_{\text{XMS-SA3-CASPT2(2,2)/cc-pVDZ}}$ = -538.8685762148 a.u.

| | | | |
|---|---|---|---|
| C | 0.507957 | -0.863016 | 0.713000 |
| C | 0.045666 | -0.083177 | 1.894500 |
| C | -0.722109 | 1.100100 | 1.725180 |
| C | -1.229390 | 1.803070 | 2.830460 |
| C | -0.982724 | 1.358010 | 4.138680 |
| C | -0.223748 | 0.186405 | 4.320080 |
| C | 0.282336 | -0.522034 | 3.222460 |
| C | 0.996132 | -0.063091 | -0.390123 |
| H | 1.619230 | -1.010600 | 1.005360 |
| H | 1.740510 | 0.757530 | -0.253622 |
| C | 0.639966 | -0.304917 | -1.755930 |
| C | 1.188400 | 0.481226 | -2.804410 |
| C | 0.761582 | 0.279286 | -4.119130 |
| C | -0.202828 | -0.712705 | -4.387690 |
| C | -0.749177 | -1.505070 | -3.355740 |
| C | -0.326285 | -1.309800 | -2.040850 |
| H | -0.949941 | 1.450580 | 0.709223 |
| H | -1.820510 | 2.711220 | 2.662400 |
| H | -1.381250 | 1.904280 | 4.999740 |
| H | -0.022832 | -0.181954 | 5.332930 |
| H | 0.859482 | -1.440470 | 3.384240 |
| H | 1.934500 | 1.247360 | -2.564700 |
| H | 1.170770 | 0.880376 | -4.936190 |
| H | -0.531748 | -0.873533 | -5.419990 |
| H | -1.494450 | -2.269970 | -3.593070 |
| H | -0.682625 | -1.888160 | -1.177960 |

**OBF2**

$E_{\text{XMS-SA3-CASPT2(2,2)/cc-pVDZ}}$ = -538.8597541304 a.u.

| | | | |
|---|---|---|---|
| C | -0.030119 | 0.236082 | -0.076821 |
| C | 0.007774 | -0.002466 | 1.373520 |
| C | 1.246490 | -0.286700 | 2.015210 |
| C | 1.319640 | -0.446532 | 3.406310 |
| C | 0.171599 | -0.336117 | 4.210280 |
| C | -1.056650 | -0.049016 | 3.588310 |
| C | -1.141010 | 0.121236 | 2.199180 |
| C | 0.557603 | -0.635501 | -1.066750 |
| H | -0.974223 | -0.376778 | -0.404344 |
| H | 0.965317 | -0.164130 | -1.986580 |
| C | 0.585014 | -2.084740 | -1.127260 |
| C | 1.149280 | -2.708810 | -2.268920 |
| C | 1.293250 | -4.100370 | -2.322610 |
| C | 0.871027 | -4.879590 | -1.232060 |
| C | 0.308039 | -4.273960 | -0.088625 |
| C | 0.168646 | -2.886950 | -0.026393 |
| H | 2.156150 | -0.374004 | 1.407850 |

| | | | |
|---|---|---|---|
| H | 2.291190 | -0.662375 | 3.866570 |
| H | 0.233942 | -0.461471 | 5.295870 |
| H | -1.965840 | 0.048024 | 4.193030 |
| H | -2.106810 | 0.361449 | 1.737650 |
| H | 1.471120 | -2.084330 | -3.110460 |
| H | 1.726000 | -4.578590 | -3.206280 |
| H | 0.980155 | -5.968370 | -1.269010 |
| H | -0.008690 | -4.893690 | 0.755531 |
| H | -0.249682 | -2.402330 | 0.861059 |

**HT1**

$E_{\text{XMS-SA3-CASPT2(2,2)/cc-pVDZ}}$ = -538.8723053197 a.u.

| | | | |
|---|---|---|---|
| C | 0.043330 | 0.040960 | 0.021368 |
| C | -0.047522 | -0.091593 | 1.532720 |
| C | 0.926071 | 0.518443 | 2.370750 |
| C | 0.885613 | 0.366696 | 3.768690 |
| C | -0.129199 | -0.388697 | 4.369980 |
| C | -1.114350 | -0.991437 | 3.557660 |
| C | -1.104910 | -0.806240 | 2.172930 |
| C | -0.159534 | 1.491620 | 0.130247 |
| H | -0.874277 | -0.435110 | -0.386554 |
| H | -1.090710 | 1.943280 | 0.545096 |
| C | 0.860299 | 2.451930 | -0.195607 |
| C | 0.655547 | 3.842400 | 0.006446 |
| C | 1.635690 | 4.755950 | -0.389963 |
| C | 2.826380 | 4.287200 | -0.981322 |
| C | 3.042620 | 2.908740 | -1.183080 |
| C | 2.067770 | 1.989730 | -0.787608 |
| H | 1.756320 | 1.064350 | 1.910300 |
| H | 1.658050 | 0.838683 | 4.385720 |
| H | -0.153201 | -0.521535 | 5.456430 |
| H | -1.908850 | -1.589520 | 4.017930 |
| H | -1.882420 | -1.266280 | 1.552430 |
| H | -0.276713 | 4.182940 | 0.471430 |
| H | 1.485590 | 5.829460 | -0.240566 |
| H | 3.595840 | 5.005410 | -1.284180 |
| H | 3.974080 | 2.565050 | -1.643530 |
| H | 2.158130 | 0.900515 | -0.900716 |

**HT2**

$E_{\text{XMS-SA3-CASPT2(2,2)/cc-pVDZ}}$ = -538.8590928258 a.u.

| | | | |
|---|---|---|---|
| C | 1.675200 | -0.176918 | 0.213071 |
| C | 0.585210 | -0.125517 | 1.185620 |
| C | -0.197592 | 1.040980 | 1.381130 |
| C | -1.153230 | 1.085320 | 2.398610 |
| C | -1.344560 | -0.027646 | 3.242840 |
| C | -0.574154 | -1.188120 | 3.063720 |
| C | 0.380807 | -1.236650 | 2.039400 |
| C | 2.126280 | 0.896518 | -0.727050 |
| H | 2.252690 | -1.116900 | 0.290847 |

| | | | |
|---|---|---|---|
| H | 2.042920 | 1.922360 | -0.325423 |
| C | 0.997801 | 0.521283 | -1.565090 |
| C | -0.107158 | 1.362800 | -1.950540 |
| C | -1.252060 | 0.821151 | -2.512350 |
| C | -1.412630 | -0.591360 | -2.658540 |
| C | -0.349856 | -1.431830 | -2.341920 |
| C | 0.862768 | -0.892430 | -1.847200 |
| H | -0.039310 | 1.901540 | 0.725766 |
| H | -1.753660 | 1.988820 | 2.544490 |
| H | -2.093350 | 0.014676 | 4.040280 |
| H | -0.720418 | -2.052120 | 3.719400 |
| H | 0.985082 | -2.139150 | 1.889920 |
| H | -0.015456 | 2.445820 | -1.804310 |
| H | -2.065620 | 1.485760 | -2.825530 |
| H | -2.344060 | -0.995905 | -3.067090 |
| H | -0.429597 | -2.510710 | -2.515870 |
| H | 1.756390 | -1.520730 | -1.793380 |

**$CI_{DHP}$**

$E_{\text{XMS-SA3-CASPT2(2,2)/cc-pVDZ}}$ = -538.8748704170 a.u.

| | | | |
|---|---|---|---|
| C | 0.480414 | -0.285877 | -1.431930 |
| C | 0.581336 | -1.284610 | -0.427436 |
| C | 0.666487 | -0.913701 | 0.919369 |
| C | 0.863153 | 0.545599 | 1.096320 |
| C | 1.048370 | 0.977143 | 2.468520 |
| C | 0.917523 | 0.069958 | 3.510580 |
| C | 0.445061 | -1.775340 | 2.034570 |
| C | 0.560545 | -1.304620 | 3.333300 |
| C | -0.378340 | 0.828664 | -1.130170 |
| C | -0.979692 | 1.637130 | -2.129110 |
| C | -0.666866 | 1.258260 | 0.243789 |
| C | -1.355040 | 2.955630 | -1.817590 |
| C | -1.176810 | 2.555620 | 0.525384 |
| C | -1.408940 | 3.471570 | -0.501743 |
| H | 0.540153 | -0.580855 | -2.486870 |
| H | 0.556187 | -2.355810 | -0.688879 |
| H | 1.510810 | 0.944867 | 0.279169 |
| H | 1.334540 | 2.013960 | 2.677160 |
| H | 1.130770 | 0.429297 | 4.525690 |
| H | 0.191596 | -2.824420 | 1.833910 |
| H | 0.432442 | -1.961930 | 4.197370 |
| H | -0.989694 | 1.307500 | -3.173560 |
| H | -1.105020 | 0.479782 | 0.894798 |
| H | -1.618870 | 3.617330 | -2.653380 |
| H | -1.388410 | 2.804020 | 1.573680 |
| H | -1.677390 | 4.513840 | -0.312631 |

## II.F OM3/MRCI(12,17)

***trans* S$_0$min**

$E_{\mathrm{OM3/MRCI(12,17)}}$ = -1928.911314 eV

| | | | |
|---|---|---|---|
| C | 1.94944405 | 0.12230450 | 0.18531263 |
| C | -1.89802434 | 0.12687601 | -0.10133340 |
| C | 0.51090141 | 0.12809383 | 0.53373022 |
| C | -0.45329859 | 0.12880708 | -0.42071068 |
| C | 2.81518843 | -0.78326284 | 0.82560517 |
| C | -2.75395463 | -0.75107262 | -0.79198523 |
| C | 2.44944287 | 1.01388884 | -0.78101113 |
| C | -2.41785491 | 0.99658124 | 0.87412789 |
| C | 4.17404655 | -0.79946739 | 0.49520863 |
| C | -4.12246464 | -0.76070664 | -0.50444644 |
| C | 3.80887362 | 0.98699627 | -1.11170095 |
| C | -3.78815722 | 0.97978364 | 1.15835288 |
| C | 4.66716124 | 0.08097858 | -0.47736962 |
| C | -4.63716442 | 0.102628 | 0.47216556 |
| H | 0.26268774 | 0.11498014 | 1.60831470 |
| H | -0.19390368 | 0.11354770 | -1.49348150 |
| H | 2.42177289 | -1.48447970 | 1.57749147 |
| H | -2.34468494 | -1.43643711 | -1.55017880 |
| H | 1.77699454 | 1.73508746 | -1.26959677 |
| H | -1.75323095 | 1.69503008 | 1.40461638 |
| H | 4.85266839 | -1.50912312 | 0.99339555 |
| H | -4.79174626 | -1.45091735 | -1.04015128 |
| H | 4.20148440 | 1.68318854 | -1.86942367 |
| H | -4.19660342 | 1.66035526 | 1.92135330 |
| H | 5.73671055 | 0.06001209 | -0.74321537 |
| H | -5.71525241 | 0.08995944 | 0.70153920 |

***cis* S$_0$min**

$E_{\mathrm{OM3/MRCI(12,17)}}$ = -1928.861473 eV

| | | | |
|---|---|---|---|
| C | -4.61406481 | 0.04497212 | 0.72367563 |
| C | -4.18693701 | -0.76019592 | -0.34114894 |
| C | -2.83841929 | -0.75856691 | -0.71333035 |
| C | -1.91655209 | 0.04042644 | -0.01321686 |
| C | -2.34719786 | 0.85093488 | 1.05235826 |
| C | -3.69884563 | 0.84547203 | 1.41712087 |
| C | -0.49755921 | 0.03759162 | -0.43870942 |
| C | 0.56108961 | -0.17626021 | 0.38385906 |
| C | 0.44795799 | -0.48026116 | 1.82821465 |
| C | 1.21889128 | 0.25369418 | 2.74719005 |
| C | 1.12095393 | -0.01923844 | 4.11589890 |
| C | 0.26123356 | -1.03189641 | 4.56336026 |
| C | -0.49916776 | -1.76960867 | 3.64848320 |
| C | -0.40863060 | -1.50111726 | 2.27777069 |
| H | 1.59494020 | -0.12954497 | -0.00720639 |
| H | -0.33734928 | 0.22941427 | -1.51683045 |
| H | 1.88840853 | 1.05409042 | 2.39421956 |
| H | -2.49946817 | -1.39443983 | -1.54662425 |

| | | | |
|---|---|---|---|
| H | -1.00019812 | -2.08354056 | 1.55769275 |
| H | -1.62769214 | 1.48283925 | 1.59183959 |
| H | 1.71040371 | 0.56574257 | 4.83883221 |
| H | -4.90742350 | -1.39699686 | -0.87740871 |
| H | -1.16327011 | -2.57389452 | 4.00320762 |
| H | -4.04150222 | 1.48537248 | 2.24605689 |
| H | 0.18768889 | -1.24758668 | 5.64193864 |
| H | -5.67792532 | 0.04693605 | 1.01241062 |

**DHP $S_0$min**

$E_{\mathrm{OM3/MRCI(12,17)}}$ = -1926.906282 eV

| | | | |
|---|---|---|---|
| C | 1.42629791 | -0.87205672 | 0.02239468 |
| C | -1.44189942 | -0.87292490 | -0.05774041 |
| C | 0.71248906 | -2.05209794 | 0.03187816 |
| C | -0.72665131 | -2.05082409 | -0.10976334 |
| C | 2.85809966 | -0.878594 | 0.22717966 |
| C | -2.87692402 | -0.88059260 | -0.25086510 |
| C | 0.71788909 | 0.44008909 | -0.25197440 |
| C | -0.74139491 | 0.43460958 | 0.24699865 |
| C | 3.53866047 | 0.27760995 | 0.44391968 |
| C | -3.55921503 | 0.27713013 | -0.46023477 |
| C | 1.48470822 | 1.60797101 | 0.30472523 |
| C | -1.51579682 | 1.61532950 | -0.27618281 |
| C | 2.82240628 | 1.53255533 | 0.57306034 |
| C | -2.84835489 | 1.53973340 | -0.56063440 |
| H | 1.22425074 | -3.01447027 | 0.17551121 |
| H | -1.23792177 | -3.01011098 | -0.27691280 |
| H | 3.37371584 | -1.85291136 | 0.25940299 |
| H | -3.38775655 | -1.85786664 | -0.28932149 |
| H | 0.72349057 | 0.55817541 | -1.38172676 |
| H | -0.74477207 | 0.51850522 | 1.37909617 |
| H | 4.62965610 | 0.28600511 | 0.57813672 |
| H | -4.64838571 | 0.28441683 | -0.62267830 |
| H | 0.94700724 | 2.56265128 | 0.44808674 |
| H | -0.97776150 | 2.57395510 | -0.38321998 |
| H | 3.38025435 | 2.41174118 | 0.93230813 |
| H | -3.40807304 | 2.42105280 | -0.91202714 |

***twist* $S_1$min**

$E_{\mathrm{OM3/MRCI(12,17)}}$ = -1925.268025 eV

| | | | |
|---|---|---|---|
| C | 1.80763486 | -0.29309313 | 0.33736990 |
| C | -1.69799395 | -0.45341064 | -0.14007252 |
| C | 0.58504414 | -1.00408616 | 0.68002735 |
| C | -0.44330860 | -1.14542040 | -0.23031437 |
| C | 2.90519000 | -0.20270229 | 1.22610030 |
| C | -2.80600606 | -0.85586104 | -0.93285799 |
| C | 1.86006119 | 0.31747202 | -0.93703963 |
| C | -1.84738311 | 0.69332141 | 0.68845369 |
| C | 4.04857960 | 0.48728688 | 0.83318917 |
| C | -3.99743364 | -0.13601790 | -0.90289884 |

| | | | |
|---|---|---|---|
| C | 3.00871343 | 1.01618238 | -1.31309685 |
| C | -3.05137977 | 1.39624132 | 0.70841792 |
| C | 4.09639190 | 1.09485810 | -0.43430324 |
| C | -4.13398426 | 0.99392876 | -0.08298578 |
| H | 0.61275334 | -1.44216995 | 1.77065814 |
| H | -0.45786148 | -2.10364853 | -0.72735365 |
| H | 2.84371012 | -0.68604993 | 2.21655580 |
| H | -2.71003983 | -1.74481708 | -1.57778487 |
| H | 0.96611013 | 0.21831488 | -1.57441591 |
| H | -1.01100230 | 1.02977435 | 1.32608582 |
| H | 4.91311970 | 0.55911705 | 1.50538368 |
| H | -4.84972821 | -0.45701490 | -1.53083485 |
| H | 3.05604039 | 1.50897688 | -2.29057769 |
| H | -3.15492260 | 2.28577340 | 1.36022479 |
| H | 5.00647374 | 1.64136300 | -0.73791077 |
| H | -5.08162725 | 1.55164254 | -0.06025087 |

### ***DHP*** $S_1$min

$E_{\mathrm{OM3/MRCI(12,17)}}$ = -1924.918398 eV

| | | | |
|---|---|---|---|
| C | 1.44619555 | -0.84371939 | -0.07856180 |
| C | -1.45744448 | -0.84307966 | 0.03892250 |
| C | 0.67388697 | -2.02805108 | -0.03350819 |
| C | -0.72174684 | -2.06062553 | -0.01445456 |
| C | 2.79537688 | -0.88777488 | 0.28937506 |
| C | -2.83945235 | -0.88508815 | -0.32874490 |
| C | 0.81861995 | 0.45891647 | -0.37176817 |
| C | -0.80222563 | 0.44393918 | 0.37098993 |
| C | 3.53224130 | 0.26761376 | 0.56737107 |
| C | -3.53708657 | 0.27120571 | -0.63153671 |
| C | 1.54445540 | 1.63306349 | 0.09774981 |
| C | -1.57444536 | 1.58949662 | -0.10128993 |
| C | 2.83459633 | 1.51803552 | 0.58859286 |
| C | -2.86578519 | 1.52337460 | -0.58624082 |
| H | 1.22939384 | -2.99893171 | -0.02168094 |
| H | -1.24752023 | -3.02187687 | -0.16170428 |
| H | 3.29021766 | -1.87214951 | 0.39648755 |
| H | -3.30251089 | -1.86862092 | -0.41524488 |
| H | 0.42987267 | 0.56541879 | -1.45241581 |
| H | -0.45758837 | 0.53321754 | 1.42961934 |
| H | 4.59595877 | 0.22085869 | 0.81227376 |
| H | -4.58700534 | 0.22746169 | -0.95539030 |
| H | 1.09742602 | 2.61930773 | -0.00894255 |
| H | -1.10392763 | 2.57283905 | 0.00620934 |
| H | 3.37215363 | 2.41259411 | 0.95791548 |
| H | -3.39075807 | 2.42255570 | -0.91681084 |

### **OBF1**

$E_{\mathrm{OM3/MRCI(12,17)}}$ = -1925.106234 eV

| | | | |
|---|---|---|---|
| C | 0.39034988 | -0.54817453 | 0.52464659 |
| C | 0.00021211 | -0.13325806 | 1.86830468 |

| | | | |
|---|---|---|---|
| C | -0.99498946 | 0.86804608 | 2.00422886 |
| C | -1.46015379 | 1.20797641 | 3.27475104 |
| C | -0.97083332 | 0.57521958 | 4.42106203 |
| C | 0.00453452 | -0.42872889 | 4.29514881 |
| C | 0.47929439 | -0.77369188 | 3.02914370 |
| C | 0.80882389 | 0.31742071 | -0.51233688 |
| H | 1.54650442 | -0.85840970 | 0.53767483 |
| H | 1.58735637 | 1.16023503 | -0.45364558 |
| C | 0.36680595 | 0.01163638 | -1.87235815 |
| C | 0.72041232 | 0.79608926 | -2.99198793 |
| C | 0.22088573 | 0.45604101 | -4.24942006 |
| C | -0.60649221 | -0.67177448 | -4.39179951 |
| C | -0.95643608 | -1.45210524 | -3.28044153 |
| C | -0.48000268 | -1.11133130 | -2.01372770 |
| H | -1.39269435 | 1.35993868 | 1.10420352 |
| H | -2.22311411 | 2.00281819 | 3.37494566 |
| H | -1.33408078 | 0.85214578 | 5.42225822 |
| H | 0.37879196 | -0.93900850 | 5.19625226 |
| H | 1.23855440 | -1.56940316 | 2.93282588 |
| H | 1.36902003 | 1.67913969 | -2.86375586 |
| H | 0.47402198 | 1.06550395 | -5.12899609 |
| H | -0.98766593 | -0.94188109 | -5.39189233 |
| H | -1.61502659 | -2.32379340 | -3.40296654 |
| H | -0.71012501 | -1.65943111 | -1.08298944 |

**OBF2**

$E_{\mathrm{OM3/MRCI(12,17)}}$ = -1924.972499 eV

| | | | |
|---|---|---|---|
| C | 0.10203884 | 0.06626163 | 0.03478813 |
| C | 0.08440124 | -0.06899550 | 1.47165544 |
| C | 1.32153895 | -0.16115828 | 2.15997135 |
| C | 1.33231088 | -0.18375491 | 3.55549132 |
| C | 0.14547098 | -0.10129701 | 4.29376232 |
| C | -1.08065077 | 0.00973823 | 3.62048516 |
| C | -1.10728210 | 0.02794402 | 2.22556948 |
| C | 0.64672033 | -0.72283805 | -0.98293062 |
| H | -0.90722865 | -0.38482332 | -0.50479885 |
| H | 1.07657265 | -0.18198757 | -1.87823244 |
| C | 0.62665394 | -2.19425354 | -1.13976722 |
| C | 1.02887792 | -2.77133181 | -2.36518609 |
| C | 1.09184466 | -4.15878804 | -2.49872964 |
| C | 0.76350105 | -4.97907312 | -1.41191640 |
| C | 0.37109417 | -4.41270192 | -0.19003052 |
| C | 0.29999477 | -3.02448711 | -0.05043587 |
| H | 2.25569351 | -0.20710024 | 1.58192183 |
| H | 2.29626253 | -0.26455454 | 4.08377640 |
| H | 0.16199139 | -0.11823640 | 5.39359970 |
| H | -2.01457133 | 0.09776368 | 4.19860120 |
| H | -2.06793442 | 0.13163858 | 1.69624617 |
| H | 1.29310075 | -2.12058002 | -3.21489916 |
| H | 1.39765575 | -4.60943183 | -3.45538627 |
| H | 0.82234875 | -6.07554766 | -1.51548087 |
| H | 0.11945247 | -5.05851828 | 0.66171880 |

| | | | |
|---|---|---|---|
| H | -0.01097448 | -2.57912657 | 0.90774428 |

**HT1**

$E_{\text{OM3/MRCI(12,17)}}$ = -1925.099109 eV

| | | | |
|---|---|---|---|
| C | 0.16023639 | 0.01702320 | 0.02256146 |
| C | 0.06149426 | -0.05129926 | 1.55971139 |
| C | 1.23541473 | 0.20369845 | 2.29641876 |
| C | 1.27999534 | -0.09476761 | 3.66269817 |
| C | 0.15415562 | -0.63730237 | 4.29773874 |
| C | -1.01929608 | -0.87944271 | 3.57088348 |
| C | -1.08189249 | -0.57124960 | 2.20849881 |
| C | -0.13943849 | 1.40199020 | 0.14392871 |
| H | -0.59664852 | -0.66543522 | -0.41644805 |
| H | -1.08564539 | 1.88964989 | 0.58109450 |
| C | 0.92527002 | 2.36082123 | -0.15175600 |
| C | 0.79789808 | 3.74306575 | 0.09414271 |
| C | 1.84142937 | 4.60148256 | -0.25563544 |
| C | 2.99605597 | 4.08207811 | -0.86483932 |
| C | 3.11831954 | 2.70935140 | -1.11570940 |
| C | 2.08799301 | 1.83700973 | -0.75842422 |
| H | 2.12417978 | 0.61052524 | 1.78671292 |
| H | 2.20478129 | 0.08270667 | 4.23716296 |
| H | 0.19623297 | -0.87557835 | 5.37342751 |
| H | -1.90194628 | -1.30826398 | 4.07721177 |
| H | -2.00244846 | -0.76380990 | 1.63234507 |
| H | -0.11931612 | 4.13602256 | 0.56468975 |
| H | 1.76551684 | 5.67905495 | -0.05260530 |
| H | 3.81043636 | 4.76863762 | -1.15412828 |
| H | 4.01955275 | 2.31808841 | -1.60252237 |
| H | 2.10375568 | 0.74662185 | -0.92260559 |

**HT2**

$E_{\text{OM3/MRCI(12,17)}}$ = -1924.872150 eV

| | | | |
|---|---|---|---|
| C | 1.77966425 | 0.00698742 | 0.33969978 |
| C | 0.65613898 | 0.04405333 | 1.30783970 |
| C | 0.10047650 | 1.28376121 | 1.68155520 |
| C | -0.89513259 | 1.33737885 | 2.66085897 |
| C | -1.35022178 | 0.15632796 | 3.26260129 |
| C | -0.79995685 | -1.08122212 | 2.90067264 |
| C | 0.20750844 | -1.13689046 | 1.93461019 |
| C | 2.05940930 | 0.82360330 | -0.77442456 |
| H | 2.42969713 | -0.91447446 | 0.48761002 |
| H | 2.00497392 | 1.92712221 | -0.75849019 |
| C | 0.76210544 | 0.21469529 | -1.35106973 |
| C | -0.41017389 | 0.98614440 | -1.52573637 |
| C | -1.42501718 | 0.52781003 | -2.36806090 |
| C | -1.31024981 | -0.71649024 | -3.00303800 |
| C | -0.15867898 | -1.49374289 | -2.82224925 |
| C | 0.87858123 | -1.03083013 | -2.00732991 |
| H | 0.47500890 | 2.21047099 | 1.21754396 |

| | | | |
|---|---|---|---|
| H | -1.31819135 | 2.30731006 | 2.96414197 |
| H | -2.14061666 | 0.20062967 | 4.03022743 |
| H | -1.16524923 | -2.00508638 | 3.37557312 |
| H | 0.63789101 | -2.10776958 | 1.63625081 |
| H | -0.50727824 | 1.96045177 | -1.01936602 |
| H | -2.32714421 | 1.14206443 | -2.52483191 |
| H | -2.12427866 | -1.08232910 | -3.64927027 |
| H | -0.06449678 | -2.46272515 | -3.33798531 |
| H | 1.80313534 | -1.61899177 | -1.89727303 |

**$\mathbf{CI_{DHP}}$**

$E_{\mathrm{OM3/MRCI(12,17)}}$ = -1925.016417 eV

| | | | |
|---|---|---|---|
| C | 1.40927836 | -0.90663484 | 0.17033077 |
| C | -1.41128829 | -0.86908272 | -0.08714463 |
| C | 0.67925431 | -2.09044680 | 0.06941941 |
| C | -0.67857711 | -2.06829105 | -0.25914350 |
| C | 2.72673171 | -0.80780215 | 0.70340219 |
| C | -2.81501973 | -0.83811933 | -0.41906561 |
| C | 0.78360136 | 0.25917241 | -0.47520772 |
| C | -0.79724595 | 0.37960360 | 0.37252824 |
| C | 3.42288621 | 0.37180729 | 0.68873588 |
| C | -3.47811912 | 0.37266826 | -0.47974749 |
| C | 1.47004510 | 1.53390536 | -0.29093561 |
| C | -1.58608160 | 1.55732171 | 0.37279001 |
| C | 2.77228346 | 1.52030312 | 0.14938618 |
| C | -2.86125189 | 1.61002862 | -0.17489336 |
| H | 1.18163279 | -3.08142733 | 0.26187868 |
| H | -1.19673018 | -2.96386549 | -0.55123496 |
| H | 3.14268255 | -1.71055217 | 1.20081259 |
| H | -3.35973661 | -1.77958436 | -0.51475431 |
| H | 0.31483005 | 0.04175857 | -1.52989479 |
| H | -0.23295712 | 0.30862541 | 1.31151389 |
| H | 4.43238232 | 0.46207029 | 1.08908922 |
| H | -4.55508798 | 0.38094610 | -0.76481999 |
| H | 1.03132401 | 2.44072691 | -0.71314919 |
| H | -1.14122115 | 2.47415699 | 0.81754375 |
| H | 3.36827180 | 2.45745623 | 0.07068703 |
| H | -3.37018901 | 2.55110362 | -0.37715794 |

# III. Conservation of the total energy in nonadiabatic dynamics

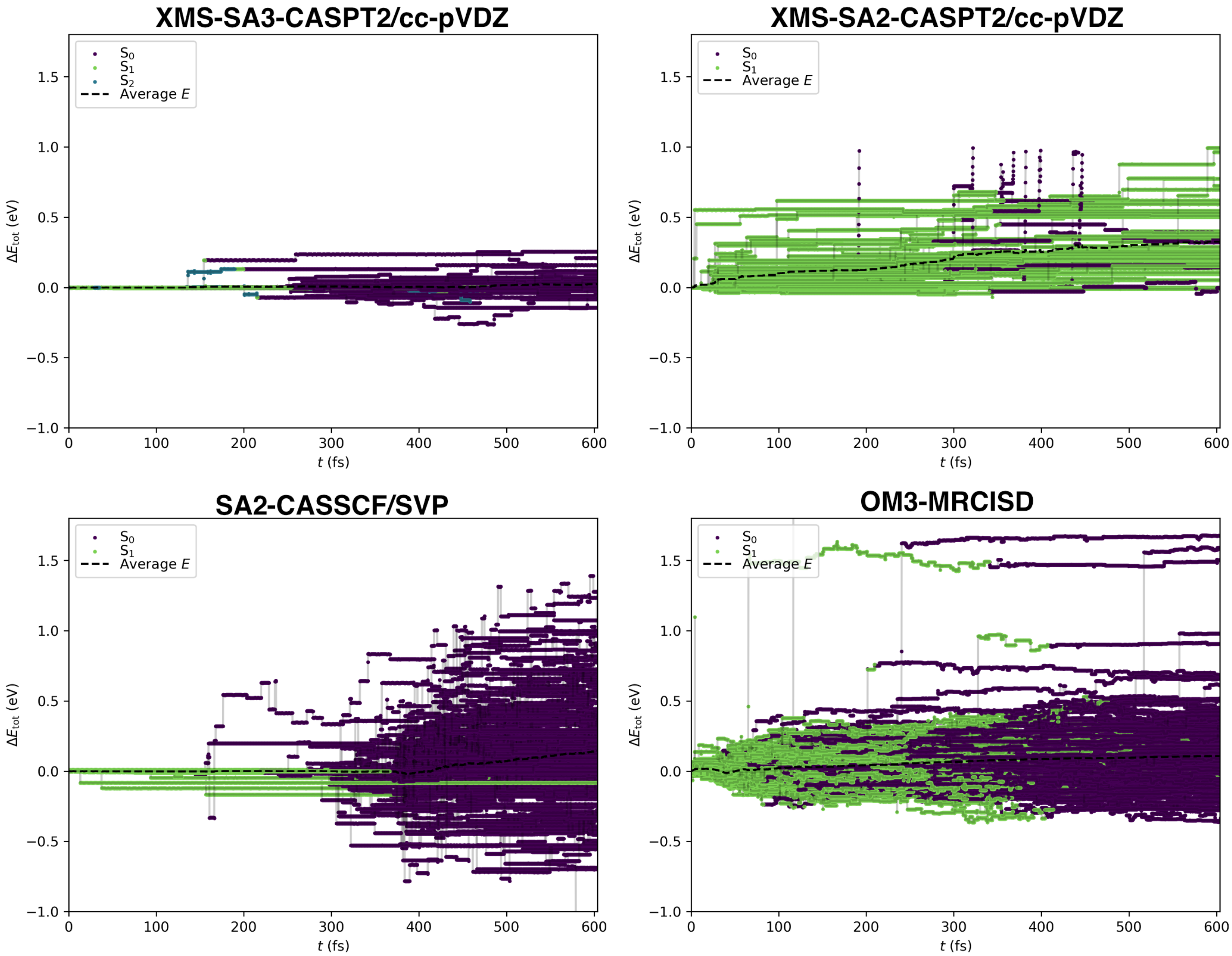


**Figure S1.** Analysis of the total energy conservation in the FSSH dynamics with XMS-SA3-CASPT2/cc-pVDZ, XMS-SA2-CASPT2/cc-pVDZ, SA2-CASSCF/SVP and OM3-MRCISD presented in the article. Plotted is the deviation of the total energy from the value at the beginning of the simulations: $\Delta E_{\mathrm{tot}}(t) = E_{\mathrm{tot}}(t) - E_{\mathrm{tot}}(0)$. The violet dots represent $\Delta E_{\mathrm{tot}}$ when the trajectory is in $S_0$, green corresponds to $S_1$ and blue to $S_2$. The dashed black line is the average $\Delta E_{\mathrm{tot}}$.

# IV. Analysis of the simulations using the SVP basis set

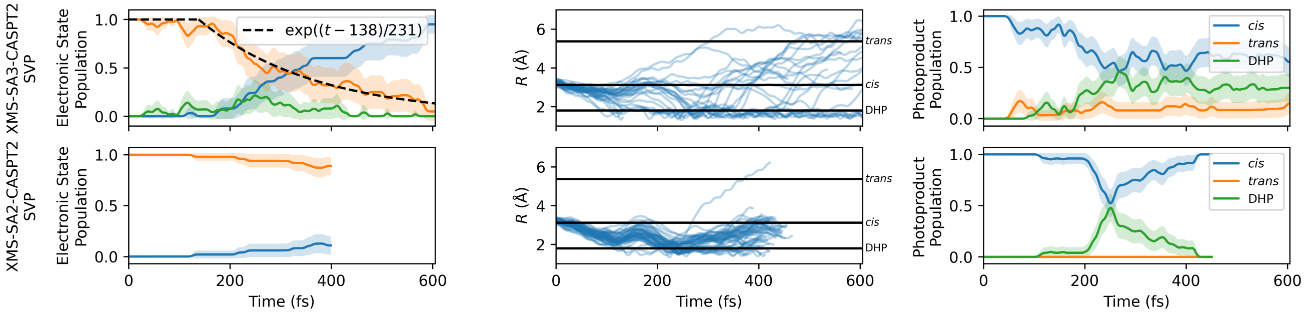


**Figure S2.** Analysis of nonadiabatic simulations for *cis*-stilbene for the XMS-CASPT2 methods using SVP basis set. We run 44 XMS-SA3-CASPT2/SVP and 51 XMS-SA2-CASPT2/SVP trajectories. The left column shows the time evolution of ground and excited-state populations upon the excitation, with accompanying 95% confidence intervals and a least squares fit for the excited-state curve. The middle column tracks the distance $R$ between $C_2$ and $C_{2'}$ atoms throughout the simulation period. We delineate three distinctive distances: the upper one, at 5.375 Å, corresponds to the *trans*-stilbene structure; the middle distance, at 3.125 Å, signifies the *cis*-stilbene configuration; and the distance at 1.8 Å represents the DHP state. The right column showcases the *cis*, *trans*, and DHP quantum yields during the dynamics. The *trans*-stilbene is defined as a conformation $90° < \theta < 270°$, where $\theta$ is the dihedral angle between atoms $C_1$, $C_0$, $C_{0'}$ and $C_{1'}$. DHP is a conformation with $R < 1.8$ Å, where $R$ is the distance between $C_2$ and $C_{2'}$. Any remaining configurations are classified as *cis*-stilbene.

**Table S2.** Calculated quantum yields $\phi$ and excited-state lifetimes $\tau$ for XMS-CASPT2 methods with SVP basis.

| Level of Theory | $\phi_{cis}$ | $\phi_{trans}$ | $\phi_{\mathrm{DHP}}$ | $\tau$ (fs) |
|---|---|---|---|---|
| XMS-SA3-CASPT2/SVP | $0.55 \pm 0.15$ | $0.15 \pm 0.11$ | $0.3 \pm 0.11$ | $369 \pm 12$ |
| XMS-SA2-CASPT2/SVP | – | – | – | – |

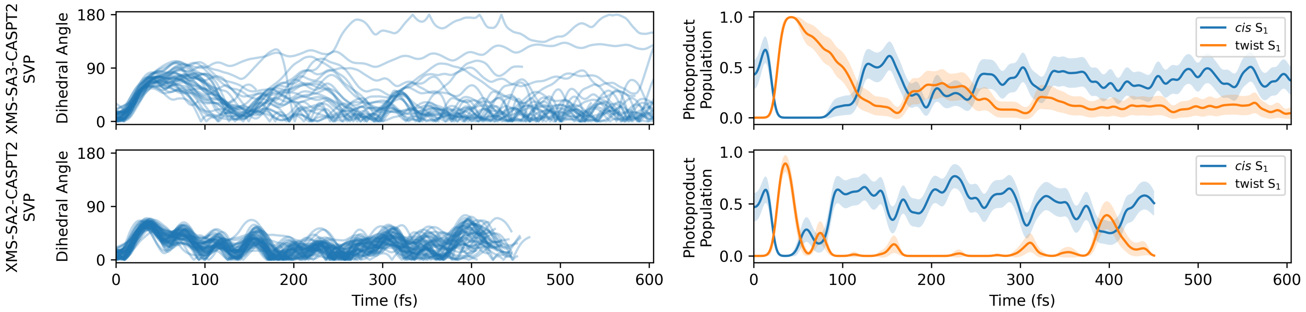


**Figure S3.** Analysis of nonadiabatic simulations for *cis*-stilbene for the XMS-CASPT2 methods using SVP basis set. The left column tracks the dihedral angle $\theta$ between $C_1$, $C_0$, $C_{0'}$ and $C_{1'}$ atoms throughout the simulation period. The right column showcases the ratios of *cis* and twist geometries during the simulation period. Twist conformation is the defined as a conformation with $50° < \theta < 90°$, while the *cis* $S_1$min has $10° < \theta < 30°$.

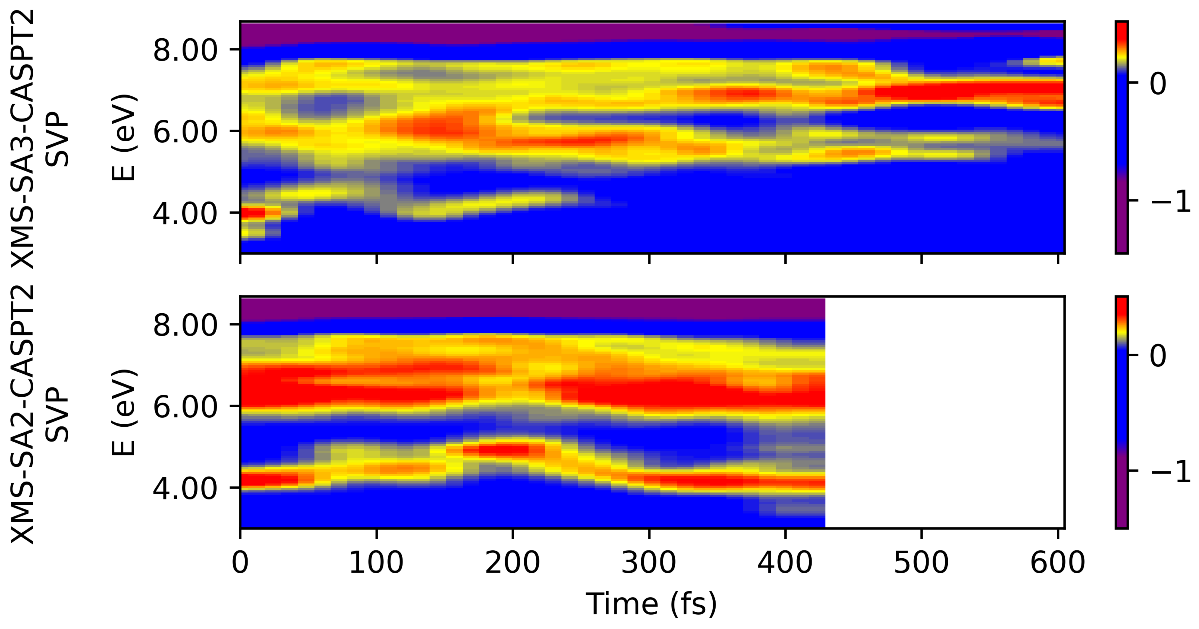


**Figure S4.** Simulated photoelectron spectra for surface hopping dynamics for the XMS-CASPT2 methods using SVP basis set; the intensities were included via Dyson norms. The pump-probe signal was calculated by subtracting the ground state signal at $t = 0$.

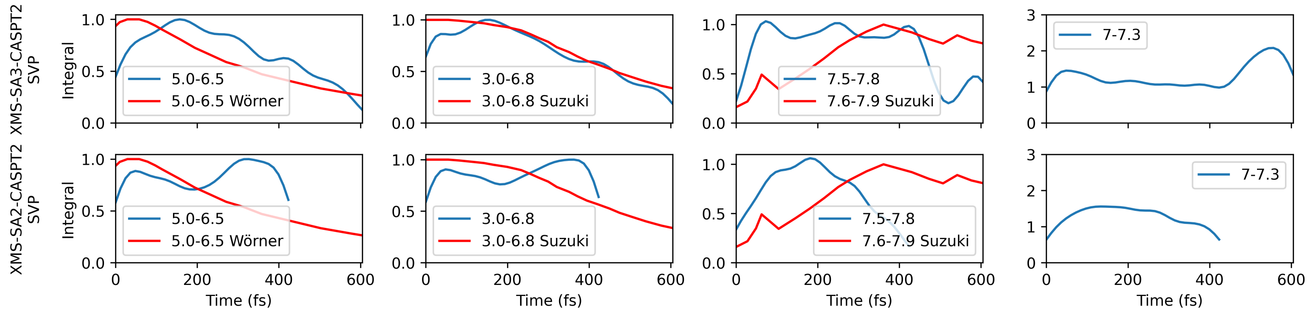


**Figure S5.** Integrated time-resolved photoelectron spectra for the XMS-CASPT2 methods using SVP basis set. The first column presents the integrals of the calculated spectra from Figure S4 in the range 5 eV to 6.5 eV (blue line) along with the experiment (red line). The second column displays the integrated calculated spectrum (blue line) from 3 eV to 6.8 eV along with the corresponding experimental spectrum (red line). The third column is analogous to the second, with the energy range from 7.5 eV to 7.9 eV. The fourth column corresponds to the 7.0 eV to 7.3 eV that should be most sensitive to the DHP formation. All the integrals were convoluted with Gaussian function with a standard deviation of 30 fs. The comparisons with the experimental data are normalized to the maximum value.

## V. Additional plots for the biased simulations

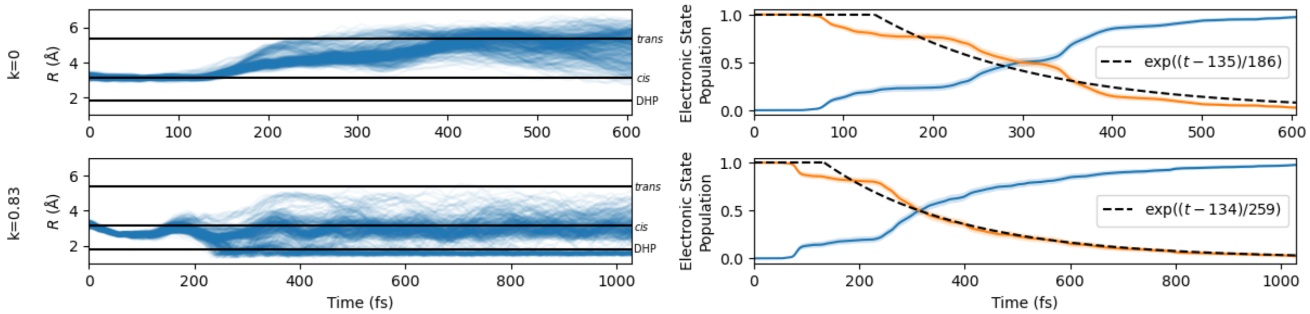


**Figure S6.** Atom distances and excited state lifetimes for the simulations with a bias. The left panel displays the distance $R$ for both the biased and unbiased simulations at the OM3-MRCISD level. On the right, the state populations are shown along with 95% confidence intervals and the least squares fit of the excited state curve.

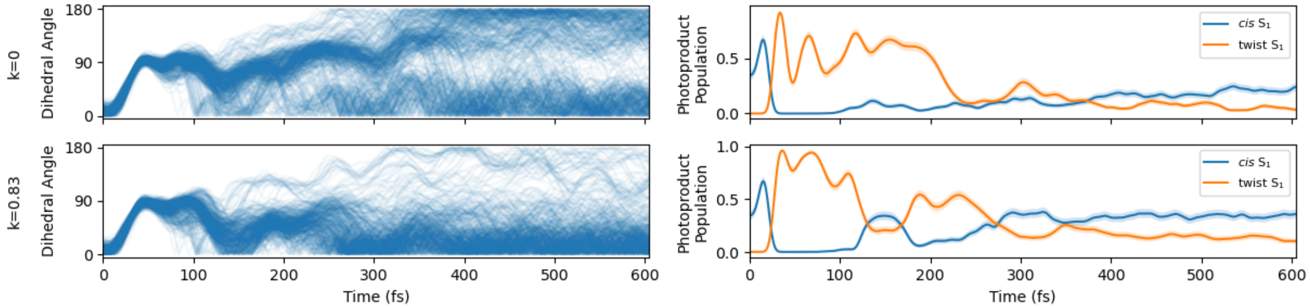


**Figure S7.** Analysis of non-adiabatic simulations with a bias for *cis*-stilbene. The left column tracks the dihedral angle $\theta$ between $C_1$, $C_0$, $C_{0'}$ and $C_{1'}$ atoms throughout the simulation period. The right column showcases the ratios of *cis* and twist geometries during the simulation period. Twist conformation is the defined as a conformation with $50^\circ < \theta < 90^\circ$, while the $S_{1,cis}$ has $10^\circ < \theta < 30^\circ$.

## VI. SA2-CASSCF/SVP and OM3-MRCISD ionization energy comparison

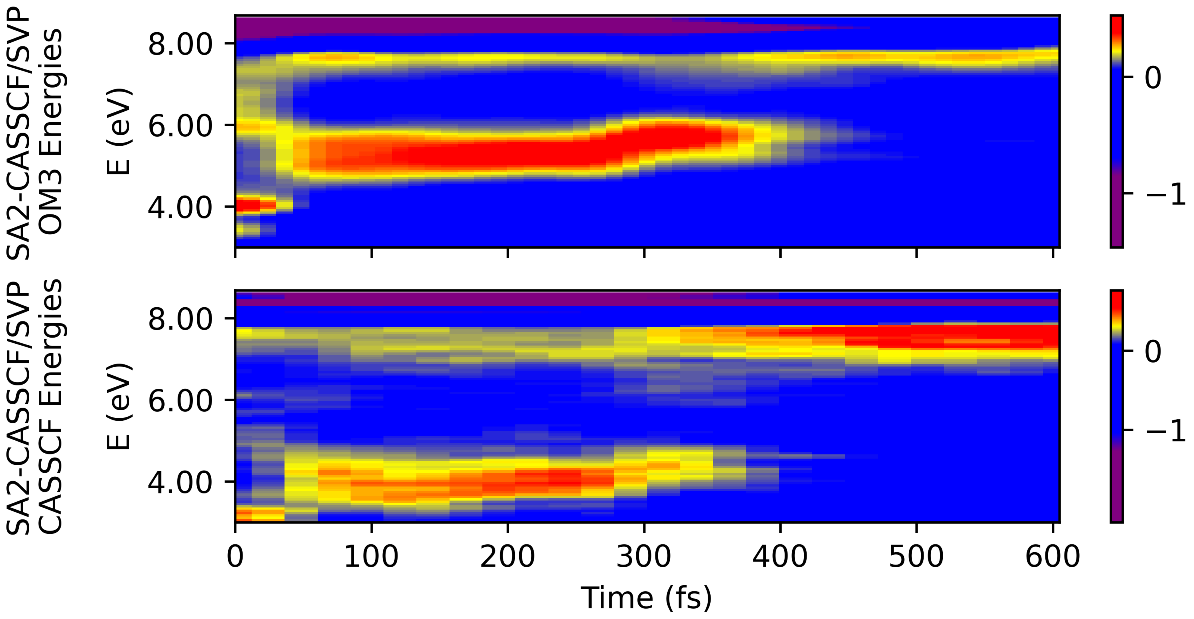


**Figure S8.** Simulated photoelectron spectra for surface hopping dynamics for the SA2-CASSCF/SVP method using ionization energies obtained from two different levels of theory; the intensities were included via Dyson norms. The pump-probe signal was calculated by subtracting the ground state signal at $t = 0$.

## VII. OM3-MRCISD dynamics with FS and LZ surface hopping algorithm comparison

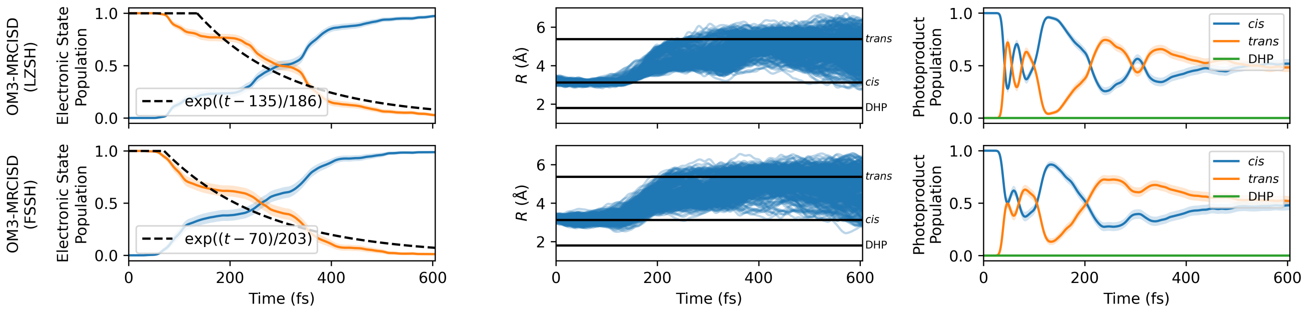


**Figure S9.** Analysis of nonadiabatic simulations for *cis*-stilbene using OM3-MRCISD method using FS and LZ surface hopping algorithm. The left column shows the time evolution of electronic ground and excited-state populations, with accompanying 95% confidence intervals and a least squares fit for the excited-state curve. The middle column tracks the distance $R$ between $C_2$ and $C_{2'}$ atoms throughout the simulation period. We delineate three distinctive distances: the upper one, at 5.375 Å, corresponds to the *trans*-stilbene structure; the middle distance, at 3.125 Å, signifies the *cis*-stilbene configuration; and the distance at 1.8 Å represents the DHP state. The right column showcases the *cis*, *trans*, and DHP photoproduct population during the dynamics. The *trans*-stilbene is defined as a conformation $90° < \theta < 270°$, where $\theta$ is the dihedral angle between atoms $C_1$, $C_0$, $C_{0'}$ and $C_{1'}$. DHP is a conformation with $R < 1.8$ Å, where $R$ is the distance between $C_2$ and $C_{2'}$. Any remaining configurations are classified as *cis*-stilbene.

## VIII. Convergence with number of trajectories

Our high-level dynamics simulations utilize approximately 50 trajectories. This is a much smaller number than what is used for the CASSCF or OM3-MRCISD calculations. The limited number of trajectories translates into larger error bars. To assess the convergence of the dynamics relative to the number of trajectories explicitly, we provide an analysis showing how the results vary with the number of trajectories. This examination helps determine whether the current trajectory count is sufficient for reliable results or if additional trajectories are required to achieve stable and representative dynamics. The data shown in Figure S10 demonstrate that the dynamical outcome is already well-captured within the smaller number of trajectories.

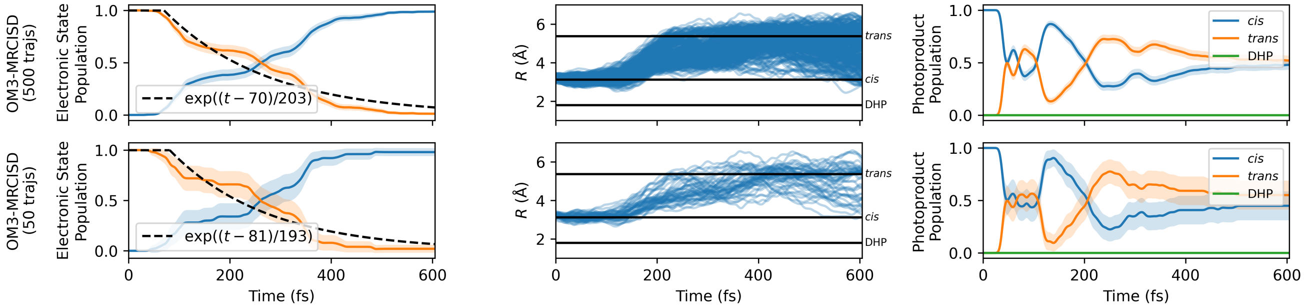


**Figure S10.** Analysis of nonadiabatic simulations for *cis*-stilbene for the OM3-MRCISD method with 500 and 50 trajectories. The left column shows the time evolution of ground and excited-state populations upon the excitation, accompanied by 95% confidence intervals and a least squares fit for the excited-state curve. The middle column tracks the distance $R$ between $C_2$ and $C_{2'}$ atoms throughout the simulation. We delineate three distinctive distances: the upper one, at 5.375 Å, corresponds to the *trans*-stilbene structure; the middle distance, at 3.125 Å, signifies the *cis*-stilbene configuration; and the distance at 1.8 Å represents the DHP state. The right column showcases the *cis*, *trans*, and DHP quantum yields during the dynamics. The *trans*-stilbene is defined as a conformation $90° < \theta < 270°$, where $\theta$ is the dihedral angle between atoms $C_1$, $C_0$, $C_{0'}$ and $C_{1'}$. DHP is a conformation with $R < 1.8$ Å, where $R$ is the distance between $C_2$ and $C_{2'}$. Any remaining configurations are classified as *cis*-stilbene.

## IX. Comparison of simulations including the failed trajectories in the excited state

One technical problem that complicates the comparison between different methods stems from failed simulations, i.e., simulations that for any reason crash at some point and do not reach the final simulation time. Such failures are common in nonadiabatic dynamics as the electronic structure methods are often prone to convergence issues at some parts of the configuration space. The electronic populations are calculated by excluding trajectories that fail in the excited state, ensuring that only complete trajectories contribute to the reported populations. The trajectories failing in the ground state were considered in the ground state till the rest of the simulation. To examine any potential bias introduced by this choice, we present an alternative analysis here. We include the trajectories that failed in the excited state in this alternative analysis, assuming they remain in the excited state (and conformation) until the final simulation time. The results shown in Figure S11 demonstrate a minimum effect of the analysis protocol on all the observed quantities for SA2-CASSCF.

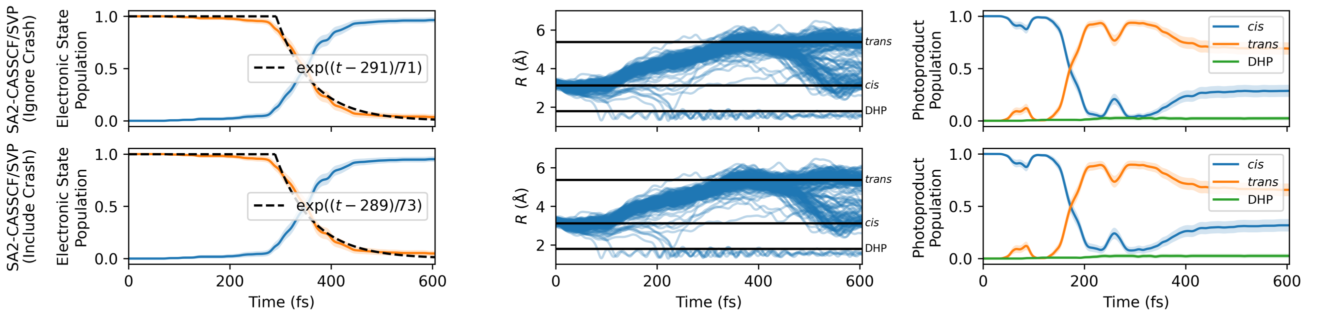


**Figure S11.** Analysis of nonadiabatic simulations for *cis*-stilbene at the SA2-CASSCF/SVP level of theory, where the monitored quantities were calculated with different analysis protocols. The first row corresponds to the method used in the main text (trajectories which failed in the excited state are ignored), whereas the second row corresponds to the protocol including trajectories that failed in the excited state, considering them in the excited state for the rest of the simulation. The left column shows the time evolution of ground and excited electronic state populations upon the excitation, with accompanying 95% confidence intervals and a least squares fit for the excited-state curve. The middle column tracks the distance $R$ between $C_2$ and $C_{2'}$ atoms throughout the simulation period. We delineate three distinctive distances: the upper one, at 5.375 Å, corresponds to the *trans*-stilbene structure; the middle distance, at 3.125 Å, signifies the *cis*-stilbene configuration; and the distance at 1.8 Å represents the DHP state. The right column showcases the *cis*, *trans*, and DHP quantum yields during the dynamics. The *trans*-stilbene is defined as a conformation $90^\circ < \theta < 270^\circ$, where $\theta$ is the dihedral angle between atoms $C_1$, $C_0$, $C_{0'}$ and $C_{1'}$. DHP is a conformation with $R < 1.8$ Å, where $R$ is the distance between $C_2$ and $C_{2'}$. Any remaining configurations are classified as *cis*-stilbene.

# X. The effect of initial conditions: Wigner versus Boltzmann momenta

In the main text, we combine the harmonic Wigner distribution of the positions with momenta sampled from the Boltzmann distribution. This choice was motivated to suppress potential problems with the zero-point leakage. To explore the sensitivity of the results to different initial conditions, we performed an additional simulation using the Wigner distribution also for momenta, a common technique in the field of nonadiabatic dynamics. This comparison allows us to assess how initial conditions influence the overall dynamics and outcomes of the simulations. Demonstrating the effect with the OM3-MRCISD level of theory, we observe that the dynamics are generally unaffected by the choice of initial conditions, see Figure S12.

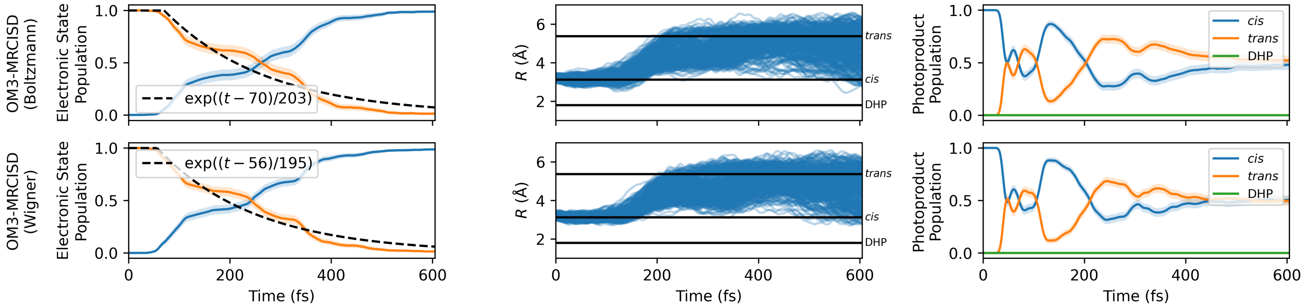


**Figure S12.** Analysis of nonadiabatic simulations for *cis*-stilbene for the OM3-MRCISD level of theory, with different initial conditions. Simulation in the first row used initial momenta generated from the Boltzmann distribution, whereas the second row was initiated with momenta from the Wigner distribution. The left column shows the time evolution of ground and excited-state populations upon the excitation, with accompanying 95% confidence intervals and a least squares fit for the excited-state curve. The middle column tracks the distance $R$ between $C_2$ and $C_{2'}$ atoms throughout the simulation period. We delineate three distinctive distances: the upper one, at 5.375 Å, corresponds to the *trans*-stilbene structure; the middle distance, at 3.125 Å, signifies the *cis*-stilbene configuration; and the distance at 1.8 Å represents the DHP state. The right column showcases the *cis*, *trans*, and DHP quantum yields during the dynamics. The *trans*-stilbene is defined as a conformation $90^\circ < \theta < 270^\circ$, where $\theta$ is the dihedral angle between atoms $C_1$, $C_0$, $C_{0'}$ and $C_{1'}$. DHP is a conformation with $R < 1.8$ Å, where $R$ is the distance between $C_2$ and $C_{2'}$. Any remaining configurations are classified as *cis*-stilbene.
